# Lithium Dihydropyridine Dehydrogenation Catalysis: A Group 1 Approach to the Cyclization of Diamine Boranes

**DOI:** 10.1002/anie.201610905

**Published:** 2016-12-21

**Authors:** Ross McLellan, Alan R. Kennedy, Samantha A. Orr, Stuart D. Robertson, Robert E. Mulvey

**Affiliations:** ^1^ WestCHEM, Department of Pure and Applied ChemistryUniversity of StrathclydeGlasgowG1 1XLUK

**Keywords:** cyclization, diaminoboranes, dihydropyridine, homogeneous catalysis, lithium

## Abstract

In reactions restricted previously to a ruthenium catalyst, a 1‐lithium‐2‐alkyl‐1,2‐dihydropyridine complex is shown to be a competitive alternative dehydrogenation catalyst for the transformation of diamine boranes into cyclic 1,3,2‐diazaborolidines, which can in turn be smoothly arylated in good yields. This study established the conditions and solvent dependence of the catalysis through NMR monitoring, with mechanistic insight provided by NMR (including DOSY) experiments and X‐ray crystallographic studies of several model lithio intermediates.

Main‐group chemistry is still in the early days of an exciting period of reassessment and reinvention owing primarily to the realization that its compounds can act in catalytic roles previously considered the exclusive domain of transition‐metal or lanthanide complexes.^1^ Emerging from years of curiosity‐driven research that uncovered novel structures and bonding, main‐group (pre)catalysts have recently found application in a range of important catalytic processes, including hydroamination,^2^ hydroboration and hydrosilylation,^3^ hydrogenation,^4^ and dehydrogenative element–element bond formation.^5^ The last named is particularly relevant since the catalytic control of dehydrocoupling processes (e.g., formation of B−N bonds) has relevance to hydrogen storage^6^ as well as utility in the synthesis of polymers and preceramic materials.^7^ The research groups of Harder,^8^ Hill,^9^ Wright,^10^ and Okuda,^11^ amongst others,^5^ have reported main‐group d^0^ complexes that are active in stoichiometric and catalytic dehydrocoupling processes; these complexes have contained mostly Group 2 or 13 metals (e.g., Mg, Ca, or Al) and bulky β‐diketiminato or (silyl)amide ligands. Despite this variety of active precatalysts, Group 1 based complexes have remained largely underexplored, which is surprising, since Group 1 precatalysts offer the advantage over Group 2 complexes that they are not involved in Schlenk‐type equilibria,3d though they can engage in aggregation phenomena.

A rare report of Group 1 precatalysts came from Hill and co‐workers, who described the dehydrocoupling of dimethylamine borane by MHMDS precatalysts (M=Li, Na, K; HMDS=1,1,1,3,3,3‐hexamethyldisilazide).^12^ This significant study revealed that the dehydrocoupling process, although competent, suffers from solubility issues due to insoluble M–H aggregates that slow the process. Thus, it follows that the solubility of catalytically critical M–H intermediates is essential for high conversion and efficient reactivity. Recently, we reported the synthesis of metal hydride surrogate complexes in 1‐lithio‐2‐alkyl‐1,2‐dihydropyridines, DHPs, prepared readily through stoichiometric reactions between pyridine and alkyl lithium reagents (cheap everyday laboratory chemicals).^13^ Moreover, 1‐lithio‐2‐*tert*‐butyl‐1,2‐dihydropyridine, **1t** Li (Scheme 1 A) proved soluble in hydrocarbon solvents, an essential synthetic advantage over insoluble polymeric Li−H species.13a,13b Further, **1t** Li proved effective in control hydrometalation reactions with benzophenone,13a thus indicating the dual role of 2‐*tert*‐butylpyridine as a hydrocarbon‐soluble Li−H storage/release vessel.

**Scheme 1:**
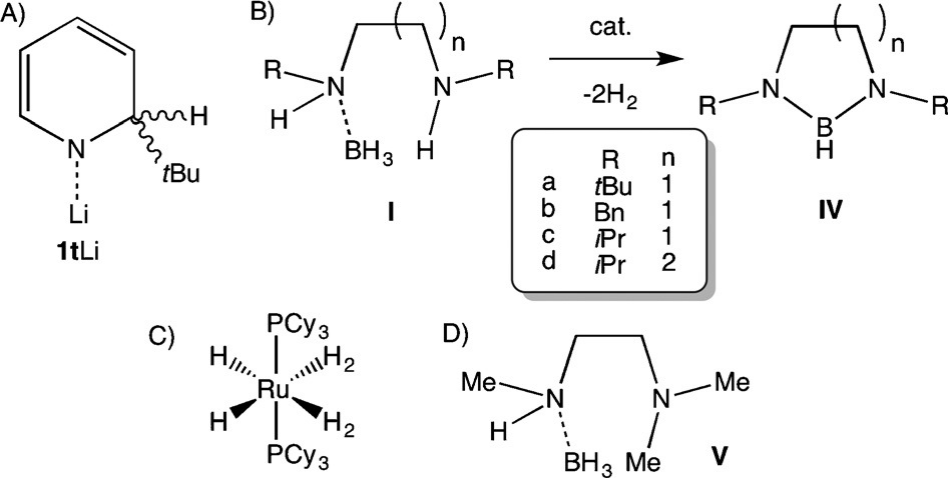
A) Empirical structure of 1‐Li‐2‐*tert*‐butyl‐1,2‐dihydropyridine (**1t** Li). B) Conversion of diamine boranes **I** into 1,3,2‐diazaborolidines **IV**. C) [RuH_2_(η^2^‐H_2_)_2_(PCy_3_)_2_] precatalyst used by the Sabo‐Etienne and Alcaraz groups. D) *N*‐(Methyl)(BH_3_)‐*N′*‐dimethylethylenediamine (**V**). Cy=cyclohexyl.

Since the ability to solubilize Li−H appears paramount in Group 1 dehydrocoupling applications, we decided to test the soluble Li−H surrogate **1t** Li as a precatalyst in the dehydrogenative cyclization of diamine boranes to 1,3,2‐diazaborolidines (Scheme 1 B). This transformation has thus far only been performed with the relatively expensive and (comparatively) synthetically challenging ruthenium‐based precatalyst [RuH_2_(η^2^‐H_2_)_2_(PCy_3_)_2_] (Scheme 1 C).^14^ With this catalyst, Sabo‐Etienne, Alcaraz, and co‐workers successfully demonstrated the cyclization of a selection of diamine boranes in 25–88 % yield at room temperature in THF. Furthermore, it was discovered that bis‐σ‐borane ruthenium complexes are critical intermediates in the cyclization process, which reportedly proceeds through sequential B−H and N−H activation steps.

Herein we report the ability of an inexpensive and readily prepared 1‐metallo‐1,2‐dihydropyridine to act in a catalytic regime. This rare example of a Group 1 based precatalyst displayed competitive reactivity with the aforementioned transition‐metal‐based system. We also discuss the crucial role of DHP/pyridine in the dehydrocoupling process.

1,3,2‐Diazaborolidines are attractive synthetic targets, being nitrogen‐containing analogues of pinacol or catechol borane reagents, which are compounds of widespread utility in hydroboration reactions^15^ and in the synthesis of boronic esters for Suzuki–Miyaura cross‐coupling.^16^ Moreover, boryl reagents, related to 1,3,2‐diazaborolidines through the formal removal of H, have been employed as boron nucleophiles in a manner akin to that of their isoelectronic congeners, singlet N‐heterocyclic carbenes, acting as strong σ‐donors.^17^ One notable area in which 1,3,2‐diazaborolidines have found application is in the asymmetric reduction of prochiral ketones.^18^

Initially, we selected *N*‐(*tert*‐butyl)(BH_3_)‐*N′*‐*tert*‐butylethylenediamine (**Ia**; Scheme 1 B) for our investigations, since it can be readily prepared by the reaction of BH_3_⋅SMe_2_ with the parent diamine. Previous work revealed the effective catalytic cyclization of **Ia** at room temperature in THF to afford *N*,*N′*‐di‐*tert*‐butyl‐1,3,2‐diazaborolidine (**IVa**) in 88 % yield after 16 h.14b Replication of the experimental conditions (THF at room temperature) in a J. Young NMR tube with [D_8_]THF and **1t** Li (5 mol %) gave only very slow conversion, as evidenced by ^1^H and ^11^B NMR monitoring; however, it was observed that the addition of the catalyst resulted in immediate gas evolution. Heating of the reaction at 70 °C resulted in much faster conversion into **IVa** (>97 % in situ NMR yield; Table 1). Next, we probed the effect of the reaction solvent on the cyclization process. Switching from THF to [D_6_]benzene enabled us to isolate **IVa** in over 94 % yield after 6 h at 70 °C; thus, the reaction was four times faster than in THF. Isolation from the catalyst residue was achieved by distillation (see Figure S3 in the Supporting Information). The use of [D_5_]pyridine had a dramatic effect on both the reaction timescale and product formation with **1t** Li (5 mol %); **Ia** was essentially fully consumed after just 45 min. ^1^H NMR data revealed two products in an approximate 3:2 ratio, each of which gave rise to signals consistent with a symmetric ethylenediamine backbone. A control reaction of **Ia** in [D_5_]pyridine with no catalyst confirmed the identity of the minor component. After 2 h at 70 °C, essentially complete transfer of BH_3_ from **I** to pyridine had occurred, thus leaving *N*,*N′*‐di‐*tert*‐butylethylenediamine (see Figure S5). The relevant resonance in the ^11^B NMR spectrum was a quartet at −11.2 ppm.^19^ Both ^1^H and ^11^B NMR spectra showed that this compound was the minor component of the product mixture. The resonances of the major component of the catalytic reaction were consistent with the cyclized product **IVa**, as manifested by a doublet at 26.3 ppm, albeit containing a shoulder, which indicated a mixture of products. Diffusion‐ordered spectroscopy (DOSY) NMR studies^20^ failed to provide any additional insight (see Figure S6). Thus, in bulk pyridine, these reactions proceed at an accelerated rate, but also form undesired side products.

**Table 1:** Catalytic conversion of **I** into **IV** with the precatalyst **1t** Li (5 mol %).

Substrate	Solvent	*t* [h]	*T* [°C]	Yield^[a]^
**Ia**	[D_8_]THF	24	70	97
**Ia**	[D_6_]benzene	6	70	94^[b]^
**Ia**	[D_5_]pyridine	0.75	70	57^[c]^
**Ib**	[D_6_]benzene	24	70	96
**Ic**	[D_6_]benzene	48	70	77
**Id**	[D_6_]benzene	48	70	97

[a] Conversion determined by ^11^B NMR spectroscopy. [b] Yield of the isolated product. [c] A mixture of products was obtained; the second product was identified as the parent diamine, which formed as a result of BH_3_ transfer to pyridine.

Under these optimal reaction conditions, a variety of similar substrates **Ib**–**d** gave the desired products **IVb**–**d** in good yields (Table 1), in excess of those achievable by Ru catalysis, thus highlighting the versatility of this approach. With the optimum solvent benzene, we probed the mechanism of this reaction by recording NMR spectra in the lead up to reaction completion. Figure 1 A displays the ^11^B NMR spectra taken at various intervals during the reaction. At the initial time point (prior to heating), three species existed. The major resonance, a quartet at −19.2 ppm, corresponds to **Ia** and partially obscures a second lower‐intensity quartet at −21.7 ppm, which we assign to a species **IIa** resulting from initial deprotonation of an acidic N−H group. This step is in agreement with the accepted mechanism for the dehydrocoupling of HNMe_2_⋅BH_3_.9a Finally, a low‐intensity doublet at 26.2 ppm is attributed to the B−H group of the cyclization product **IVa**. After heating for 2 h, as well as a clear increase in product/decrease in starting material, four other low‐intensity intermediates were observed: a quintet at *δ*=−38.8 ppm assigned to LiBH_4_; a quartet at *δ*=−13.4 ppm (the identity of which remains unknown; however, it does not correspond to 2‐*t*Bu‐py⋅BH_3_, see Figure S10); and a pair of triplets at *δ*=−5.8/38.0 ppm, which we assign to a dimer/monomer equilibrium of **IIIa**. Sabo‐Etienne and co‐workers reported a related dimer/monomer equilibrium (R=Me, *n*=5) at 4/38 ppm in the ^11^B NMR spectrum.14b

**Figure 1:**
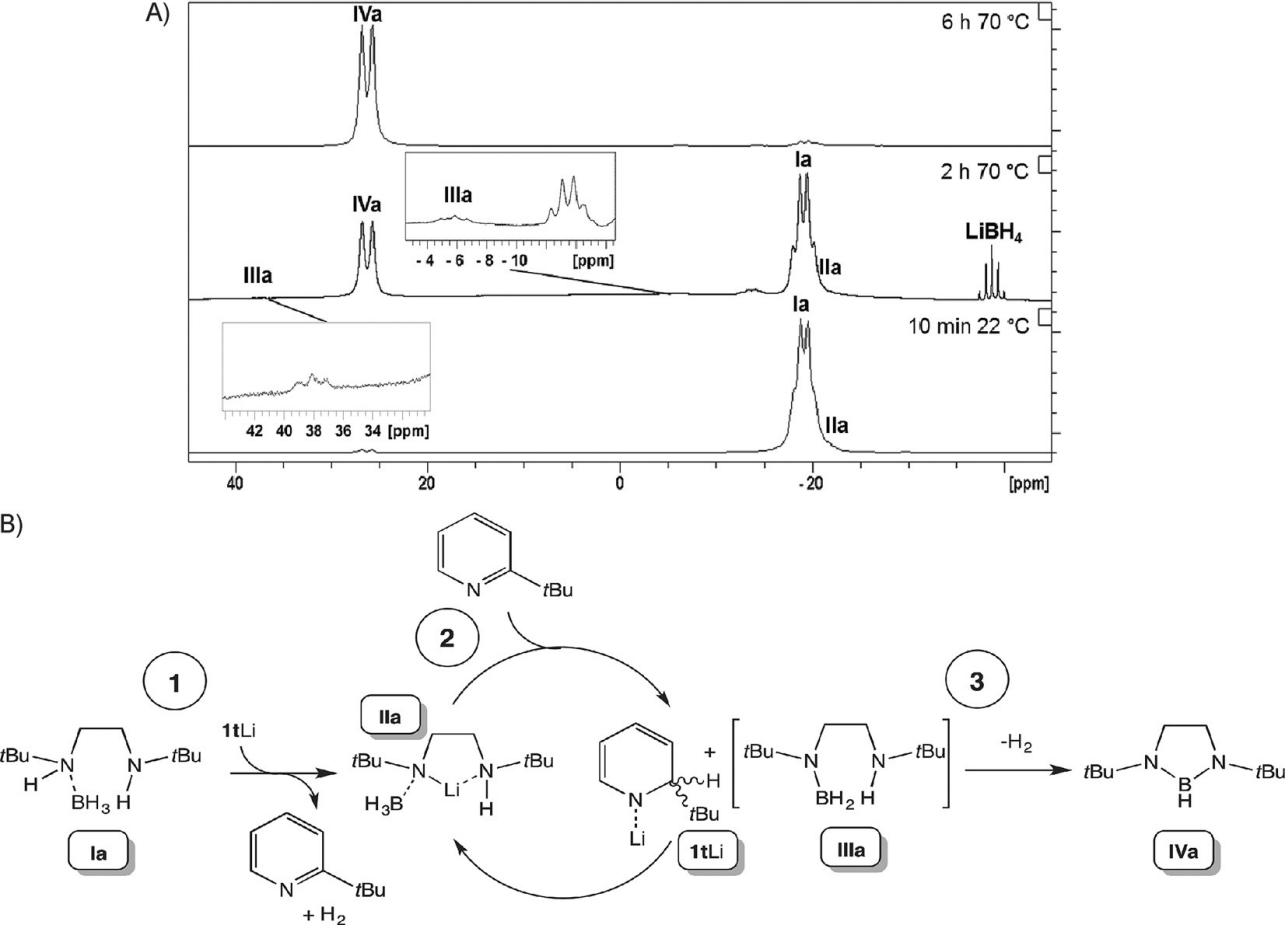
A) ^11^B NMR spectra of the reaction between **1t** Li (5 mol %) and *N*‐(*tert*‐butyl)(BH_3_)‐*N′*‐*tert*‐butylethylenediamine (**Ia**) in [D_8_]THF. B) Proposed catalytic cycle for the conversion of diamine boranes into 1,3,2‐diazaborolidines, using **Ia** as a representative example.

These observations suggest that the catalysis proceeds by three key steps (Figure 1 B): 1) deprotonative metalation to form **II** with the concomitant formation of H_2_ and 2‐*tert*‐butylpyridine, in contrast to the mechanism proposed for the Ru system (successive B−H and N−H activation); 2) following our hypothesis, β‐hydride elimination to give **III** with the addition of Li−H over the 2‐*tert*‐butylpyridine generated in situ to regenerate an active 1‐lithio‐2‐*tert*‐butyldihydropyridine species (**1t** Li or one of its isomers, see below); 3) intramolecular loss of H_2_ to generate **IV**.^21^

An explanation for the presence of LiBH_4_ is that the reaction between **1t** Li and **Ia** (step 1) may also proceed through a minor secondary pathway involving the reaction of BH_3_ with the Li−H source to generate LiBH_4_ and 2‐*tert*‐butylpyridine.^22^ Presumably, this pathway is slower than the deprotonation step and so only minor amounts are formed. The ^1^H NMR data agree well with the ^11^B NMR data. The faster reaction rate in benzene with respect to THF can be explained by THF solvation of Li in step 2, which slows the β‐hydride elimination step. We next set out to obtain structural evidence for the initial reaction product between **1t** Li and **Ia**. In parallel, we also included *N*‐(methyl)(BH_3_)‐*N*′‐dimethylethylenediamine (**V**) as a substrate, since one N atom does not possess an acidic H atom and is thus incapable of cyclizing. Gratifyingly, direct stoichiometric reactions of **Ia** and **V** with **1t** Li in *n*‐hexane afforded single crystals in four cases after the addition of THF or pyridine: ([(*t*Bu)N(BH_3_)(CH_2_)_2_N(H)(*t*Bu)Li⋅THF] (**IIa⋅THF**; Figure 2 A), [(*t*Bu)N(BH_3_)(CH_2_)_2_N(H)(*t*Bu)Li⋅py], (**IIa⋅py**), [{(Me)N(BH_3_)(CH_2_)_2_N(Me)_2_Li⋅THF}_2_] (**[(VI⋅THF)_2_]**; Figure 2 B), and [{(Me)N(BH_3_)(CH_2_)_2_N(Me)_2_Li⋅py}_2_] (**[(VI⋅py)_2_]**). Reactions were low‐yielding (24–34 % yield of the isolated crystalline product), a probable consequence of 2‐*tert*‐butylpyridine causing further reaction.

**Figure 2:**
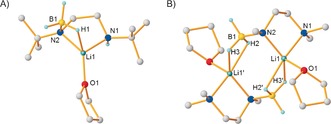
Molecular structures of A) **IIa⋅THF** and B) **[(VI⋅THF)_2_]**; transformations used to generate symmetry‐equivalent atoms: −*x*,1−*y*,−*z*. All hydrogen atoms except those attached to boron and nitrogen atoms are omitted for clarity. Structures of **IIa⋅py** and **[(VI⋅py)_2_]** and selected bond lengths and angles of all structures are given in the Supporting Information.

A higher‐yielding procedure was found in the direct treatment of **Ia** or **V** with *n*BuLi (48–81 %, Scheme 2). **IIa⋅THF** and **IIa⋅py** are essentially isostructural except for the donor solvating Li; thus, only **IIa⋅THF** is discussed herein. Its structure is best described as a distorted trigonal‐pyramidal Li complex with one site occupied by a BH_3_ hydridic H⋅⋅⋅Li interaction (the three H atoms bonded to boron were located during refinement). Coordination is completed by a THF molecule and a deprotonated molecule of **I**, which binds in a bidentate fashion through its amino and amido N atoms (see Figure S13 for relevant structural parameters). Similarly, **[(VI⋅THF)_2_]** and **[(VI⋅py)_2_]** differ only in solvation, and hence only one structure is discussed herein. **[(VI⋅THF)_2_]** is a centrosymmetric dimer with the main difference to **IIa⋅THF** being two hydridic H⋅⋅⋅Li interactions (all three H atoms bonded to boron were located) between the symmetry‐equivalent moieties comprising the dimeric unit; that is, the B−H⋅⋅⋅Li interactions are between the BH_2_ and Li from opposite halves of the molecule. Moreover, one B−H⋅⋅⋅Li distance is notably shorter than the other [H2–Li1′ 2.074(10) Å versus H3–Li1′ 1.962(13) Å]. Li1 exhibits a distorted square‐pyramidal geometry, completed by a THF and a bidentate deprotonated diamine borane ligand.

**Scheme 2:**
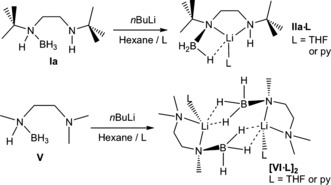
Synthesis of **IIa** and **VI**.

Crucially, in all four cases an acidic N−H functionality has been deprotonated, thus implying that these complexes represent valid reaction intermediates resulting from the first step in the cyclization reaction. Further, the B−H⋅⋅⋅Li interactions [Li−H range (shortest Li−H distance in **VI**) 1.894(18)–1.962(13) Å)] are all somewhat shorter than the shortest Li−H contact in solid Li−H (2.043 Å).^23^ The hydride is thus activated and predisposed to add across pyridine (see pyridine solvates) to regenerate an active catalyst^24^ and leave a N−BH_2_ functionality in close proximity to the remaining N−H hydrogen atom (in the case of **IIa⋅py**). The aggregation states of **IIa⋅THF** and **[(VI⋅THF)_2_]** in [D_8_]THF were assessed in DOSY NMR studies by the Stalke method.20c In each case, the results indicate that the solid‐state arrangement is preserved, albeit without THF of solvation in **[(VI⋅THF)_2_]** (see Figure S15 and S16), thus reflecting the longer Li−O bonds [1.984(2) versus 1.907(3) Å] and the higher coordination number of lithium (5 versus 4), which leads to higher lability of the THF ligand in **[(VI⋅THF)_2_]**.

By exploiting reaction intermediates **IIa** and **VI**, we conducted a series of experiments to probe further the mechanistic details and the crucial role of the reaction solvent (Scheme 3). Heating of **IIa⋅THF** at 70 °C in either [D_6_]benzene or [D_5_]pyridine resulted in cyclization after 70 or 25 h, respectively (Scheme 3 A). Presumably in the former, reaction occurs through elimination of Li−H (a slight amount of precipitate was evident) and thermally induced dehydrogenation. Moreover, the reaction was monitored by ^11^B NMR spectroscopy, which showed a quartet at *δ*=−21.7 and a doublet at *δ*=26.3 ppm corresponding to starting material and product, respectively. In contrast, during catalysis several intermediate species were observed (see above), which may indicate that slow Li−H loss is followed by rapid (as compared with the NMR timescale) dehydrogenative cyclization. Significantly, in [D_5_]pyridine, the reaction appeared to proceed almost three times as fast, which can be explained by the displacement of solvating THF by the bulk deuterated solvent, followed by dehydrogenation and Li−H addition across pyridine. The reaction of **IIa⋅py** (a pyridine solvate preorganized for DHP formation) at 70 °C in [D_6_]benzene resulted in product formation over a similar time, 22 h. Crucially, ^1^H NMR data recorded periodically during the experiment revealed several resonances attributable to DHP species, albeit with low integration values.

**Scheme 3:**
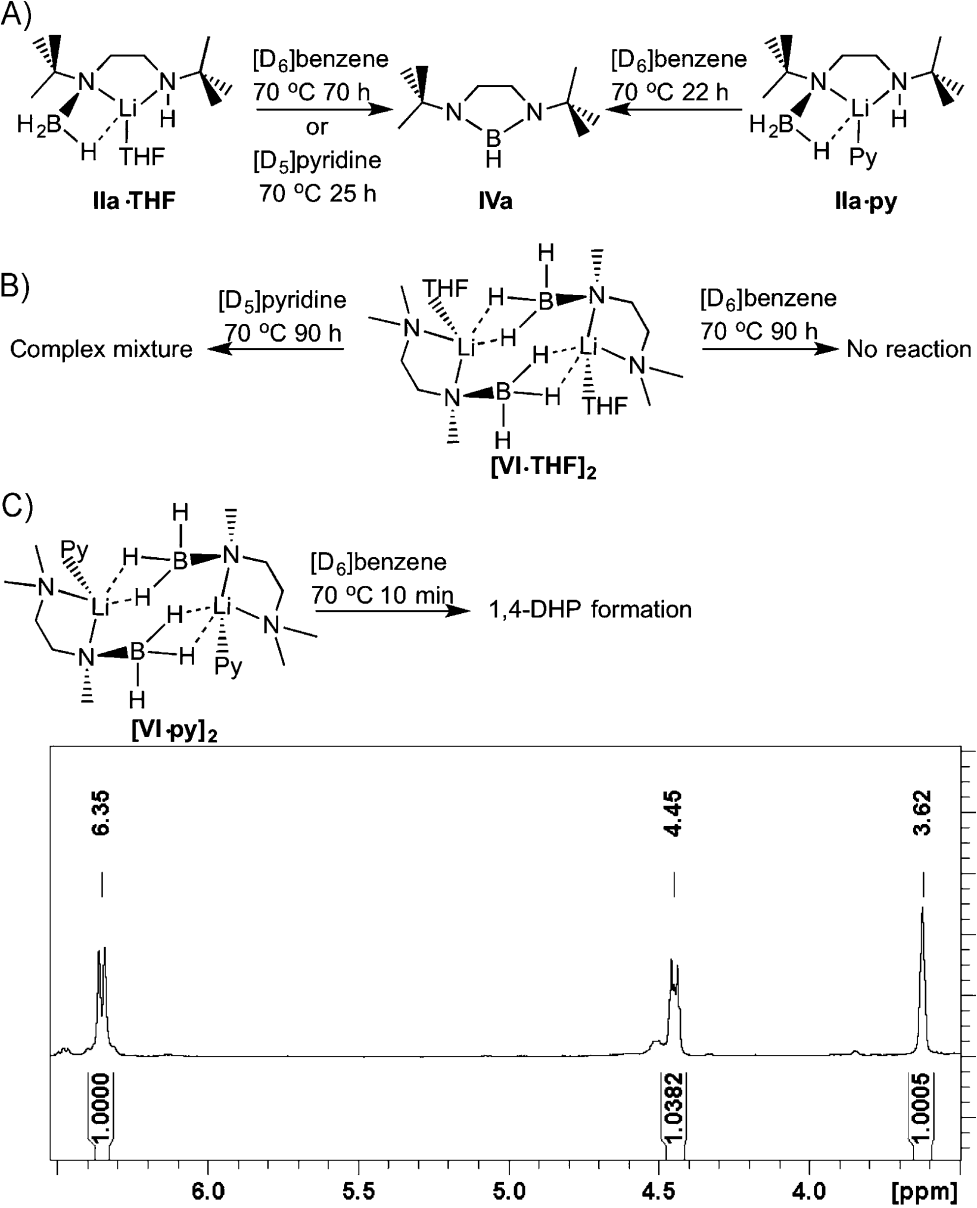
A) Reaction of intermediates **IIa⋅THF** and **IIa⋅py** in [D_6_]benzene or [D_5_]pyridine. B) Reaction of intermediate **[**(**VI⋅THF)_2_]** in [D_6_]benzene or [D_5_]pyridine. C) Reaction of intermediate **[**(**VI⋅py)_2_]** in [D_6_]benzene to give a 1,4‐DHP species. Py/py=pyridine.

Since **[(VI⋅THF)_2_]** and **[(VI⋅py)_2_]** lack an acidic H atom bound to one N atom, they are unable to cyclize as **IIa**, thus giving an opportunity to probe the role of pyridine in the reaction after the initial deprotonation. When the THF solvate **[(VI⋅THF)_2_]** in [D_6_]benzene was heated at 70 °C for 90 h (Scheme 3 B), it remained unchanged as anticipated, thus implying that Li−H is not readily eliminated under these forcing conditions. Heating of **[(VI⋅THF)_2_]** at 70 °C for 90 h in [D_5_]pyridine resulted in complete consumption of the starting material and ultimately in a complex mixture of products (see Figure S25). Heating of **[(VI⋅py)_2_]** at 70 °C in [D_6_]benzene resulted in only a small change in the ^1^H and ^11^B NMR spectra after 10 min (Scheme 3 C), consistent with a 1,4‐dihydropyridine species (see Figure S27).^25^ Heating at 70 °C for 72 h resulted in no further change in the spectra.

Next, we investigated the postsynthetic functionalization of **IVa** by treatment with phenyllithium in situ after formation by the described catalytic method (Figure 3 A; see the Supporting Information for full details). Monitoring by ^1^H and ^11^B NMR spectroscopy revealed the formation of a new B−C_phenyl_ bond at room temperature, with approximately 80 % conversion (determined by ^11^B NMR spectroscopy) after 2 h. The ^11^B NMR spectrum displayed a diagnostic singlet resonance at *δ=*31.8 ppm. Similar reactivity has recently been observed by Nozaki and co‐workers.^26^ Replacement of the hydridic hydrogen atom with a phenyl group (to give **IVa Ph**) importantly provided facile access to a new nitrogen‐based boronate ester with potential for exploitation in Suzuki–Miyaura cross‐coupling reactions.^27^ **IVa Ph** was readily isolated by conducting the initial catalysis in toluene prior to the addition of *n*‐hexane and cooling to −68 °C. In this way, single crystals were obtained in 70 % yield. The structure (Figure 3 B) confirms the replacement of the boron hydride with a boron–carbon bond. Notably, the phenyl group exists in an orthogonal disposition to the mean plane of the diazaborolidine ring (dihedral angle: 85°), partly as a consequence of the proximity of the bulky *t*Bu substituents attached to the nitrogen atoms. The generality of this reaction was probed with compounds **IVb**–**IVd**, initially on an NMR scale. **IVb Ph** and **IVc Ph** were readily isolable in yields of 81 and 61 %, respectively. **IVd Ph** was isolated as a waxy solid as an inseparable mixture with the BH precursor.

**Figure 3:**
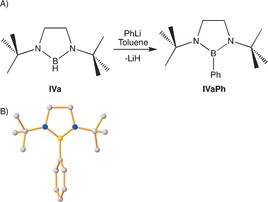
A) Formation of **IVa Ph** by the reaction of **IVa**, generated in situ, with PhLi. B) Molecular structure of **IVa Ph**; transformations used to generate symmetry‐equivalent atoms: −*x*,+*y*,1/2−*z*. All hydrogen atoms are omitted for clarity. Selected bond lengths and angles are given in the Supporting Information.

In summary, this study has introduced Group 1 DHP‐based precatalysts into the catalytic arena, specifically in the cyclization of diamine boranes to give 1,3,2‐diazaborolidines, and assessed the importance of the reaction solvent on their catalytic performance. Spectroscopic and structural studies elucidated the key role in this catalytic regime of 2‐*tert*‐butylpyridine, which is able to store and deliver Li−H on demand. The B−H bond of 1,3,2‐diazaborolidines can be smoothly converted into a B−C bond by the use of PhLi under mild conditions. The study presented herein should assist the development of other new Group 1 catalysts and precatalysts.

## Experimental Section

General catalytic procedure: **I** was dissolved in the required NMR solvent, and NMR spectra were recorded. **1t** Li (5 mol %) was added, and the reaction mixture was heated at 70 °C for the requisite time. Conversion was determined by ^11^B NMR spectroscopy.

Synthesis of **IIa**/**VI**: **Ia** or **V** was dissolved in *n*‐hexane, *n*BuLi was added, and the mixture was stirred for 10 min, during which time a white solid precipitated. THF or pyridine was added dropwise until the solid had completely dissolved. The product crystallized at −20 °C overnight.

## Supporting information

As a service to our authors and readers, this journal provides supporting information supplied by the authors. Such materials are peer reviewed and may be re‐organized for online delivery, but are not copy‐edited or typeset. Technical support issues arising from supporting information (other than missing files) should be addressed to the authors.

Supplementary
