# Supplementary material for: Lithium Dihydropyridine Dehydrogenation Catalysis: A Group 1 Approach to the Cyclization of Diamine Boranes
Source: Angew Chem Int Ed Engl. 2016 Dec 21;56(4):1036–41. doi: 10.1002/anie.201610905 (PMC5396369; doi:10.1002/anie.201610905)
Supplement: Supplementary file 1 — Supplementary [file ANIE-56-1036-s001.pdf]

Angewandte Chemie
Eine Zeitschrift der Gesellschaft Deutscher Chemiker

Supporting Information

Lithium Dihydropyridine Dehydrogenation Catalysis: A Group 1 Approach to the Cyclization of Diamine Boranes

Ross McLellan, Alan R. Kennedy, Samantha A. Orr, Stuart D. Robertson, and Robert E. Mulvey**

anie_201610905_sm_miscellaneous_information.pdf

General experimental considerations

All reactions and manipulations were conducted under a protective argon atmosphere using either standard Schlenk techniques or an MBraun glove box fitted with a gas purification and recirculation unit. NMR experiments were conducted in J. Youngs tubes oven dried and flushed with Argon prior to use. Hexane and THF were dried by heating to reflux over sodium benzophenone ketyl and then distilled under nitrogen prior to use. All other reagents were purchased commercially from Sigma-Aldrich and used as received. **1t**Li,[1] **I** and **V**[2] were prepared as previously described or by slight variations thereof.

NMR Spectroscopy NMR spectra were recorded on a Bruker AV3 or AV 400 MHz spectrometer operating at 400.13 MHz for ^{1}H, 128.38 MHz for ^{11}B, 155.47 MHz for ^{7}Li and 100.62 MHz for ^{13}C. All ^{13}C spectra were proton decoupled. ^{1}H and ^{13}C NMR spectra were referenced against the appropriate solvent signal. ^{7}Li NMR spectra were referenced against LiCl in D_2O at 0.00 ppm and ^{11}B spectra were reference against $BF_3{\cdot}OEt_2$ in $CDCl_3$ at 0.00 ppm

X-ray Crystallography Crystallographic data were collected on Oxford Diffraction instruments with Mo Kα radiation (λ = 0.71073 Å). Structures were solved using SHELXS-97[3] or OLEX2,[4] while refinement was carried out on F2 against all independent reflections by the full matrix least-squares method using the SHELXL-97 program or by the GaussNewton algorithm using OLEX2. All non-hydrogen atoms were refined using anisotropic thermal parameters. Selected crystallographic details and refinement details are provided in table S1. CCDC 1476810-1476813, 1513975 contains the supplementary crystallographic data for this structure. These data can be obtained free of charge from the Cambridge Crystallographic Data Centre via www.ccdc.cam.ac.uk/data_request/cif.

Catalytic dehydrogenative cyclisation of I with 5 mol% 1tLi

Ia (93 mg, 0.5 mmol) and ferrocene (9.3 mg, 0.05 mmol) were placed in a J. Youngs NMR tube and dissolved in either d_8-THF or d_6-benzene and NMR data were recorded. **1t**Li (3.6 mg, 5 mol%) was then added (in the case of d_5-pyridine **1t**Li was added prior to NMR solvent due to an unwanted side reaction). The NMR tube was then heated for the prescribed period and the reaction monitored via ^{1}H, and ^{11}B spectroscopy.

Catalytic dehydrogenative cyclisation reactions of **Ib**-**Id** were conducted using the same procedure.

Figure S1: ^{1}H and ^{11}B NMR spectra of catalytic cyclisation of **1a** in d_8-THF at 70 °C for 24 h.

A) ^{1}H NMR spectra

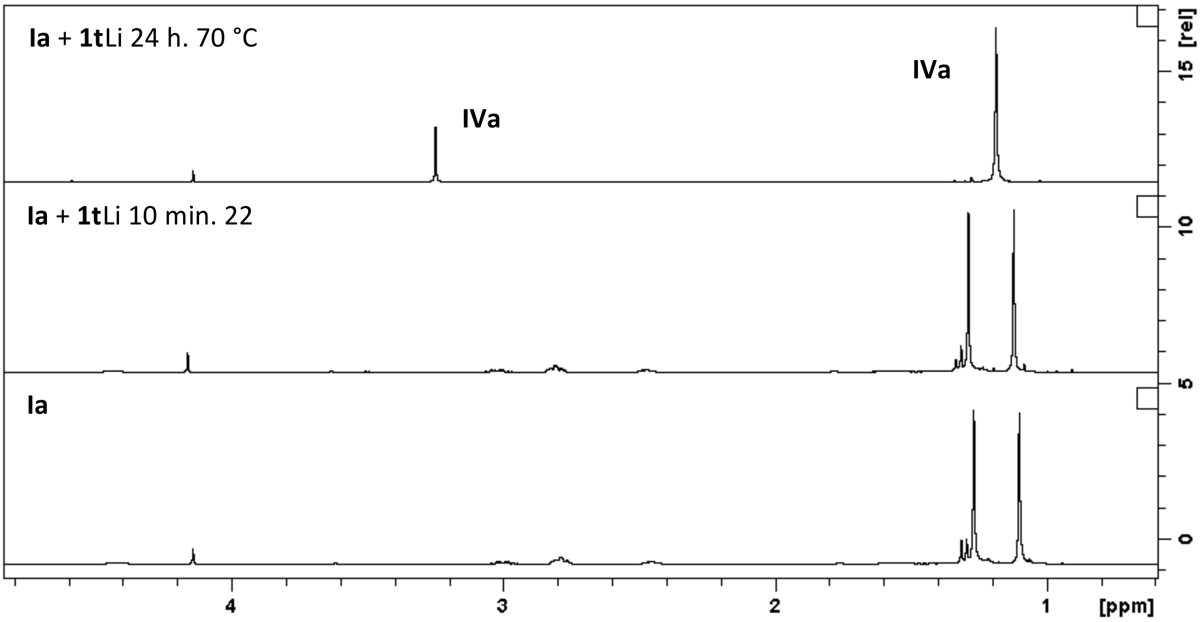

B) ^{11}B NMR spectra

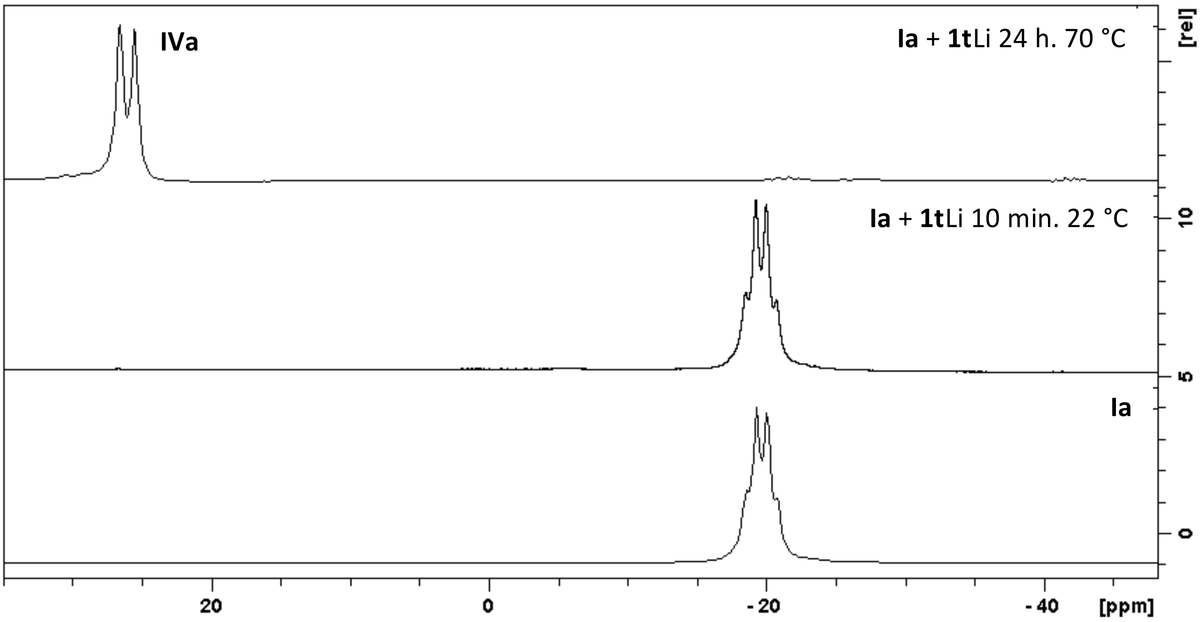

Figure S2: ^{1}H and ^{11}B NMR spectra of catalytic cyclisation in d_6-benzene at 70 °C for 6 h.

A) ^{1}H NMR spectra

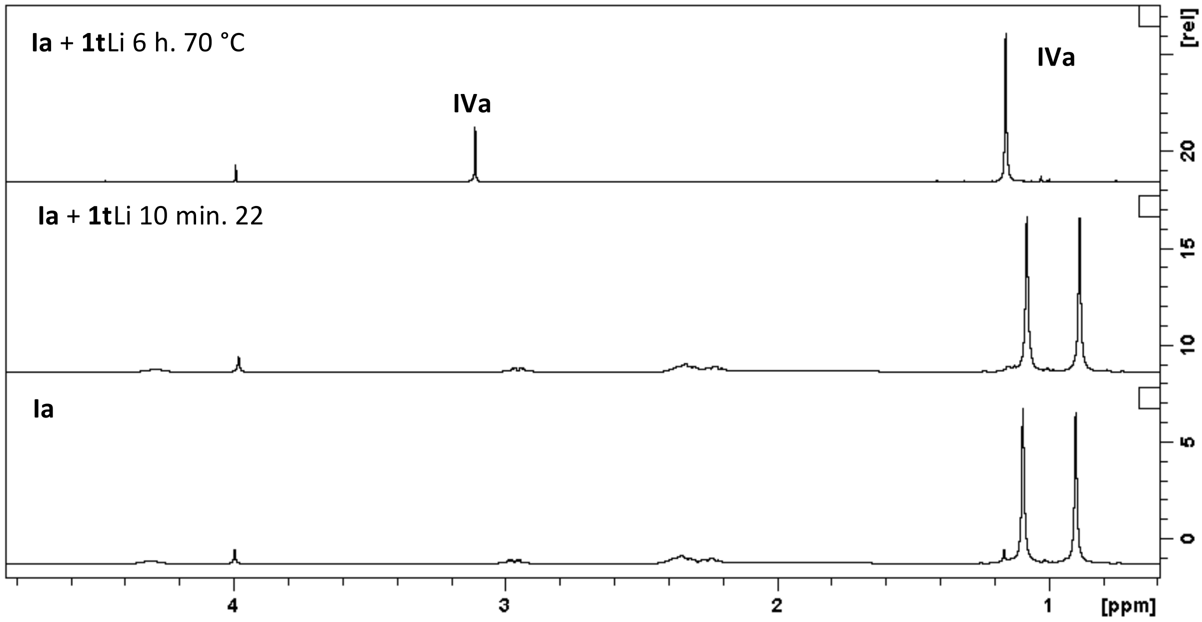

B) ^{11}B NMR spectra

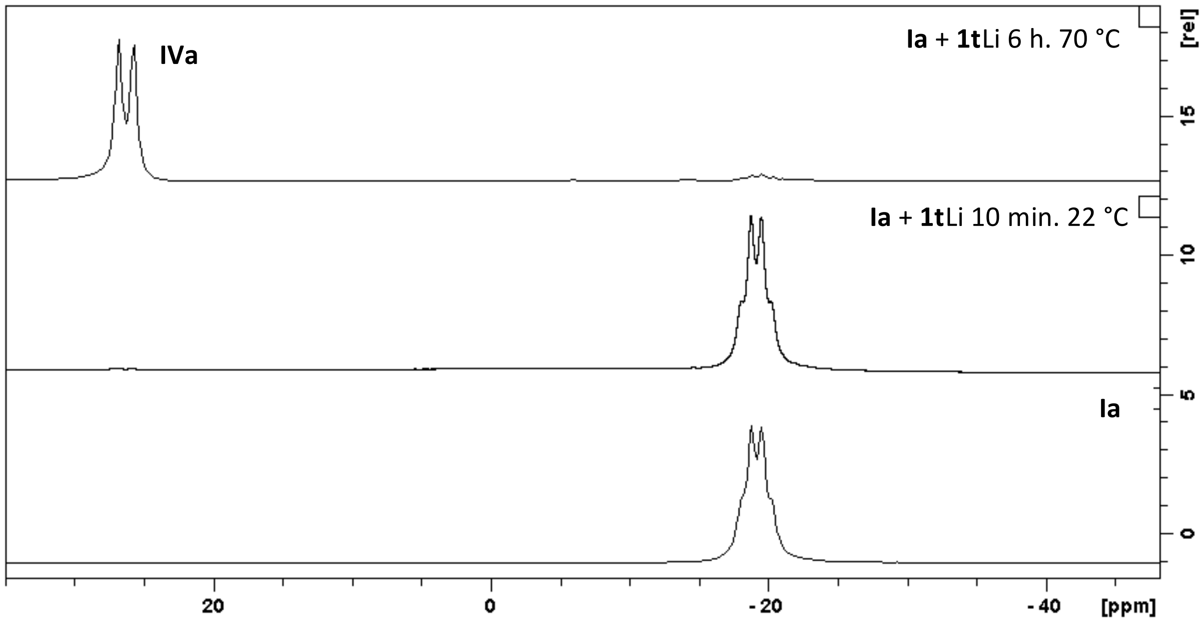

Isolation of **IVa**

Ia (93 mg, 0.5 mmol) and **1t**Li (3.6 mg, 5mol%) were stirred in benzene (1 mL) in a Schlenk flask at 70 °C for 6 h. Benzene was removed by distillation at atmospheric pressure (oil bath temp – 90 °C). A second colourless oil was collected via distillation (oil bath temp 105 °C) and was confirmed by NMR studies as **IVa**. Yield 86 mg, 94%.

^{1}H NMR (400.1 MHz, C_6D_6 300K): δ 3.13 (4H, s, C*H₂*), 1.18 ppm (18H, s, *t*Bu)

^{11}B NMR (128.4 MHz, C_6D_6 300K): δ 25.5 ppm (d, $^{1}J_{B\text{-}H}$ 138.2 Hz, BH) and -18.8 ppm (q, $^{1}J_{B\text{-}H}$ 94.8 Hz, BH_3) corresponding to a small amount of starting diamine borane.

Figure S3 NMR characterisation of **IVa**

A) ^{1}H NMR spectrum

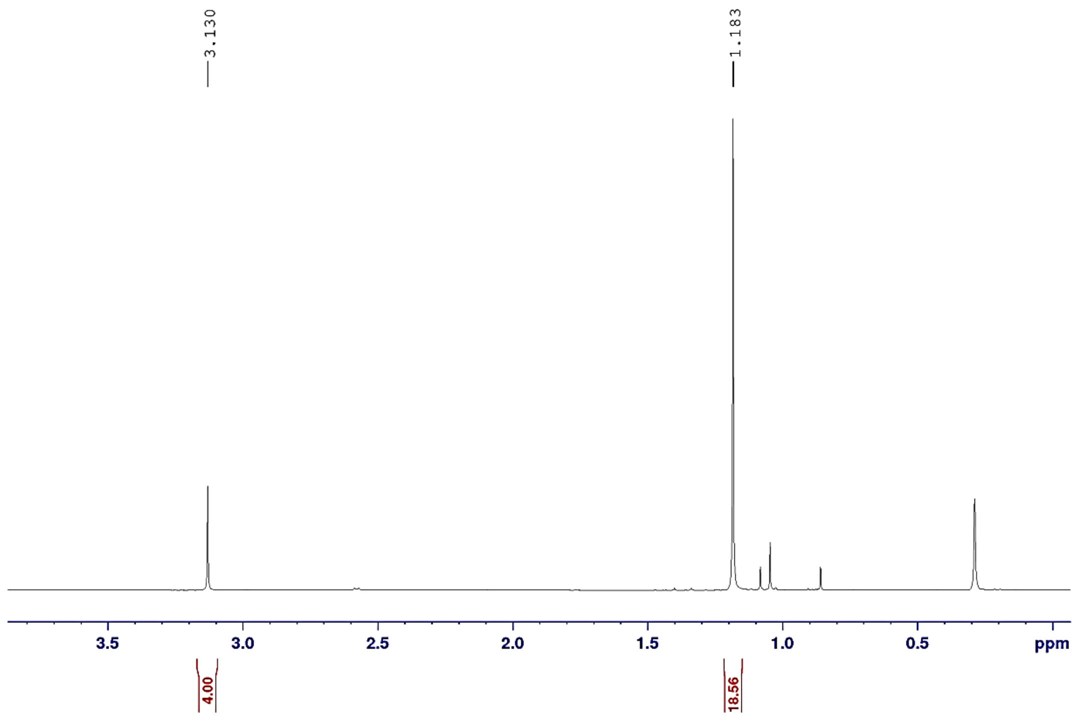

B) ^{11}B NMR spectrum

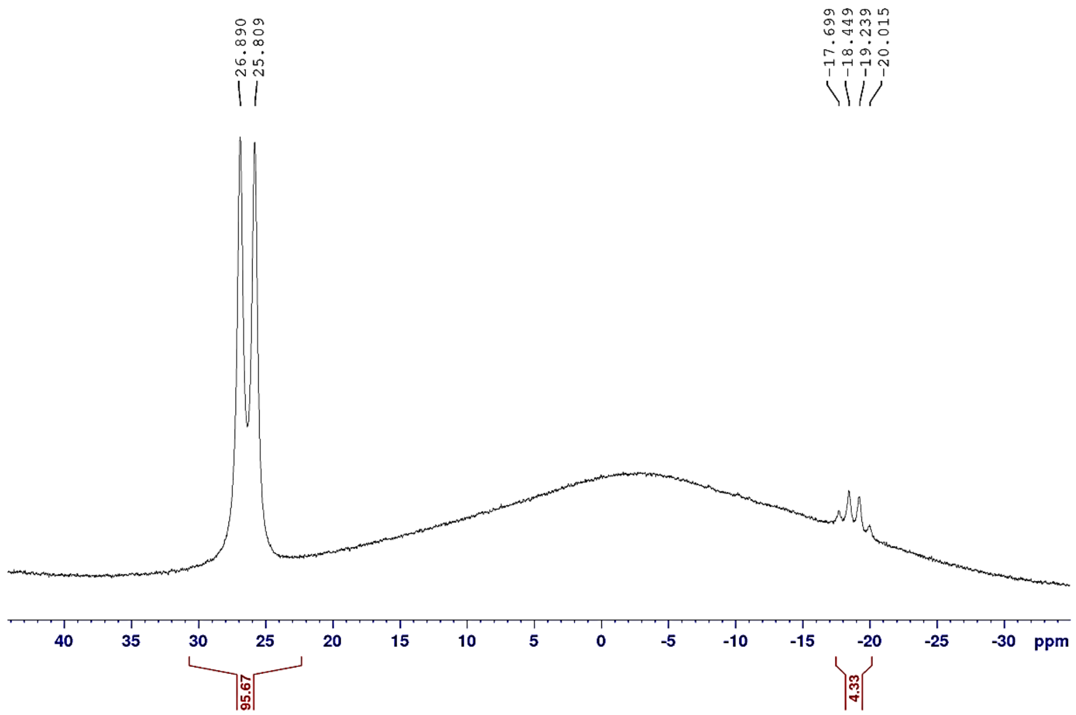

C) ^{7}Li NMR spectrum

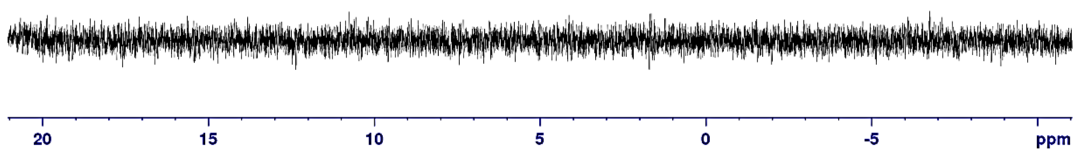

Figure S4: ^{1}H and ^{11}B NMR spectra of catalytic cyclisation in d_5-pyridine at 70 °C for 45 min. Reaction affords a mixture of products due to the transfer of BH_3 from the diamine borane to pyridine, and consequently liberates the parent diamine.

A) ^{1}H NMR spectra

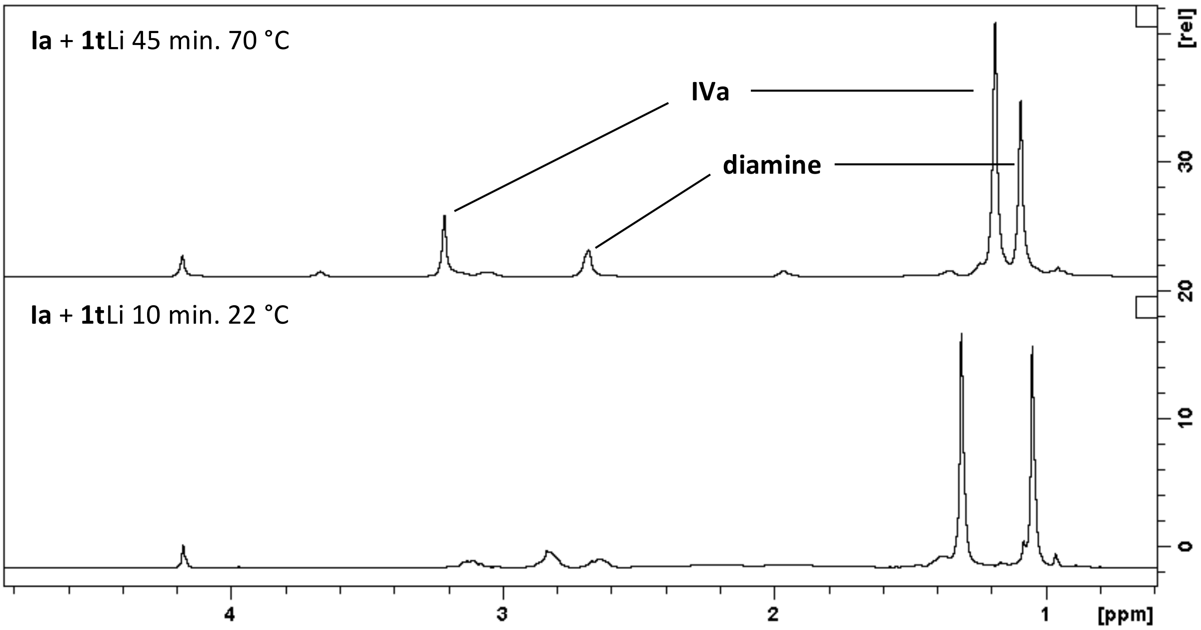

B) ^{11}B NMR spectra

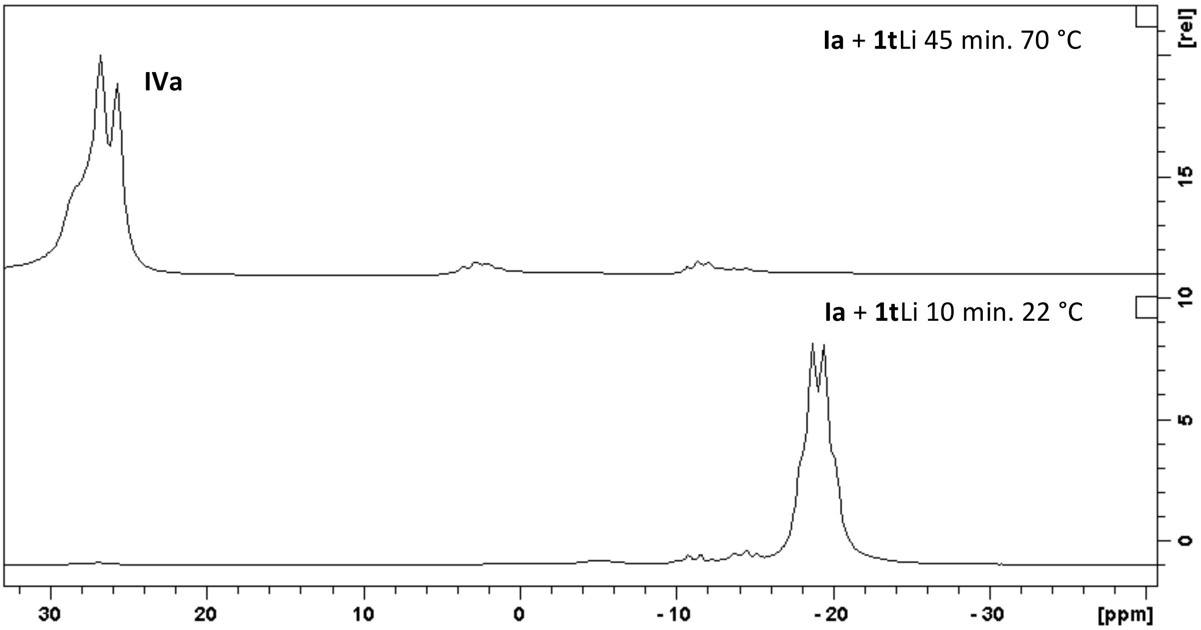

Figure S5 Control reaction of **Ia** in d_5-pyridine demonstrating removal of BH_3 from the diamine borane.

A) ^{1}H NMR spectra

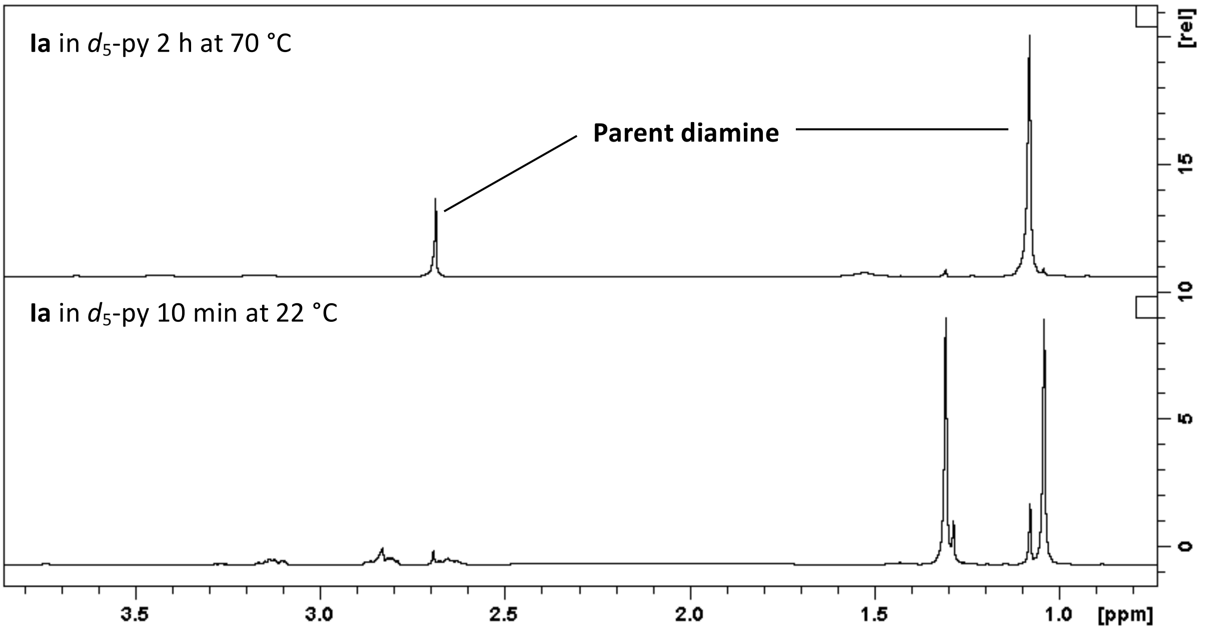

B) ^{11}B NMR spectra

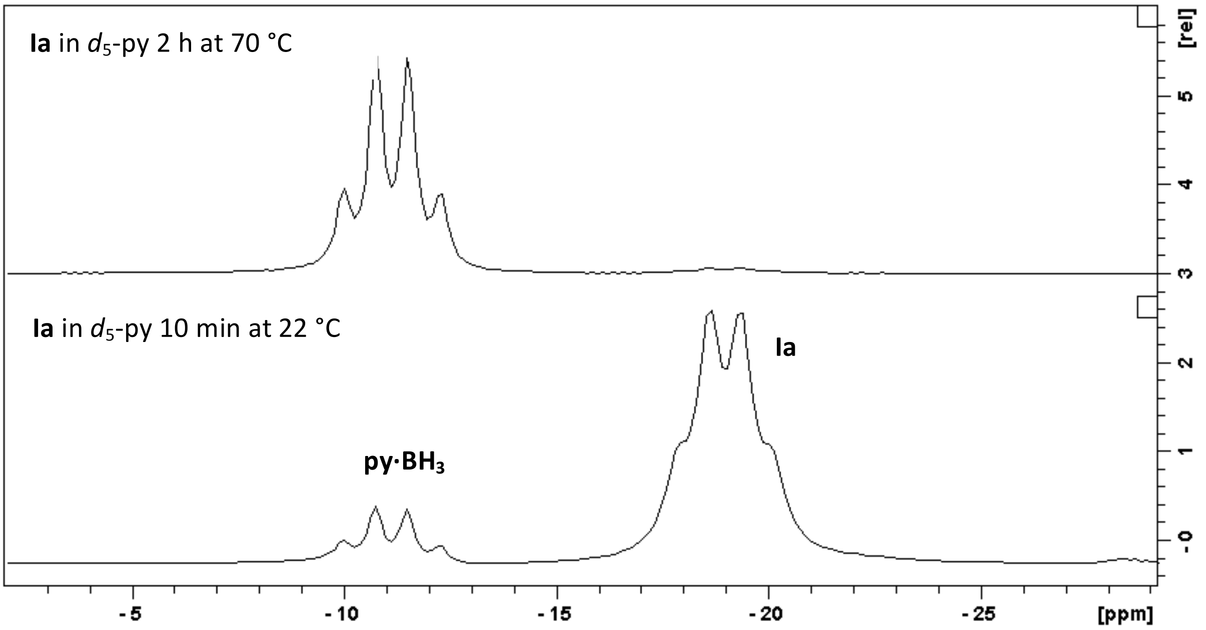

Figure S6 DOSY NMR study of major catalytic cyclisation product in d_5-pyridine after 45 min at 70 °C

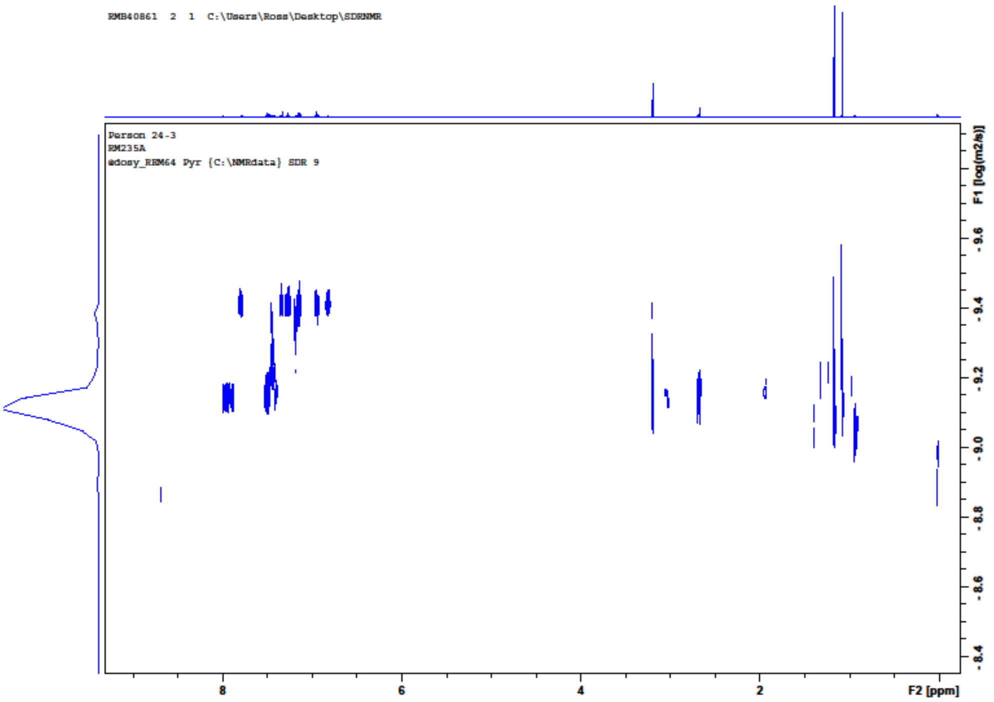

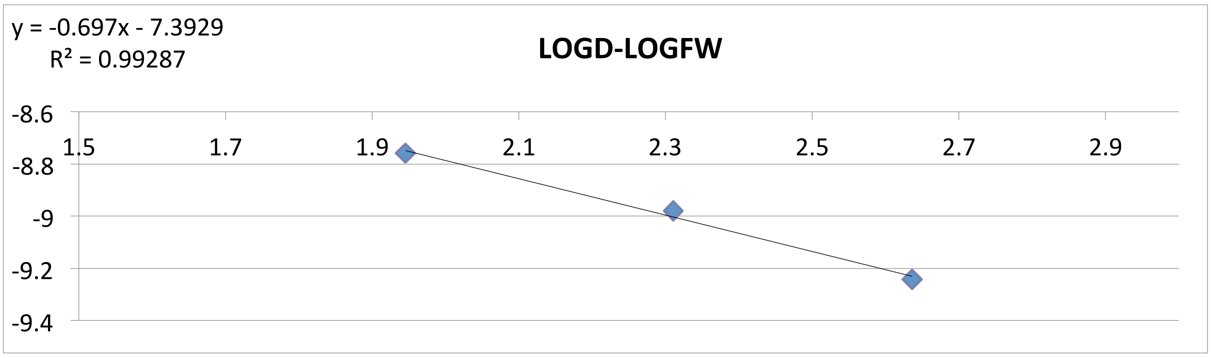

A molecular weight of 204.2 g mol^{-1} was found, higher than that of the cyclised product (182.1 g mol^{-1}, 12% error).

Figure S7 ^{1}H and ^{11}B NMR spectra of catalytic cyclisation of **1b** in d_6-benzene at 70 °C for 24 h.

^{1}H NMR Spectra

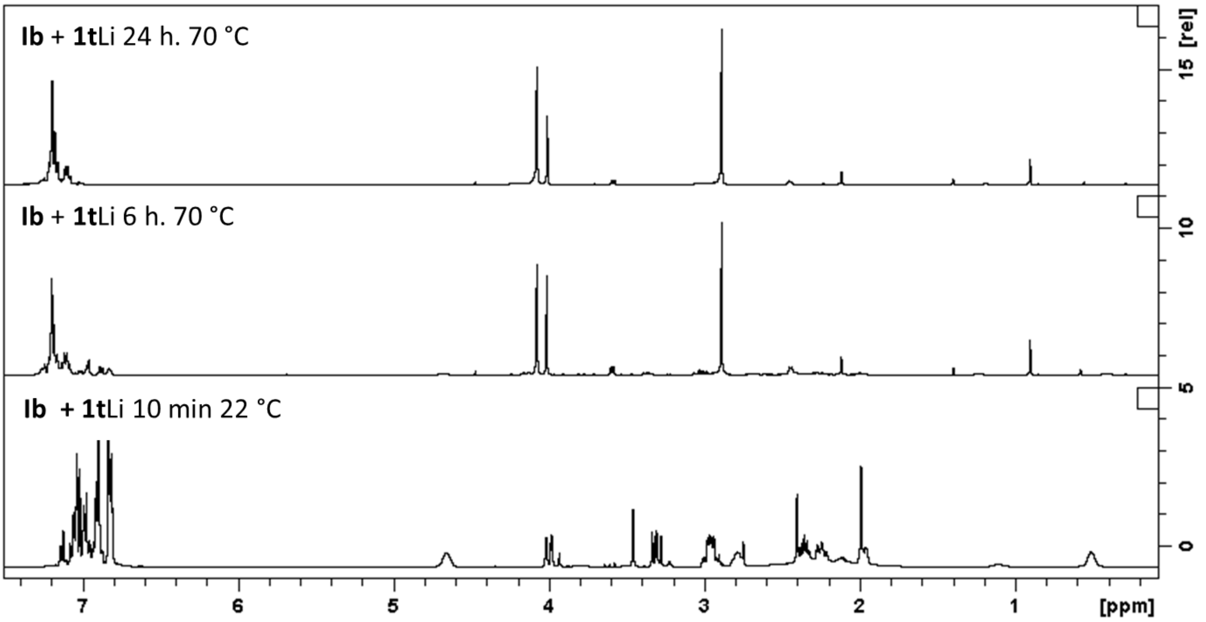

^{11}B NMR spectra

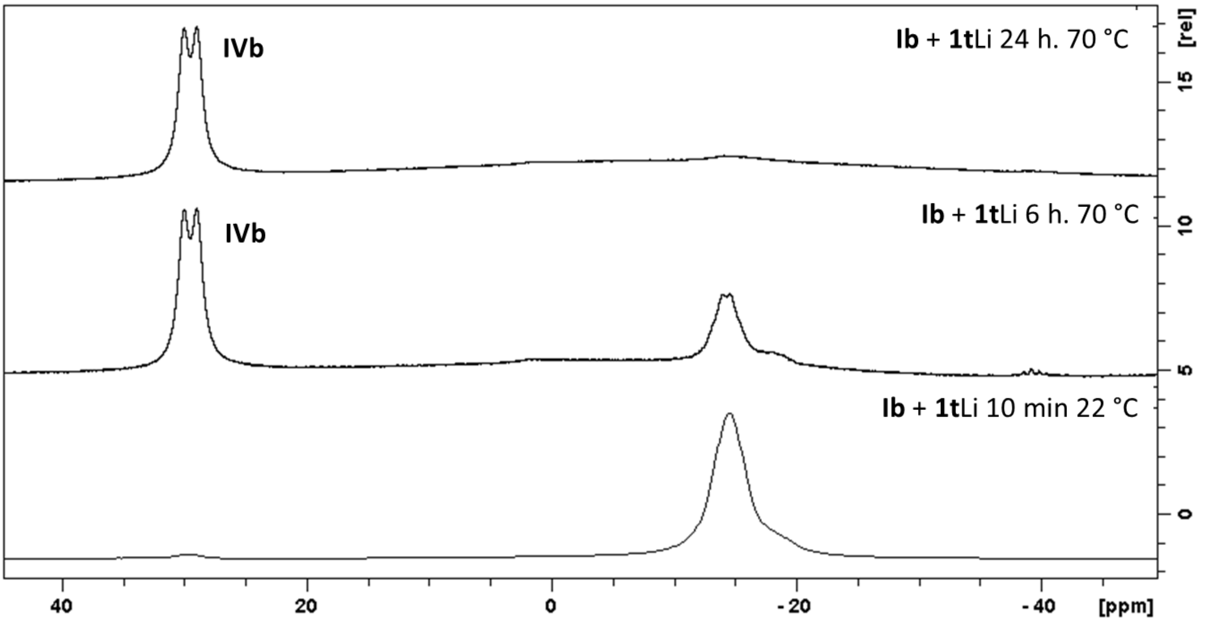

Figure S8 ^{1}H and ^{11}B NMR spectra of catalytic cyclisation of **1c** in d_6-benzene at 70 °C for 48 h.

^{1}H NMR spectra

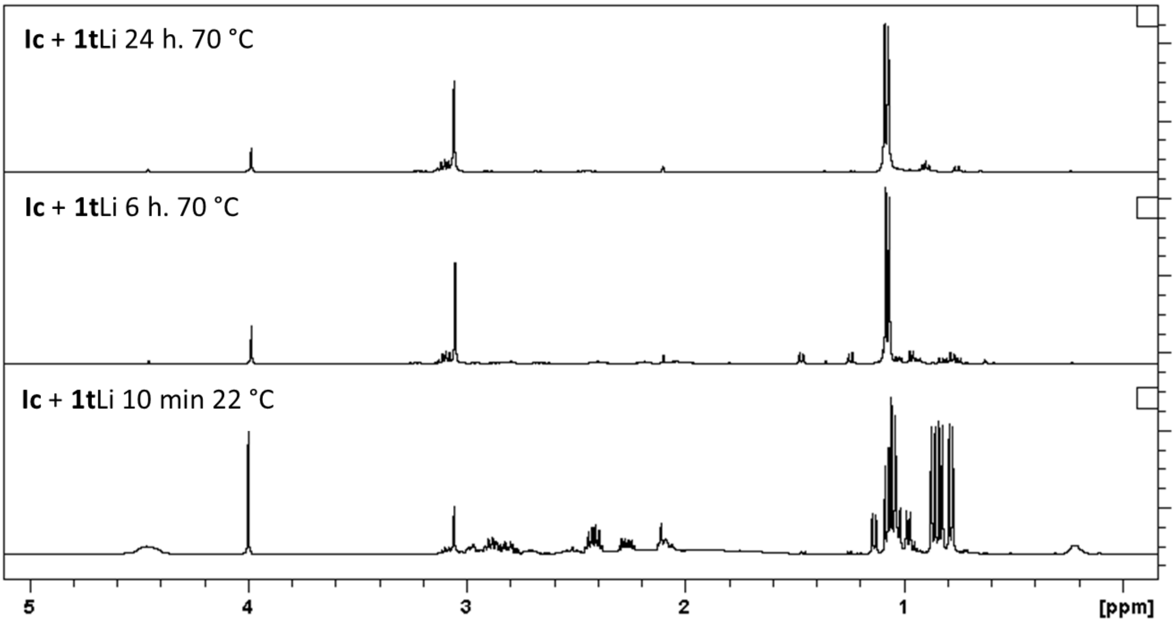

^{11}B NMR spectra

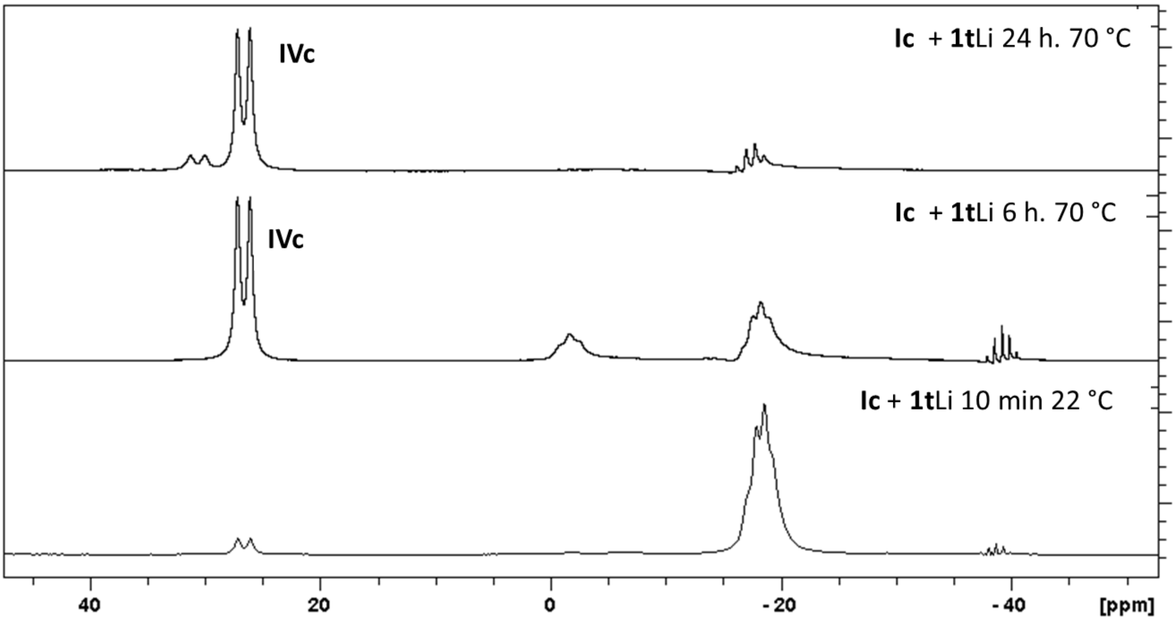

Figure S9 ^{1}H and ^{11}B NMR spectra of catalytic cyclisation of **1d** in d_6-benzene at 70 °C for 48 h.

^{1}H NMR spectra

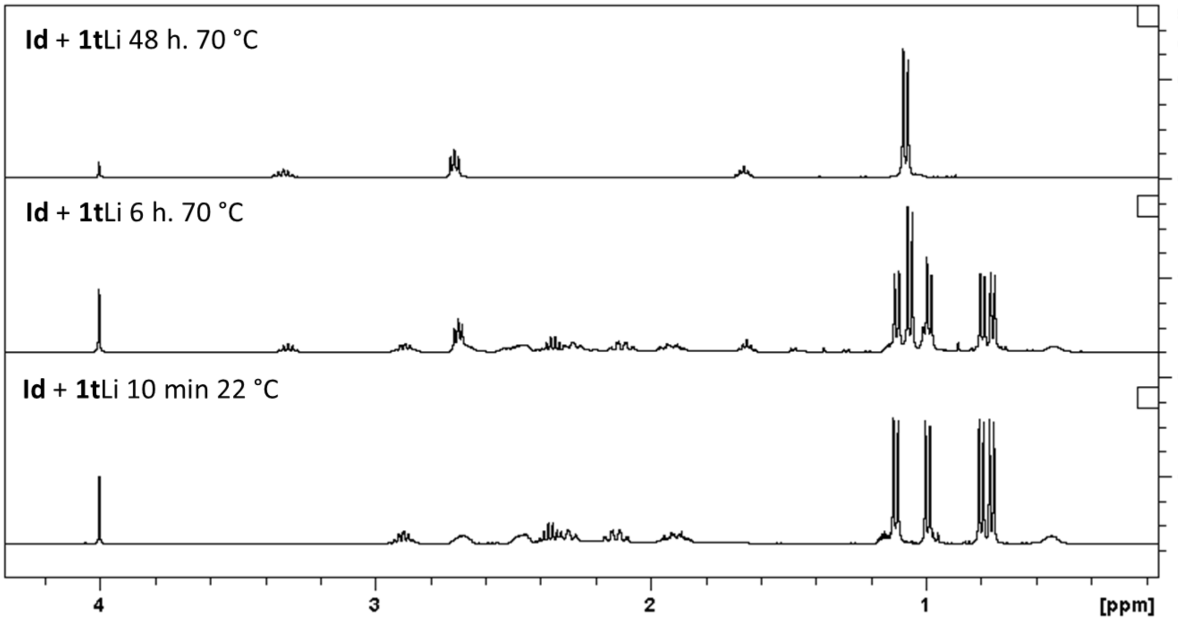

^{11}B NMR spectra

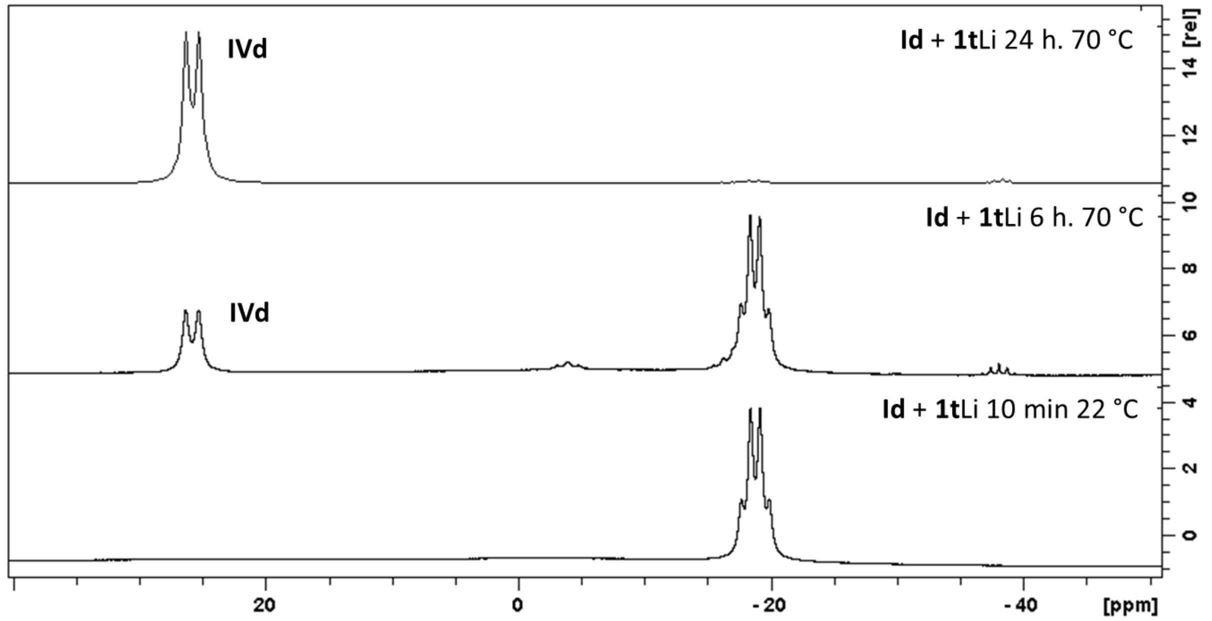

Figure S10 Identity of resonance at δ-13.4 ppm in catalytic cyclisation of **1a** in benzene.

Reaction of 2-*tert*-butylpyridine with $BH_3{\cdot}SMe_2$ in d_6-benzene shows conversion to 2-*tert*-butylpyridine·BH_3. The aromatic region in the ^{1}H NMR spectrum confims this displaying two sets of aromatic proton resonances. The ^{11}B NMR confirms that the quartet at δ-13.4 ppm in the catalytic reaction does not belong to 2-*t*-butylpyridine·BH_3

A) ^{1}H NMR spectrum 10 min at 22 °C – Aromatic region.

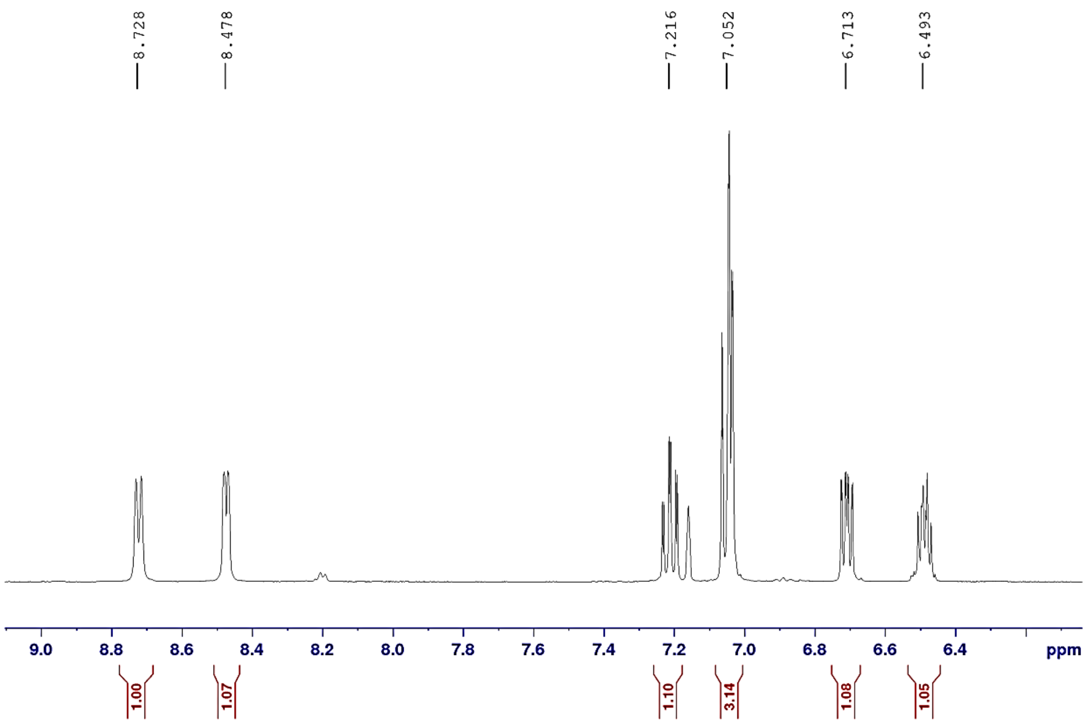

B) ^{11}B NMR spectrum 10 min at 22 °C

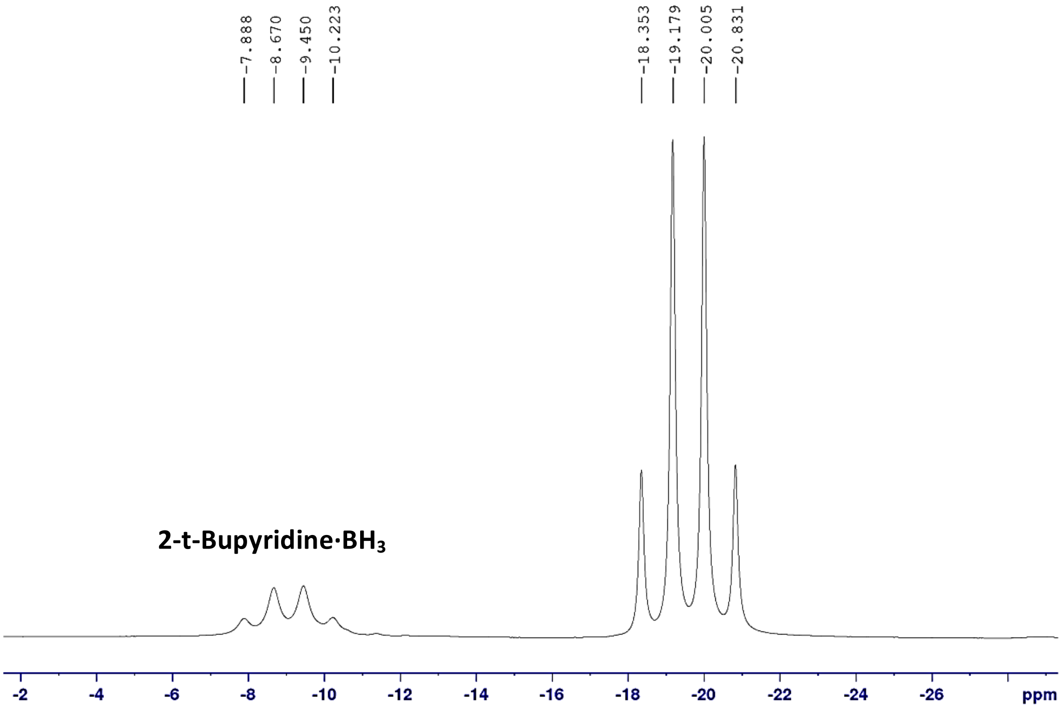

Reaction of 2-*tert*-butylpyridine with **Ia** in d_6-benzene results in formation of two products – 2-t-Bupyridine·BH_3 and an unknown BH_3 containing compound resonating at δ-13.5 ppm, in agreement with the unknown catalytic intermediate. Although the identity of this species is unknown it is clear that it only occurs in reactions involving **Ia.**

C) ^{11}B NMR spectrum 10 min at 22 °C

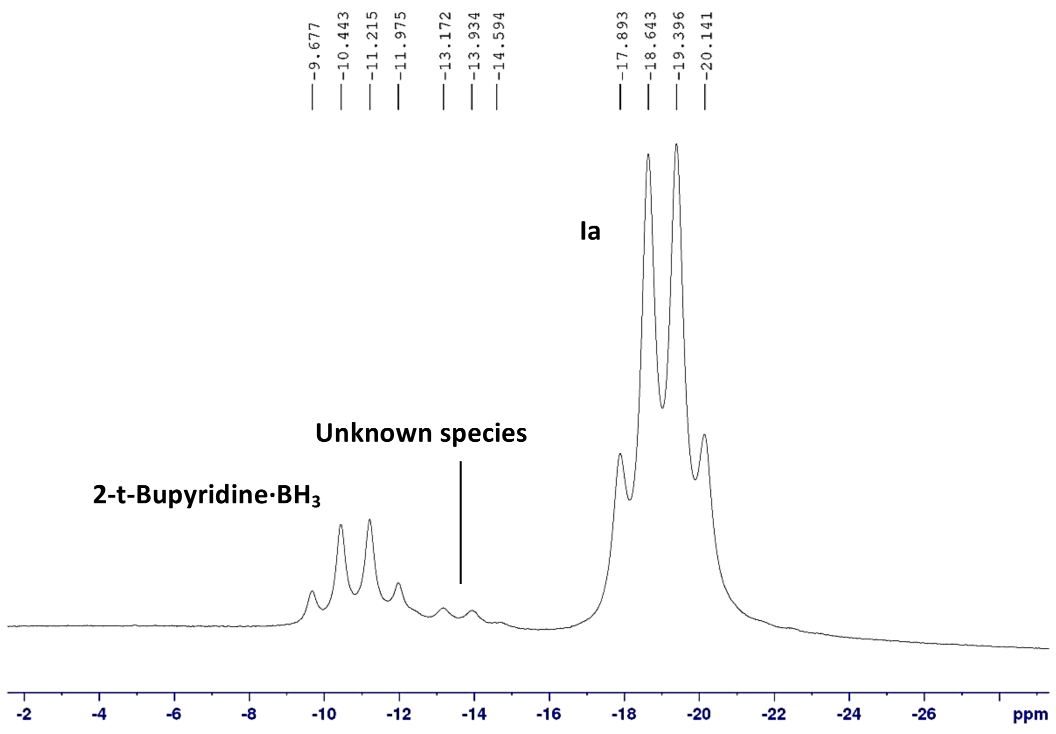

Figure S11 Control reaction between **1t**Li and THF·BH_3 in d_8-THF.

Here reaction of **1t**Li with a borane adduct containing no acidic protons results in formation of $LiBH_4$, in agreement with observations from our catalytic studies.

^{11}B NMR spectrum 16 h at 70 °C

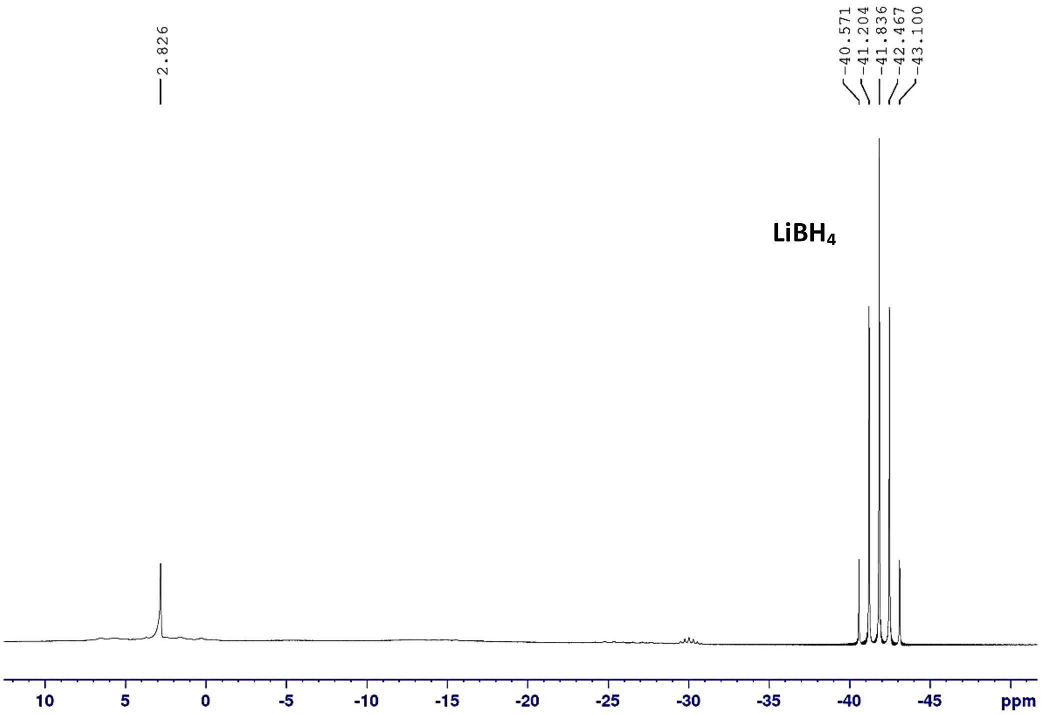

Figure S12 Catalytic dehydrogenative cyclisation of **Ia** with 5 mol% $LiBH_4$ in d_6-benzene.

^{11}B NMR spectra

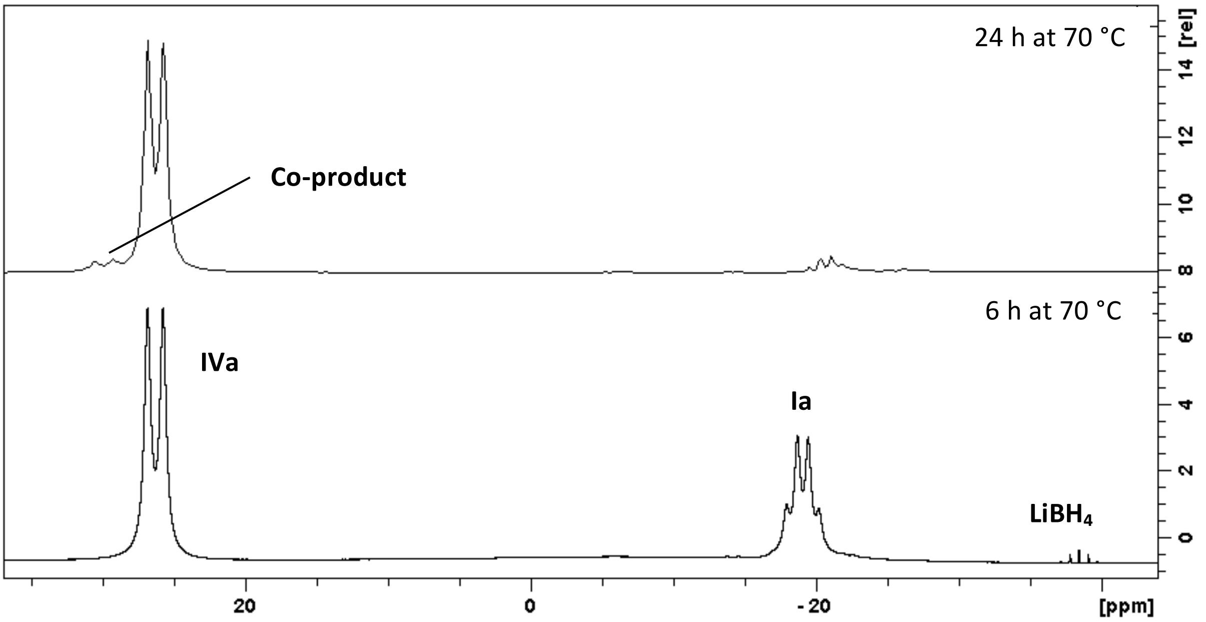

Figure S13 Structural characterisation of **IIa·THF**, **IIa·py**, **[VI·THF]$_2$** and **[VI·py]$_2$**, and selected bond parameters.

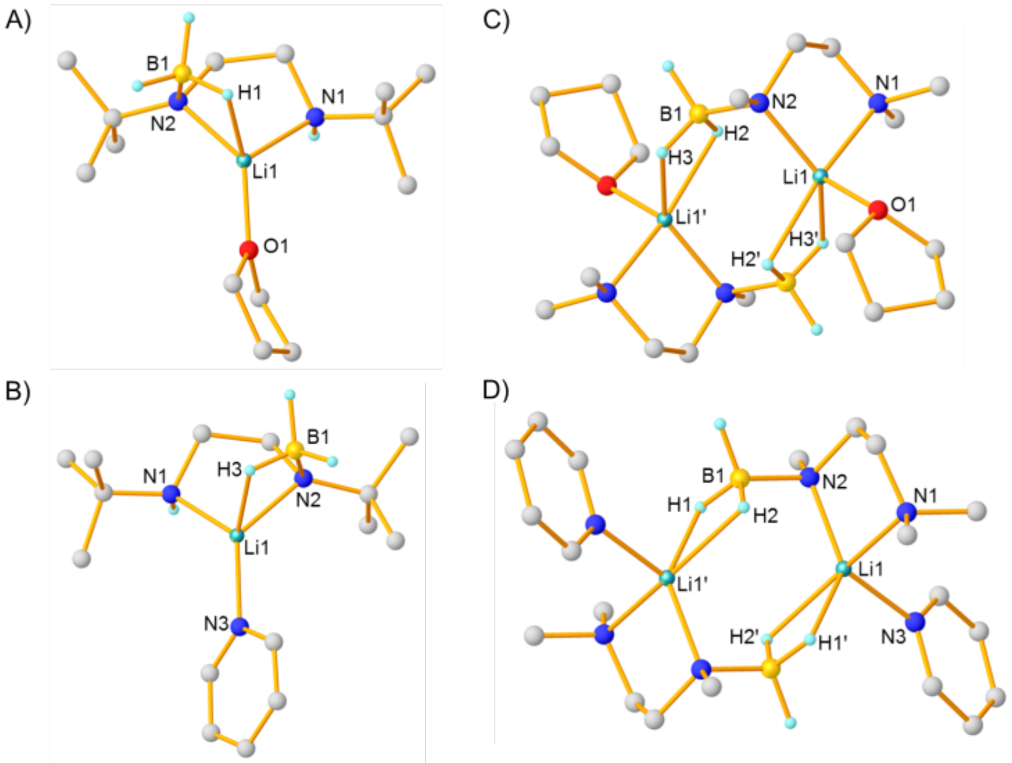

Molecular structures of A) **IIa**·THF, Selected bond lengths (Å) and angles (°): Li1-N1, 2.031(3); Li1-N2, 1.969(3); B1-N2, 1.568(2); Li1-O1, 1.907(3); Li1-H1, 1.894(18); N1-Li1-N2, 92.67(13); N1-Li1-O1, 120.15(15); N2-Li1-O1, 136.30(17); O1-Li1-H1, 125.1(6); N1-Li1-H1, 99.0(6); N2-Li1-H1, 70.0(6); B) **IIa**·py, Selected bond lengths (Å) and angles: (°): Li1-N1, 2.025(11)); Li1-N2, 1.985(12); B1-N2, 1.581(9); Li1-N3, 2.040(11); Li1-H3, 1.90(7); N1-Li1-N2, 93.4(5); N1-Li1-N3, 126.4(6); N2-Li1-N3, 131.2(6); N3-Li1-H3, 122(2); N1-Li1-H3, 99(2); N2-Li1-H3, 69(2); C) **[VI·THF]$_2$**, Selected bond lengths (Å) and angles (°): Li1-N1, 2.156(2); Li1-N2, 2.039(2); B1-N2, 1.555(1); Li1-O1, 1.984(2); H2-Li1’, 2.074(10); H3-Li1’, 1.962(13); N1-Li1-N2, 89.24(6); N1-Li1-O1, 105.92(6); N2-Li1-O1, 111.24(7); N1-Li1-H2’, 149.4(4); N1-Li1-H3’, 96.0(4); N2-Li1-H2’, 108.7(4); N2-Li1-H3’, 139.0(3); H2’-Li1-O1, 90.8(3); H3’-Li1-O1, 106.3(3). Transformations used to generate symmetry equivalent atoms -X,1-Y,-Z; and D) **[VI·py]$_2$**, Selected bond lengths (Å) and angles (°): Li1-N1, 2.163(2); Li1-N2, 2.055(2); B1-N2, 1.558(1); Li1-N3, 2.098(2); H1-Li1’, 1.960(13); H2-Li1’, 2.107(11); N1-Li1-N2, 87.79(7); N1-Li1-N3, 102.75(7); N2-Li1-N3, 117.92(8); N1-Li1-H1’, 99.4(3); N1-Li1-H2’, 152.9(4); N2-Li1-H1’, 136.7(4); N2-Li1-H2’, 105.5(4); H1’-Li1-N3, 102.1(4); H2’-Li1-N3, 91.9(4). Transformations used to generate symmetry equivalent atoms -X,1-Y,-Z. All hydrogen atoms except those attached to boron and nitrogen are omitted for clarity.

Figure S14 Stoichiometric reaction of **V** and **1t**Li in d_6-benzene

^{1}H NMR spectrum – dihydropyridyl hydrogen region

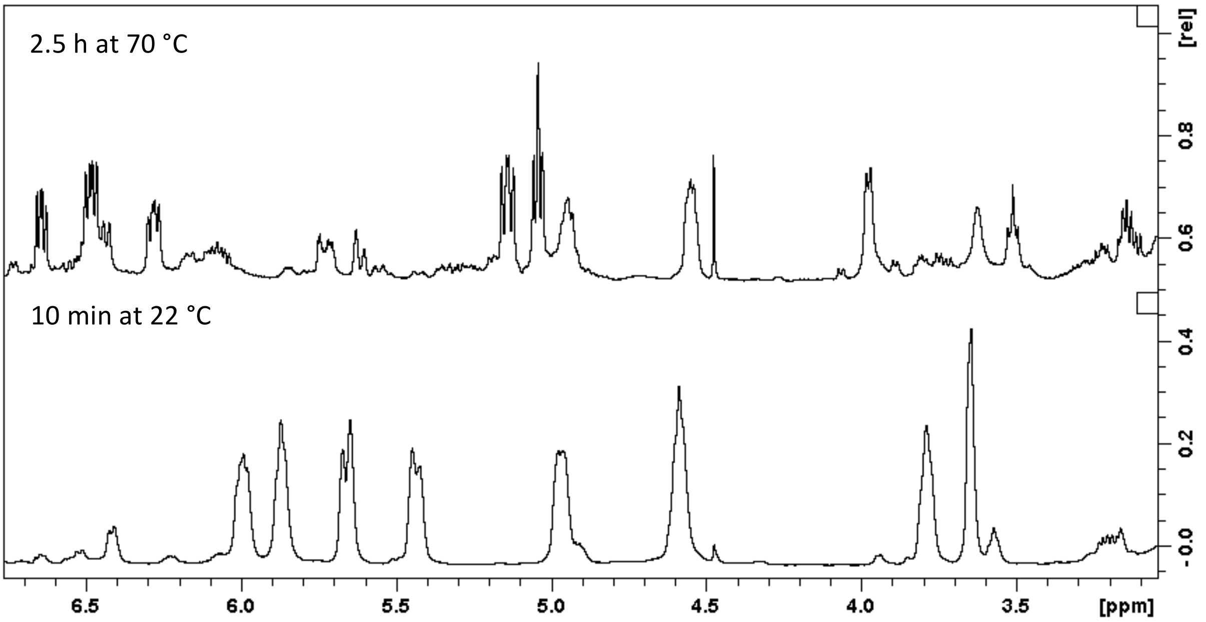

^{11}B NMR spectrum

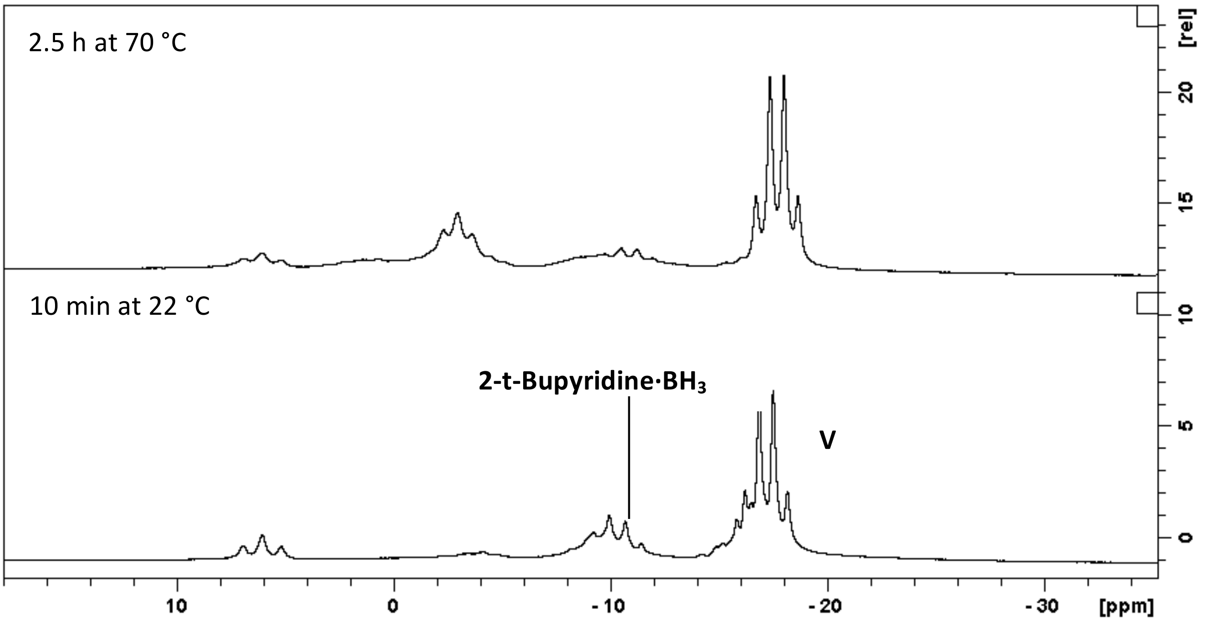

Figure S15 DOSY NMR study of **IIa·THF** in d_8-THF

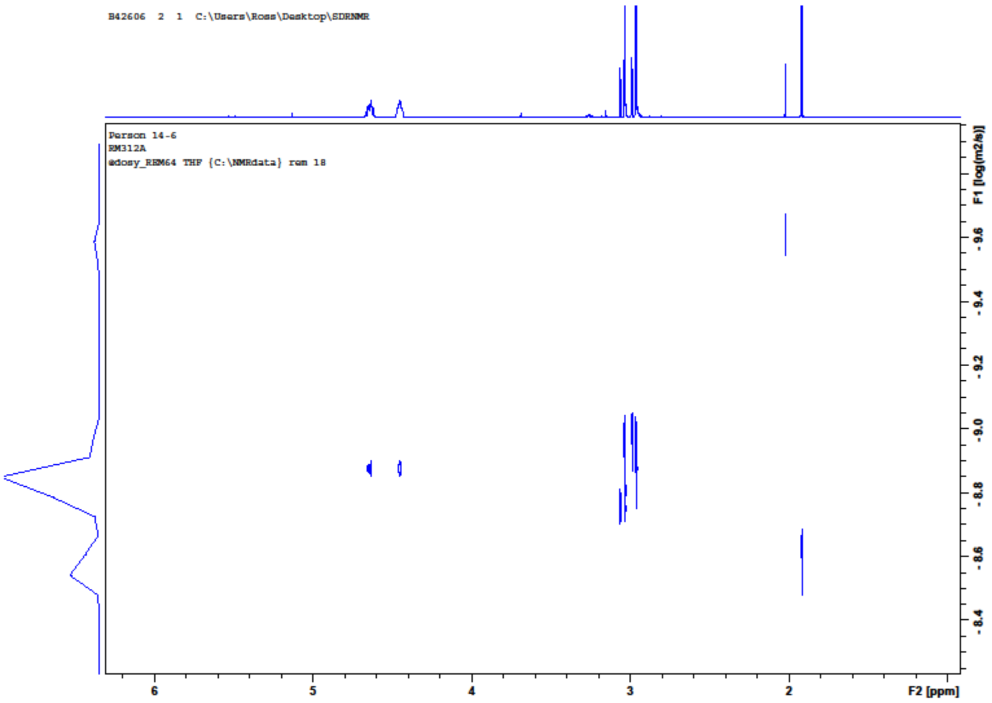

The estimated molecular weight of **IIa·THF** in d_8-THF is 297 g mol^{-1}, 12% higher than the anticipated value for a monomeric unit (264 g mol^{-1}). While this difference is reasonably large the value is still much closer to that of a monomeric unit than to a dimeric species. In addition we cannot rule out a solvation-desolvation event between a second molecule of THF and the lithium atom in **IIa·THF**. This would also lead to an increased estimated value.

ECC-MW-Determination of Small Molecules
V1.7

**Solvent:**				
**THF-d8**				
**Internal Reference**	**logDref,fix**			
TMS	-8.7018		Comment	
**Diffusion Coefficient Internal Reference [m2/s]**		**MW Analyte [g/mol]**		
2.270E-09	*Dref*	264.19		
**Diffusion Coefficient Analyte [m2/s]**		**Calibration Curve**		comment
1.208E-09	*Dx*	Dissipated Spheres + Ellipsoids (in THF)		
**normalized Diffusion Coefficient Analyte**				
-8.9758	log*Dx*,norm			
		**Predicted MW [g/mol]**	**ΔMW [%]**	
		297	-12	error ±9%

Figure S16 DOSY NMR study of **[VI·THF]**$_2$ in d_8-THF

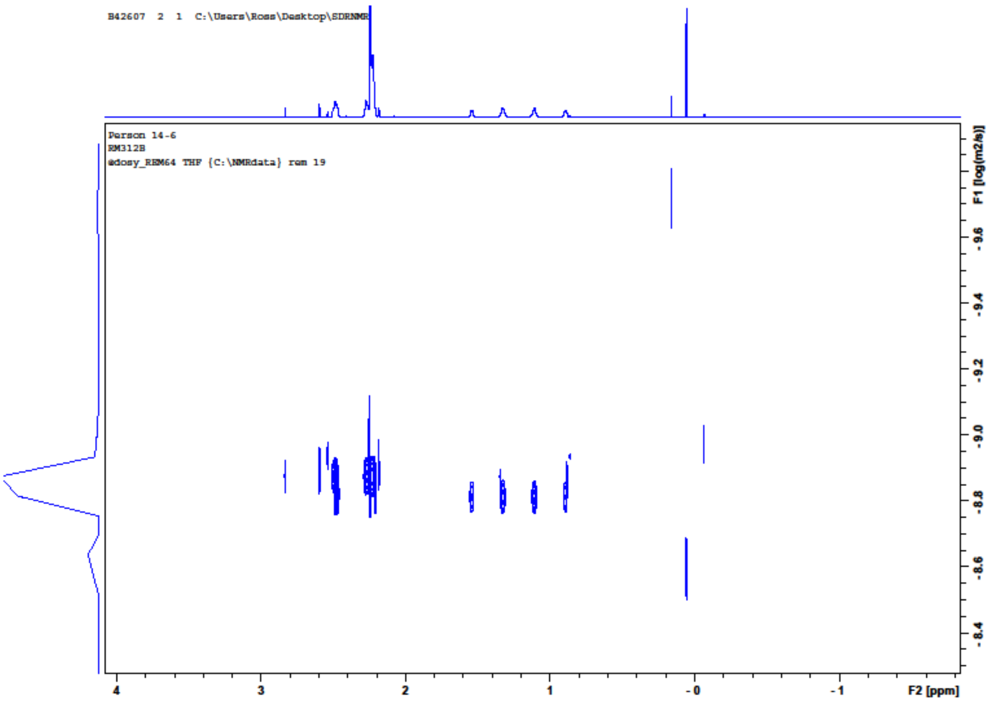

The estimated molecular weight of **[VI·THF]**$_2$ in d_8-THF is 265 g mol^{-1}, 4% higher than the anticipated value for the dimeric species observed in the crystal structure (228 g mol^{-1}, minus the solvating THF ligands from the crystal structure which were removed under application of vacuum during isolation). This small difference supports the view that a dimeric species is present in solution.

ECC-MW-Determination of Small Molecules

V1.7

Solvent:

THF-d8

**Internal Reference**	**logDref,fix**	
TMS	-8.7018	comment

**Diffusion Coefficient Internal Reference [m2/s]**		**MW Analyte [g/mol]**	
2.178E-09	*Dref*	227.77	
**Diffusion Coefficient Analyte [m2/s]**		**Calibration Curve**	comment
1.322E-09	*Dx*	Dissipated Spheres + Ellipsoids (in THF)	
**normalized Diffusion Coefficient Analyte**			
-8.9186	log*D*x,norm		

**Predicted MW [g/mol]**	**ΔMW [%]**	error
237	-4	±9%

Synthesis and NMR characterisation of IIa·THF

Ia (186 mg, 1 mmol) was dissolved in hexane (3 mL) and *n*BuLi (0.63 mL, 1 mmol 1.6M in hexane) was added, resulting in precipitation of a white solid after several minutes. After 30 min. stirring THF was added dropwise until a colourless solution was obtained. Crystals suitable for single crystal X-ray diffraction studies were grown after standing the solution at -20 °C for 24 h. Yield 193 mg, 73%.

Elemental analysis (%) calculated for $C_{14}H_{34}N_2B_1Li_1O_1$: C 63.65, H 12.97, N 10.60; found: C 63.61, H 13.01, N 10.42.

^{1}H NMR (400.1 MHz, C_6D_6 300K): δ 3.57 (4H, br t, OCH_2-THF), 2.67 (4H, br s, CH_2CH_2-diamine), 1.41 (4H, br t, (CH_2)$_2$-THF), 1.40 (9H, s, *t*Bu), 1.06 (9H, s, *t*Bu), 0.56 ppm (1H, t, $^3J_{H\text{-}H}$ 7.05 Hz, N*H*).

^{11}B NMR (128.4 MHz, C_6D_6 300K): δ -21.7 ppm (q, $^1J_{B\text{-}H}$ 86.7 Hz, BH_3).

^{7}Li NMR (155.5 MHz, C_6D_6 300K): δ 0.13 ppm.

^{13}C NMR (100.6 MHz, C_6D_6 300K): δ 67.8 (THF), 53.5 (*t*Bu quaternary), 51.6 (*C*H_2), 50.7 (*C*H_2), 42.8 (*t*Bu quaternary), 29 2 (*C*H_3-*t*Bu), 28.4 ppm (*C*H_3-*t*Bu).

Figure S17 NMR characterisation of **IIa·THF**

A) ^{1}H NMR spectrum

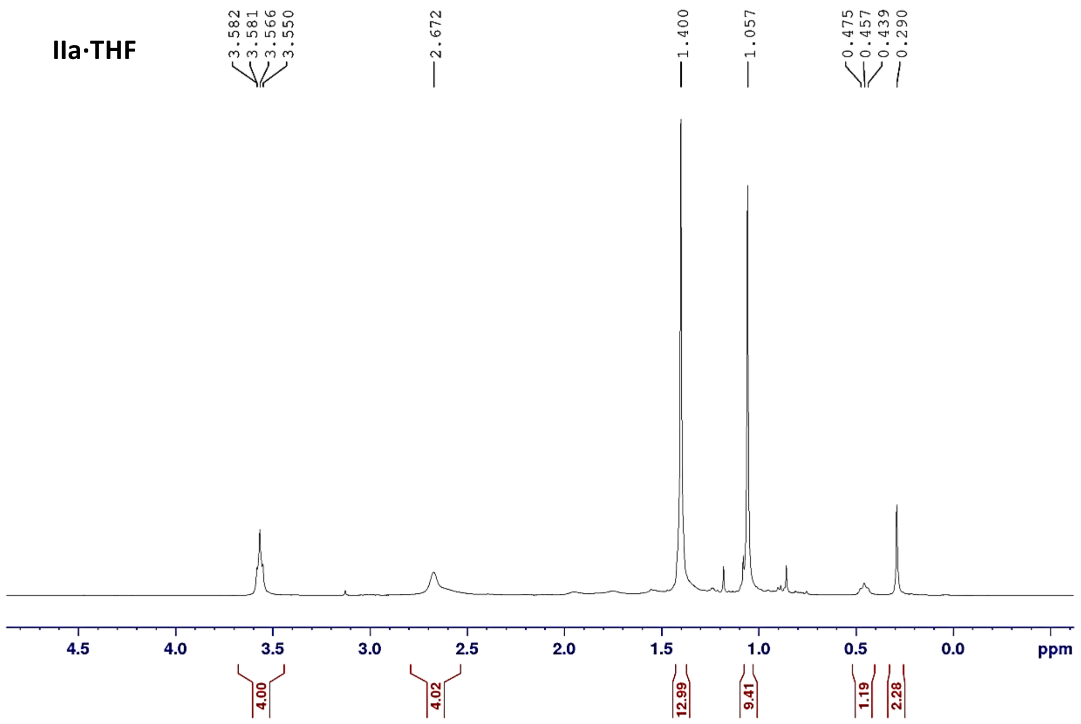

B) ^{7}Li NMR spectrum

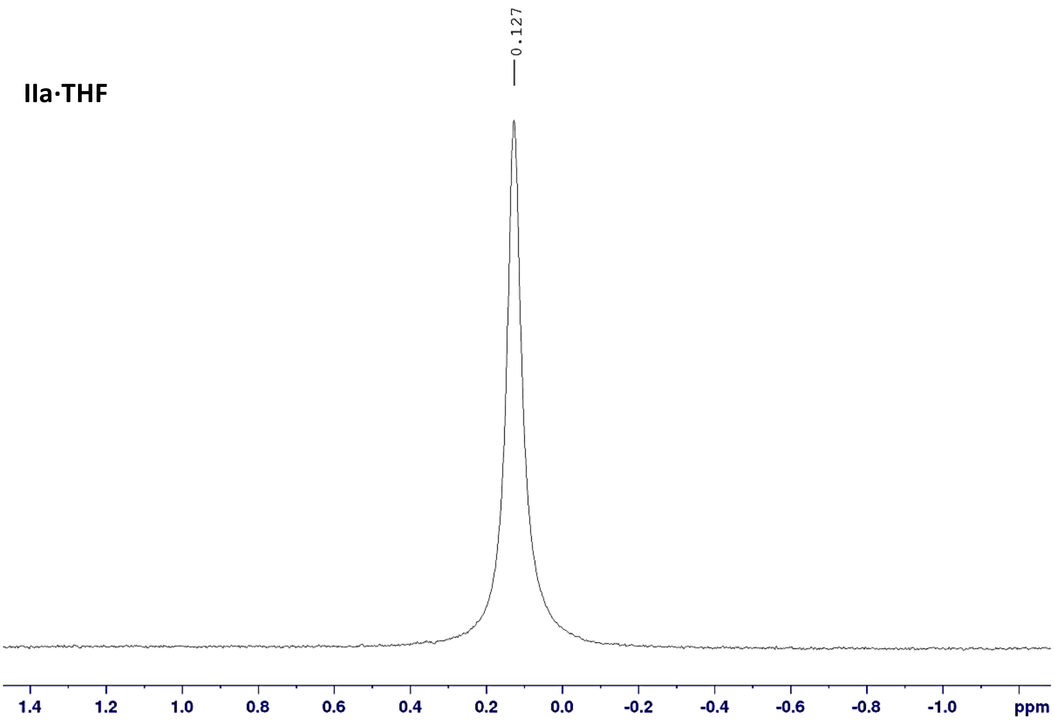

C) ^{11}B NMR spectrum

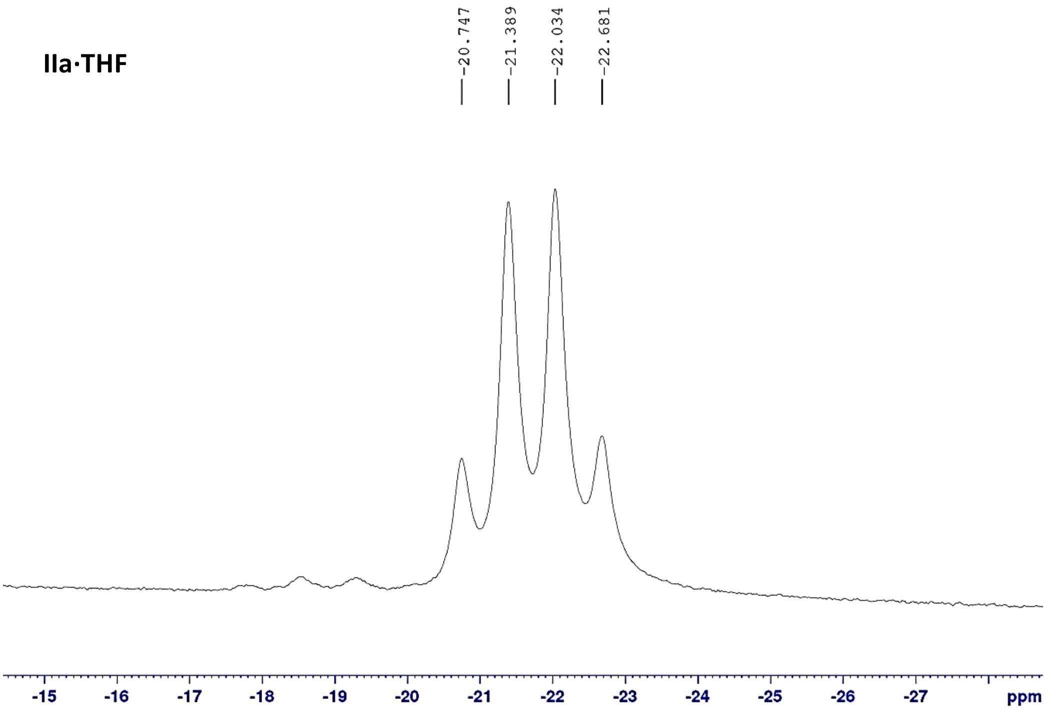

D) ^{13}C NMR spectrum

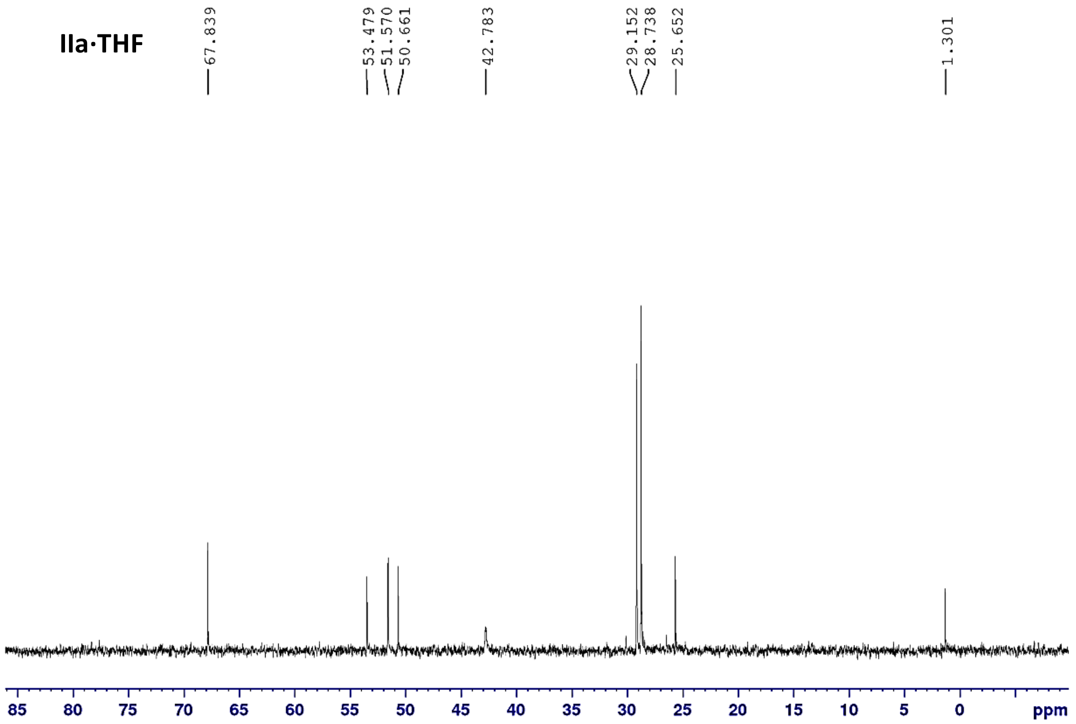

E) Infrared spectrum of **IIa·THF**

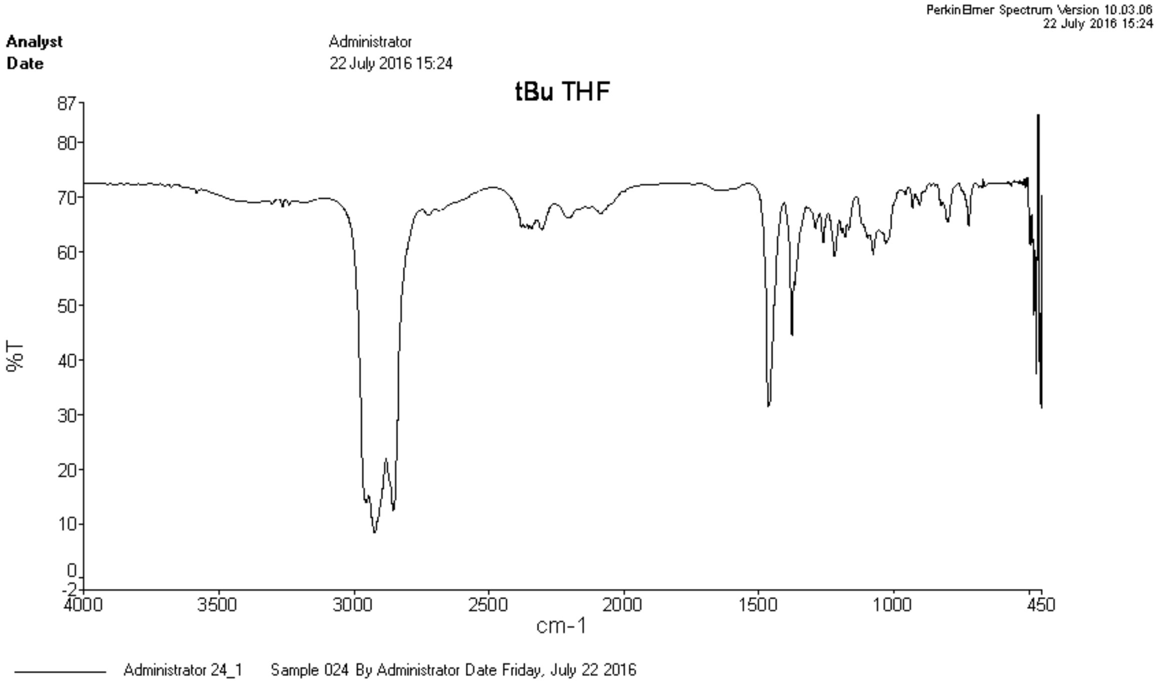

Figure S18 IIa·THF in d_6-benzene at 70 °C for 70 h.

A) ^{1}H NMR spectrum

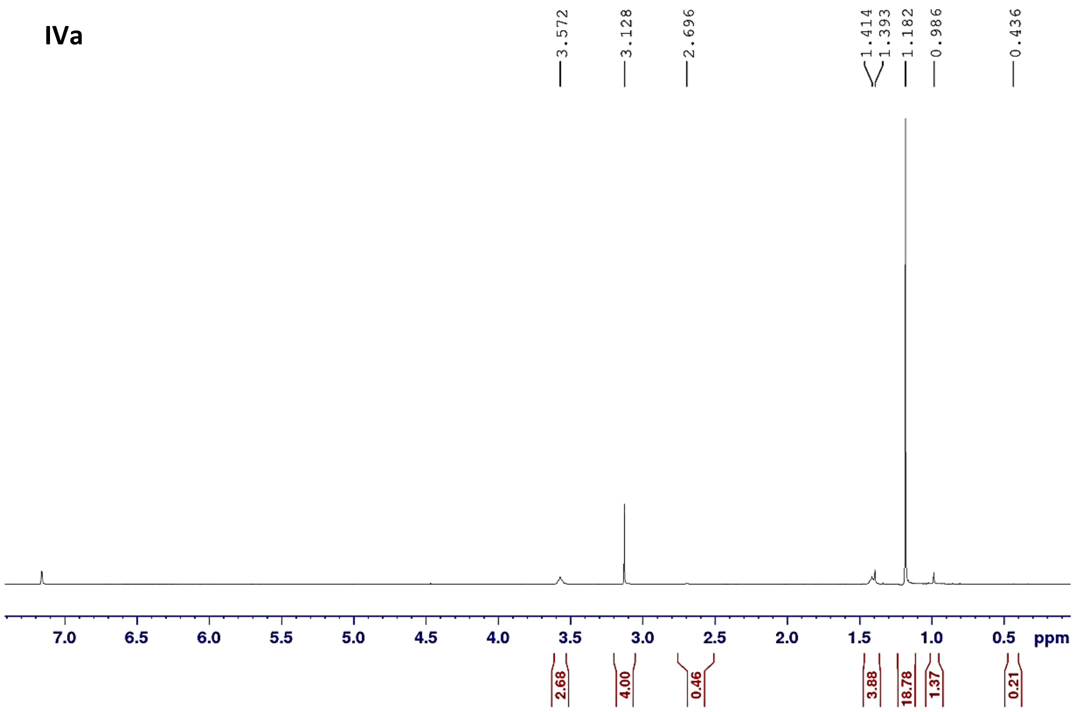

B) ^{11}B NMR spectrum

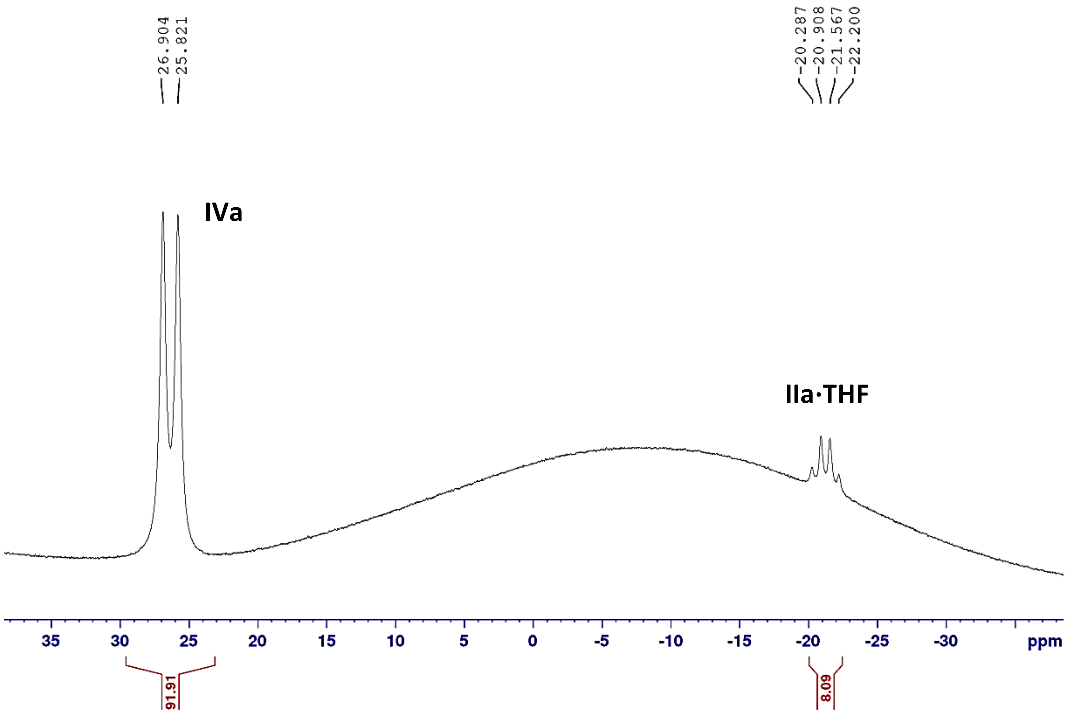

Figure S19 IIa·THF in d_5-pyridine at 70 °C for 25 h.

A) ^{1}H NMR spectrum

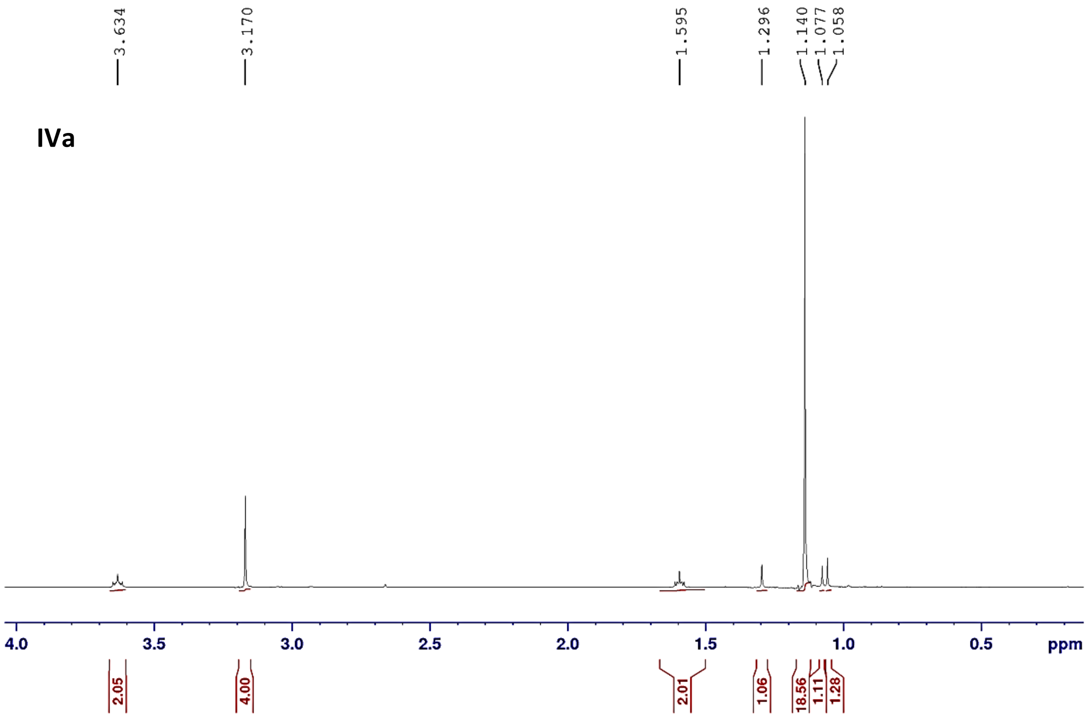

B) ^{11}B NMR spectrum

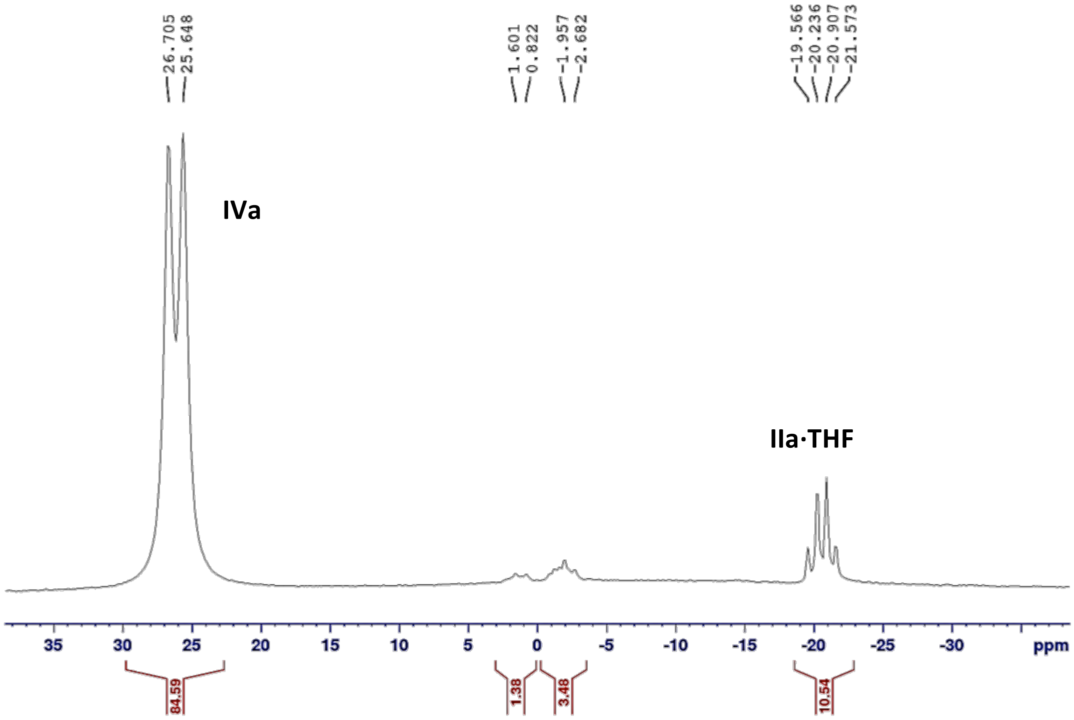

Synthesis and NMR characterisation of IIa·py

Ia (186 mg, 1 mmol) was dissolved in hexane (3 mL) and *n*BuLi (0.63 mL, 1 mmol 1.6M in hexane) was added, resulting in precipitation of a white solid after several minutes. After 30 min. stirring pyridine was added dropwise until a colourless solution was obtained. Crystals suitable for single crystal X-ray diffraction studies were grown after standing the solution at -20 °C for 24 h. Yield 169 mg, 62%.

Elemental analysis (%) calculated for $C_{15}H_{31}N_3B_1Li_1$: C 66.44, H 11.52, N 15.49; found: C 66.26, H 11.19, N 15.30.

^{1}H NMR (400.1 MHz, C_6D_6 300K): δ 8.52 (2H, m, C*H*-Pyr), 6.95 (1H, tt, $^3J_{H\text{-}H}$ 7.68 Hz; $^4J_{H\text{-}H}$ 1.93 Hz, C*H*-pyr), 6.64 (2H, m, C*H*-Pyr), 2.75 (4H, br s, C*H*$_2$C*H*$_2$-diamine), 1.41 (9H, s, *t*Bu), 1.01 (9H, s, *t*Bu), 0.54 ppm (1H, t, $^3J_{H\text{-}H}$ 8.27 Hz, N*H*).

^{11}B NMR (128.4 MHz, C_6D_6 300K): δ -21.3 ppm $^1J_{B\text{-}H}$ 84.9 Hz

^{7}Li NMR (155.5 MHz, C_6D_6 300K): δ 0.52 ppm

^{13}C NMR (100.6 MHz, C_6D_6 300K): δ 150.1 (pyr), 135.8 (pyr), 123.7 (pyr), 53.5 (*t*Bu quaternary), 51.6 (*C*H$_2$), 50.7 (*C*H$_2$), 43.0 (*t*Bu quaternary), 29 3 (*C*H$_3$-*t*Bu), 28.8 ppm (*C*H$_3$-*t*Bu).

Figure S21 NMR characterisation of **IIa·py**

A) ^{1}H NMR spectrum

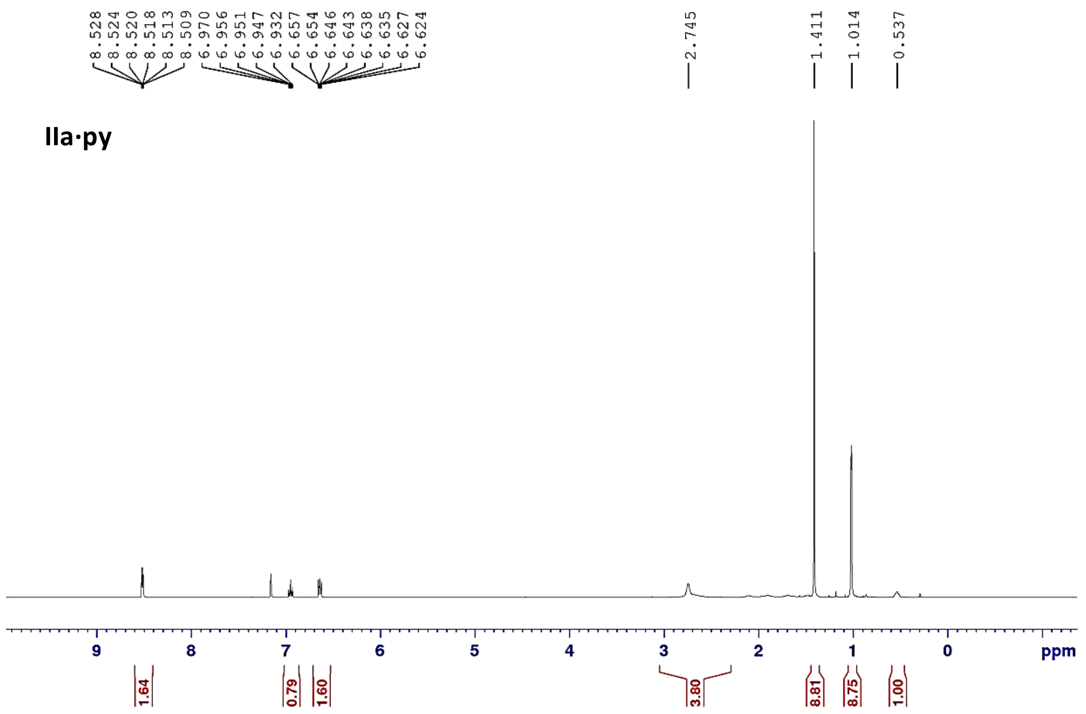

B) ^{7}Li NMR spectrum

IIa·py

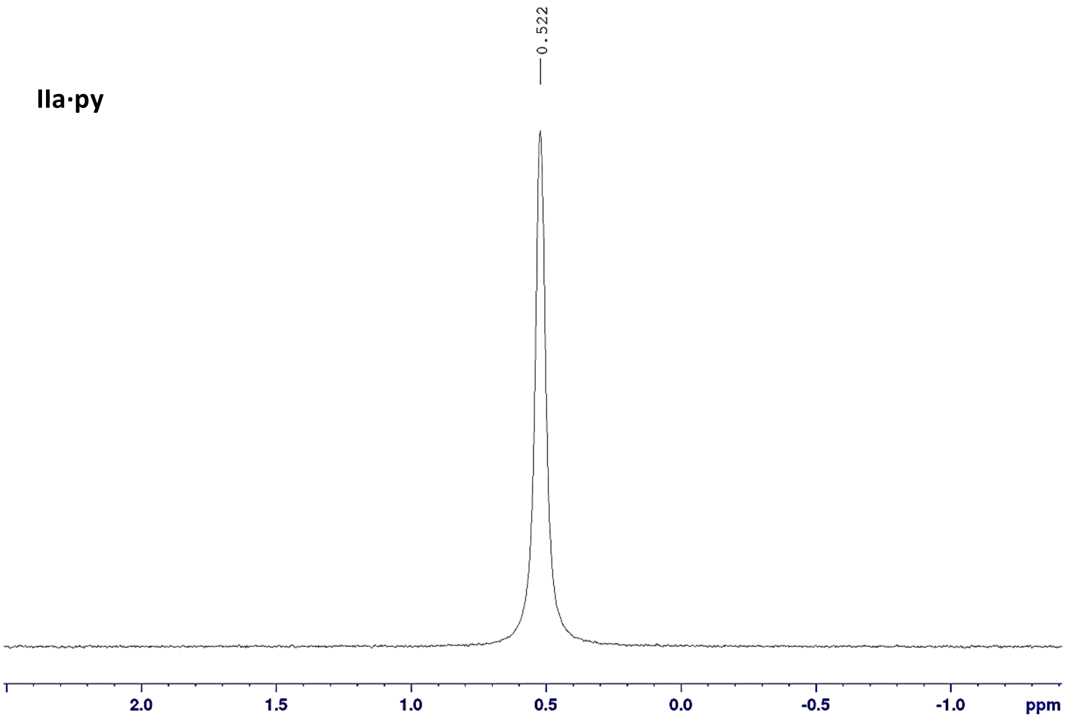

C) ^{11}B NMR spectrum

IIa·py

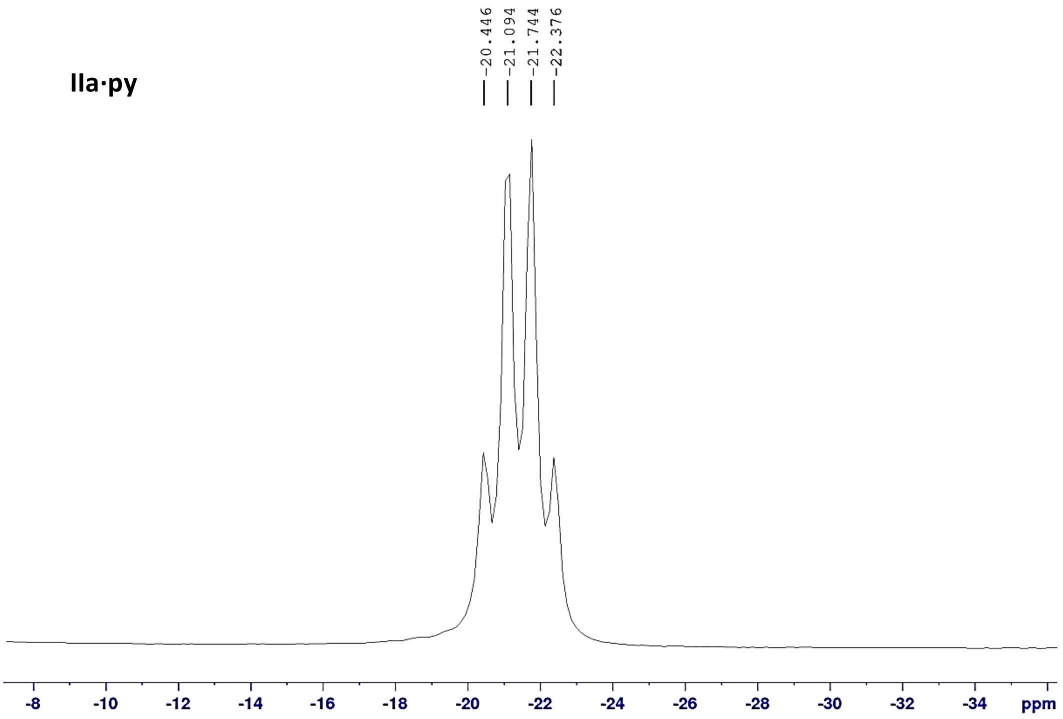

D) ^{13}C NMR spectrum

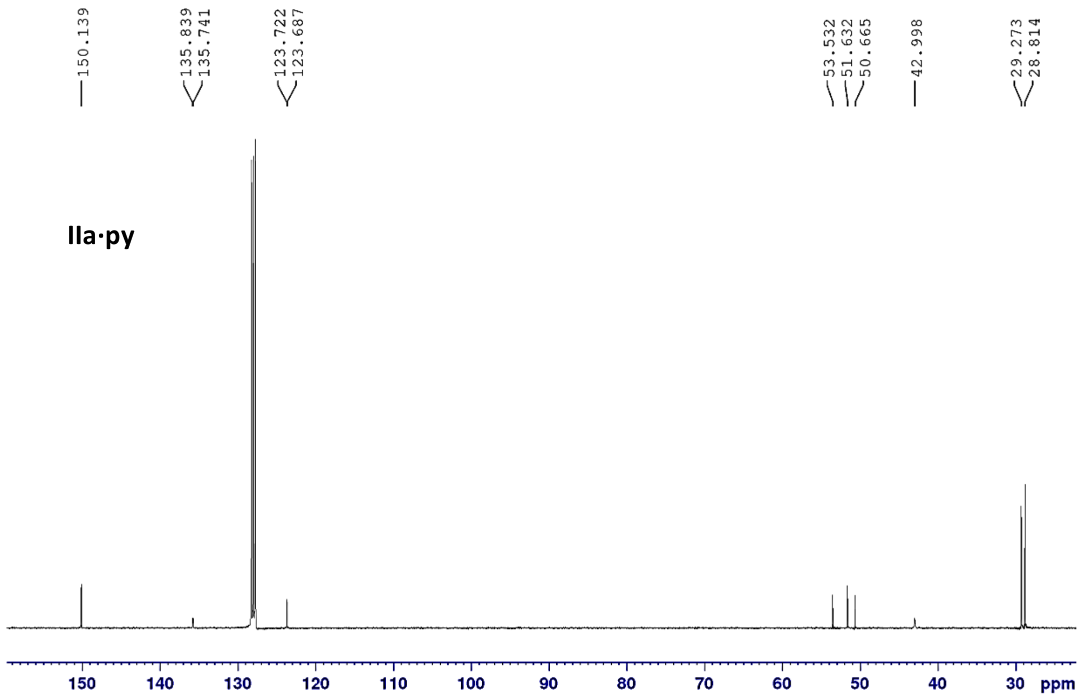

E) Infrared spectrum of **IIa·py**

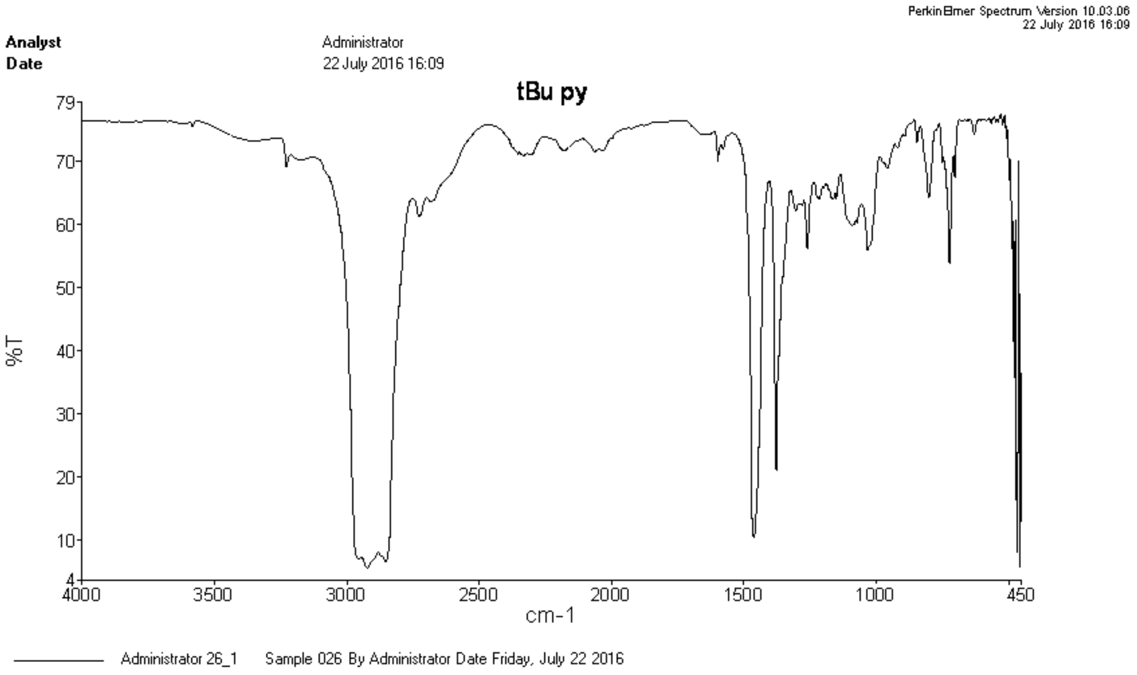

Figure S22 IIa·py in d_6-benzene at 70 °C for 22 h.

A) ^{1}H NMR spectrum

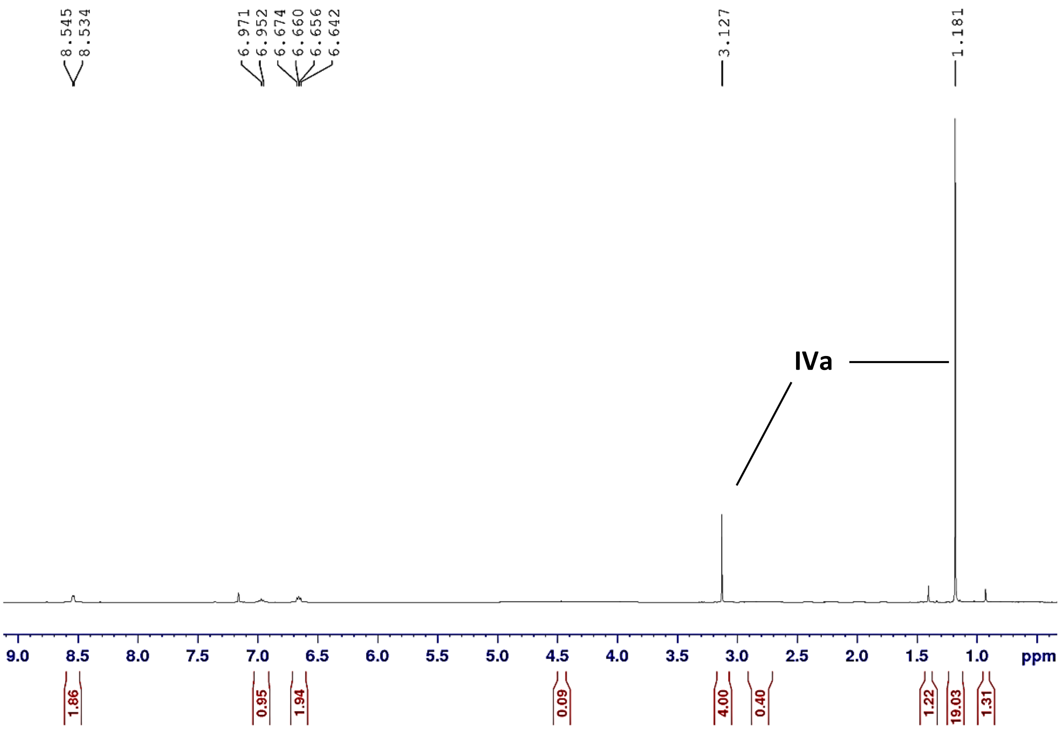

B) Expansion of ^{1}H NMR showing resonances attributable to DHP species.

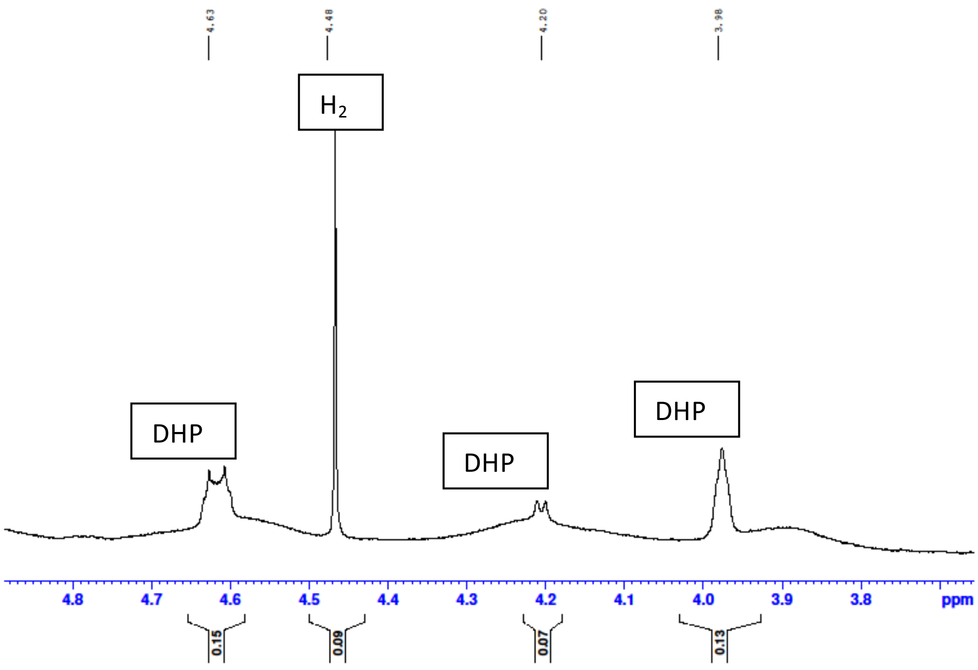

C) ^{11}B NMR spectrum

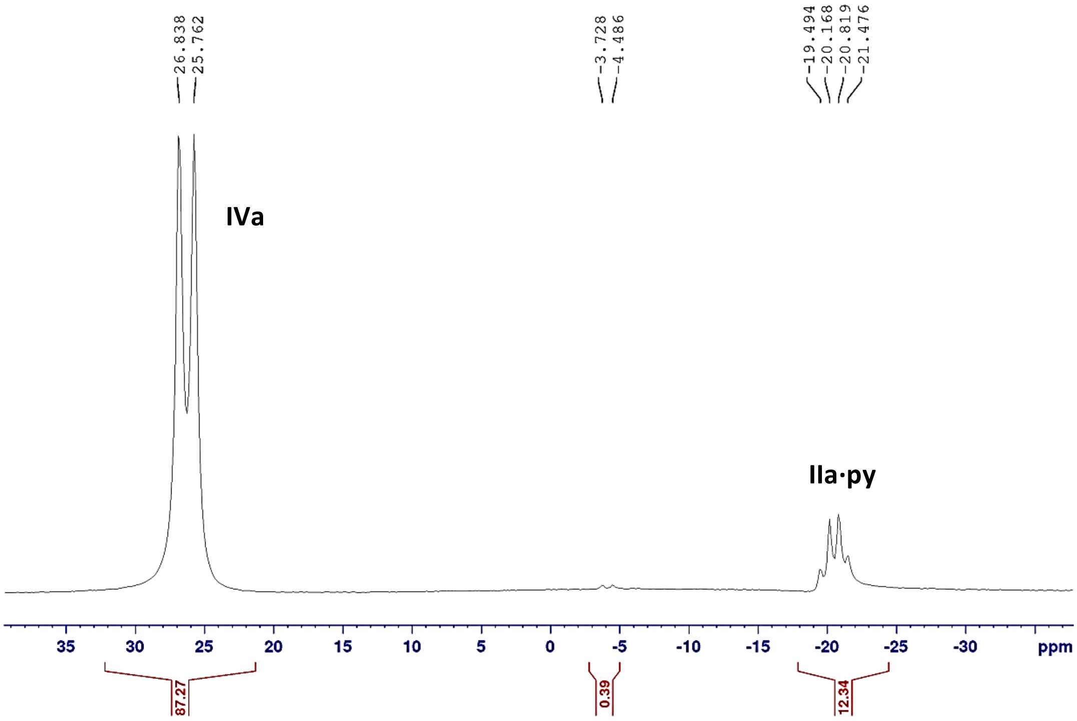

Synthesis and NMR characterisation of [VI·THF]$_2$

V (116 mg, 1 mmol) was dissolved in hexane (3 mL) and *n*BuLi (0.63 mL, 1 mmol 1.6M in hexane) was added, resulting in precipitation of a white solid after several minutes. After 30 min. stirring THF was added dropwise until a colourless solution was obtained. Crystals suitable for single crystal X-ray diffraction studies were grown after standing the solution at -20 °C for 24 h. Yield 112 mg, 81%.

Elemental analysis (%) calculated for $C_{10}H_{32}N_4B_2Li_2$: C 49.25, H 13.23, N 22.97; found: C 49.51, H 12.28, N 22.92. Consistent with loss of 2 x THF upon drying *in vacuo*.

^{1}H NMR (400.1 MHz, C_6D_6 300K): δ 2.64 (3H, br s, CH_3-diamine), 2.45 (2H, br s,CH_2-diamine), 2.34 (2H, br s,CH_2-diamine), 2.01 ppm (1H, s, 2xCH_3-diamine).

^{11}B NMR (128.4 MHz, C_6D_6 300K): δ -17.9 ppm $^1J_{B\text{-}H}$ 83.3 Hz

^{7}Li NMR (155.5 MHz, C_6D_6 300K): δ 0.56 ppm

^{13}C NMR (100.6 MHz, C_6D_6 300K): δ 68.0 (THF), 58.6 (CH$_2$), 57.1 (CH$_2$), 48.0 (CH$_3$), 45.0 (2xCH$_3$), 25.6 ppm (THF).

Figure S23 NMR characterisation of **[VI·THF]$_2$**

A) ^{1}H NMR spectrum

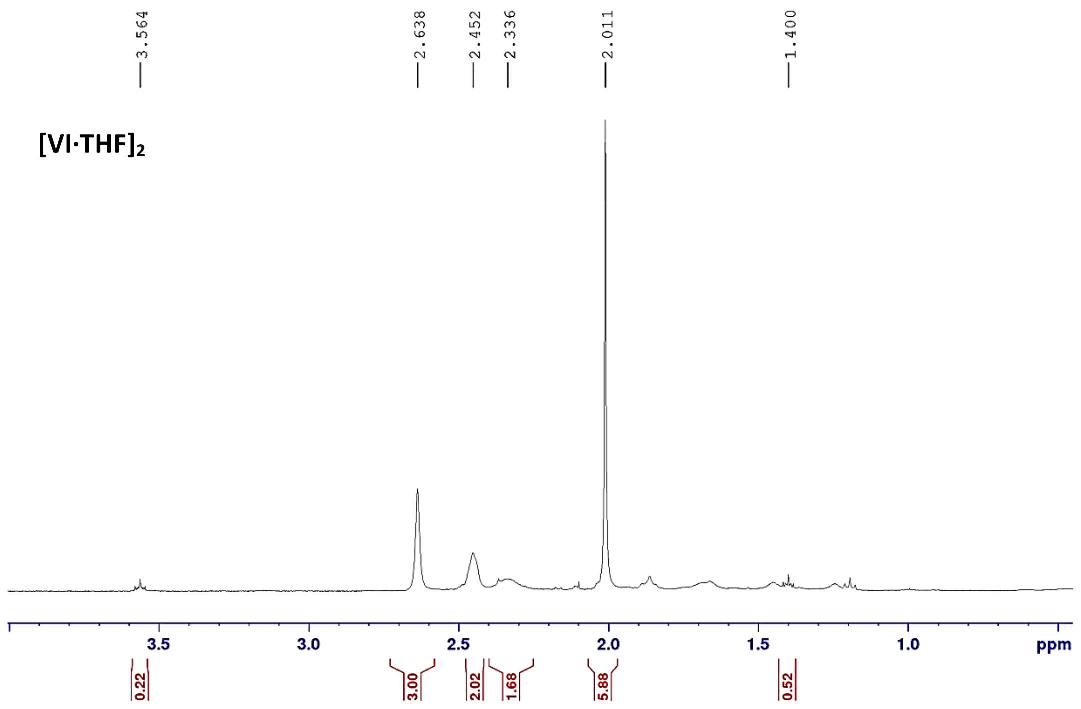

B) ^{7}Li NMR spectrum

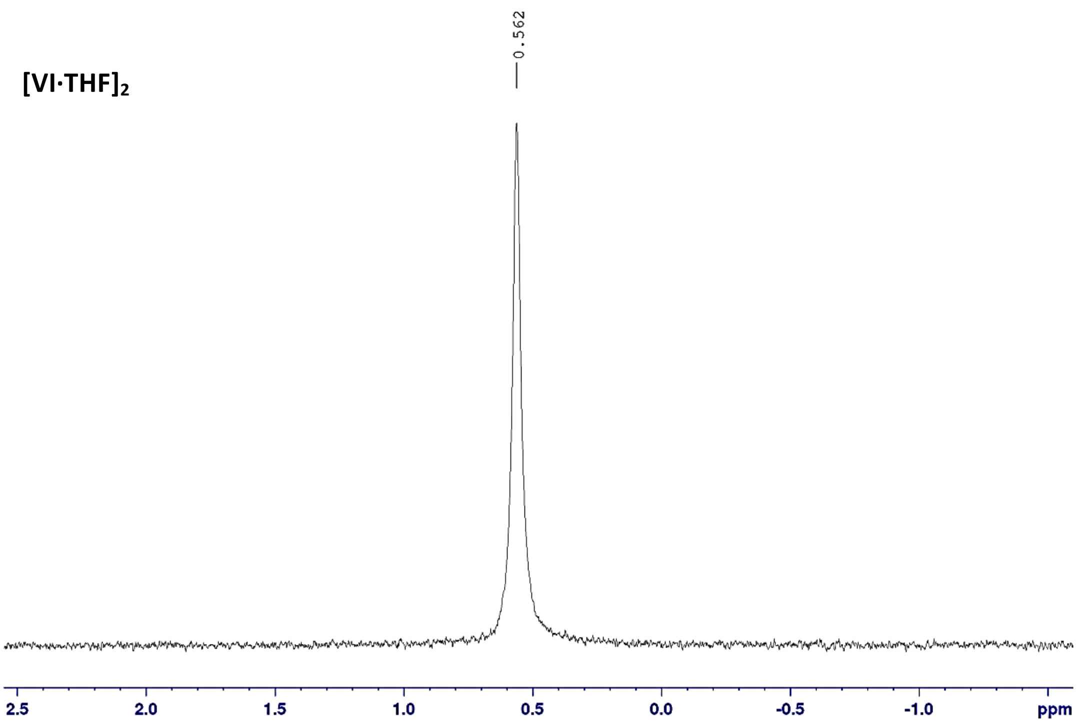

C) ^{11}B NMR spectrum

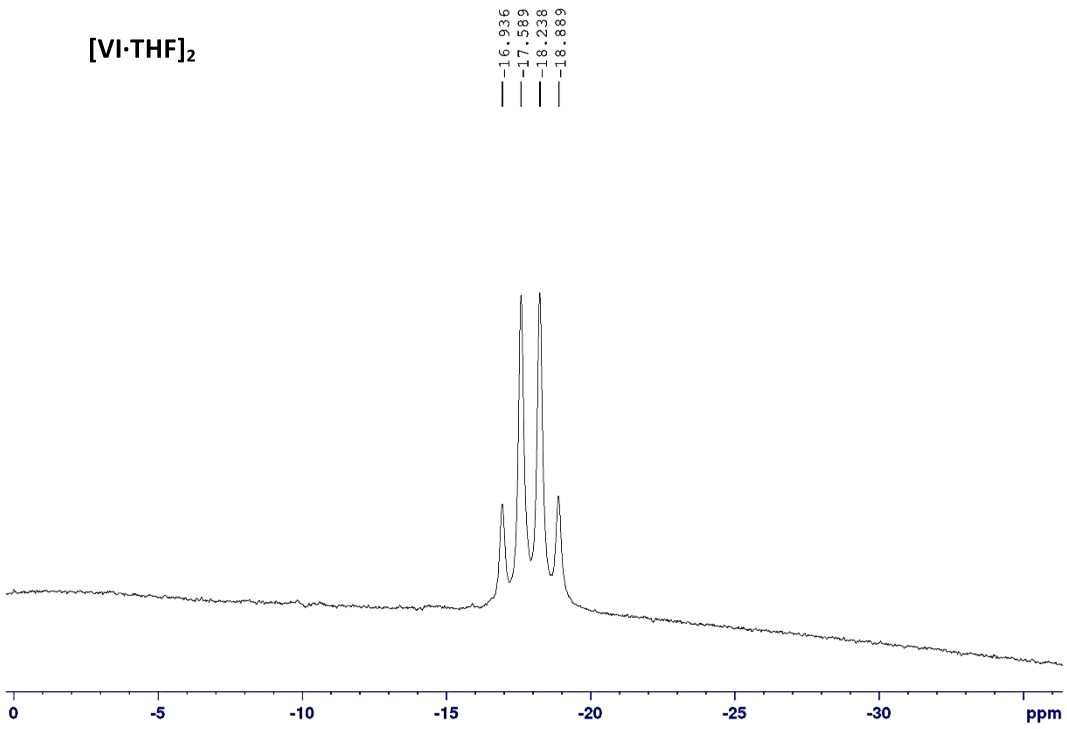

D) ^{13}C NMR spectrum

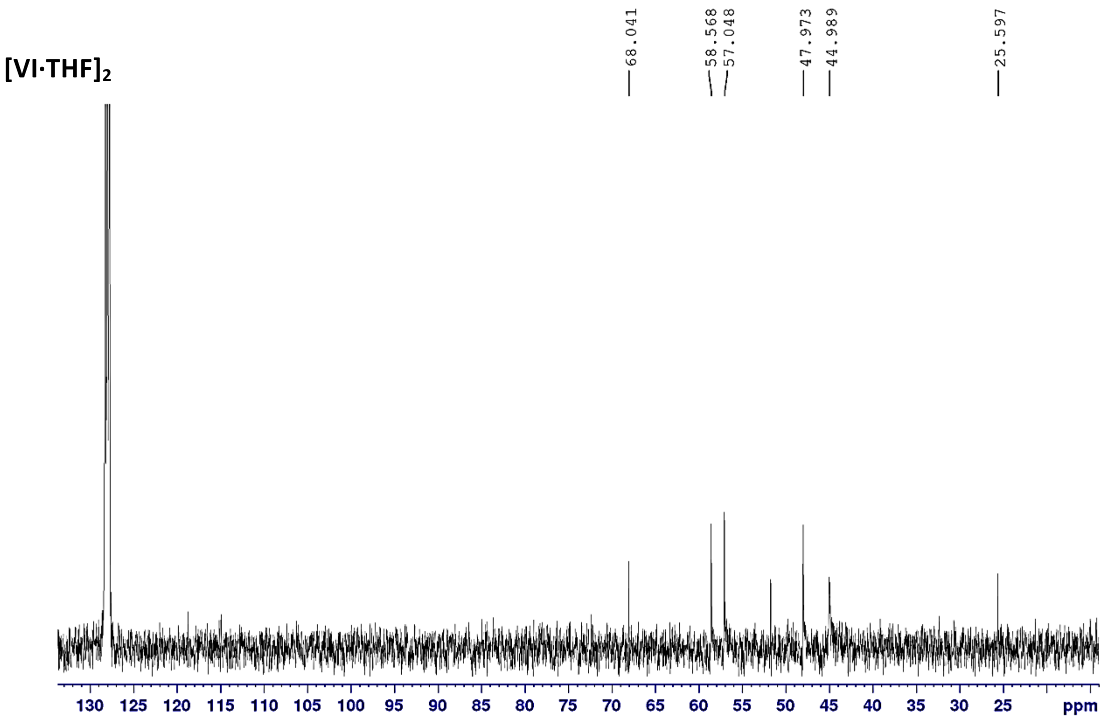

E) Infrared spectrum of **[VI·THF]$_2$**

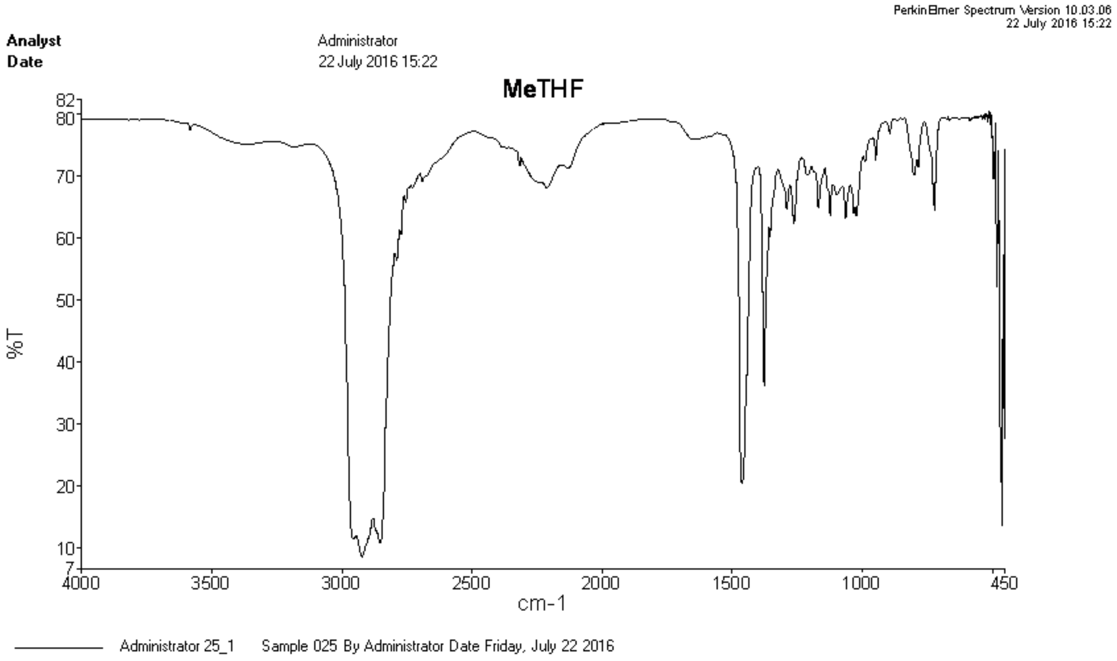

Figure S24 [VI·THF]$_2$ in d_6-benzene at 70 °C for 90 h.

A) ^{1}H NMR spectrum

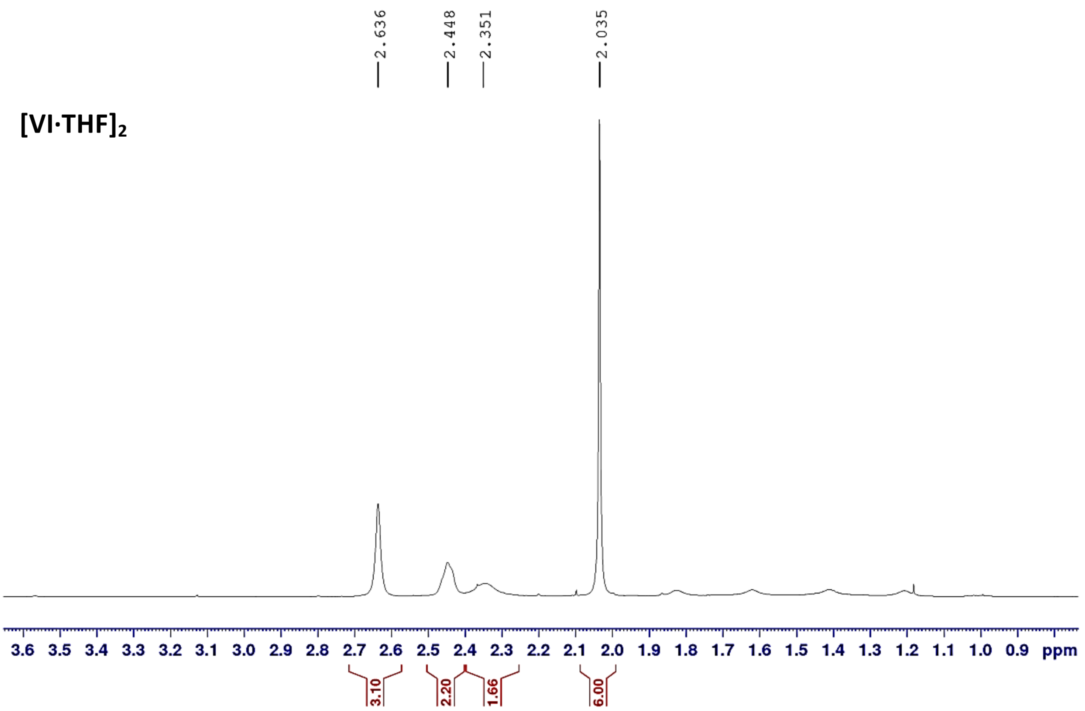

B) ^{11}B NMR spectrum

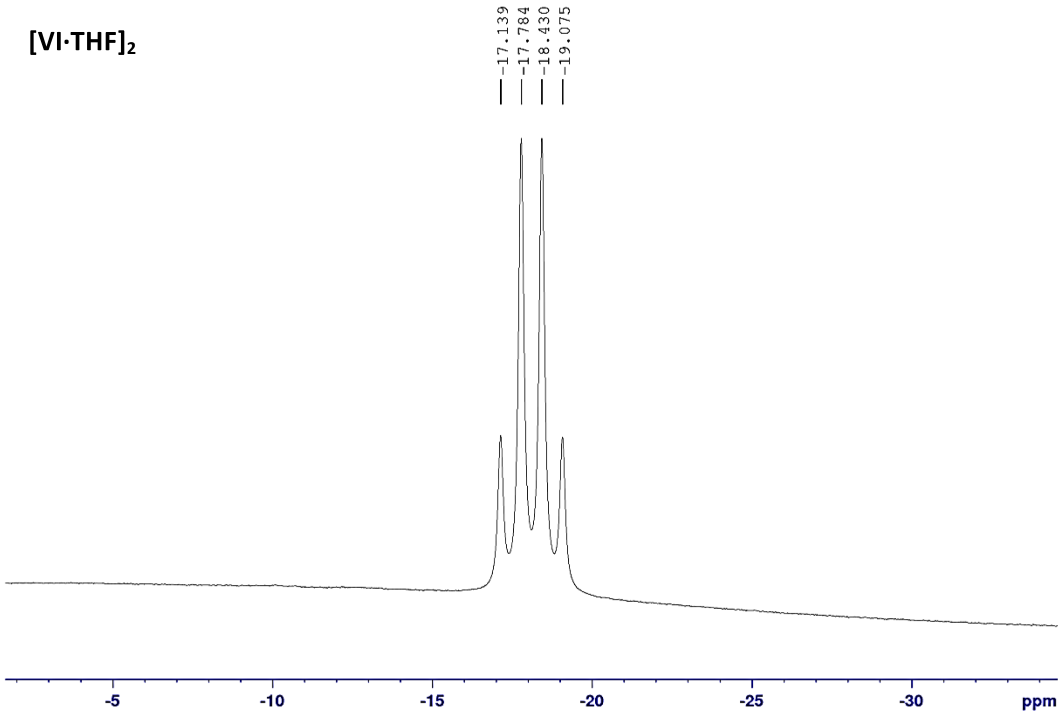

Figure S25 [VI·THF]$_2$ in d_5-pyridine at 70 °C for 90 h.

A) ^{1}H NMR spectrum

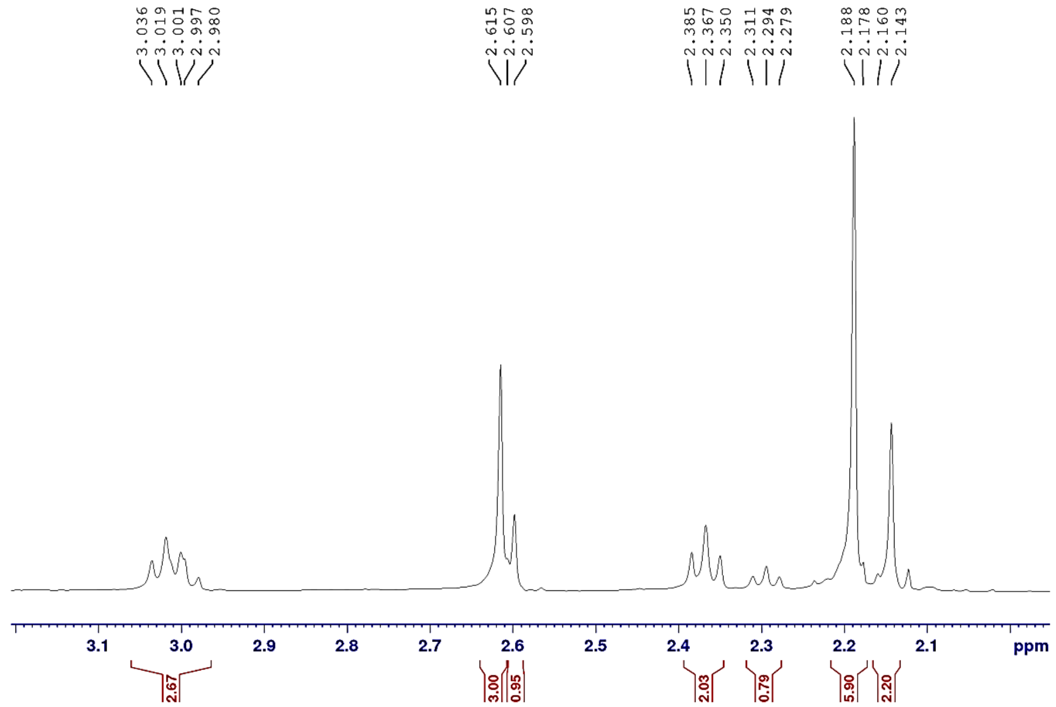

B) ^{11}B NMR spectrum

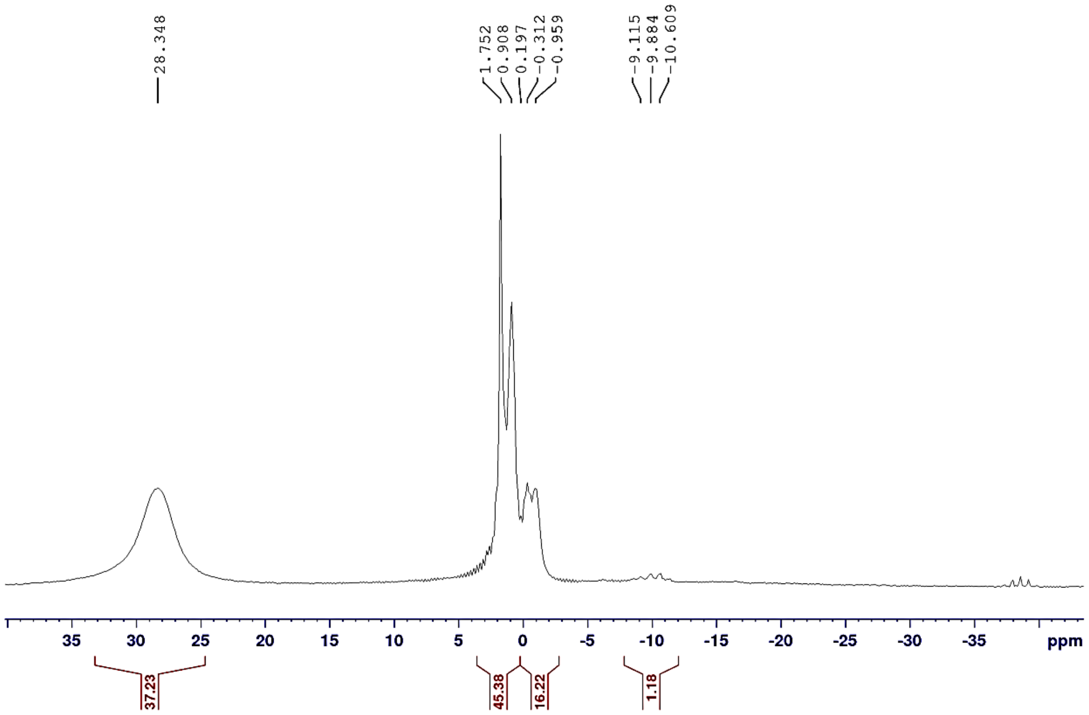

C) ^{13}C NMR spectrum

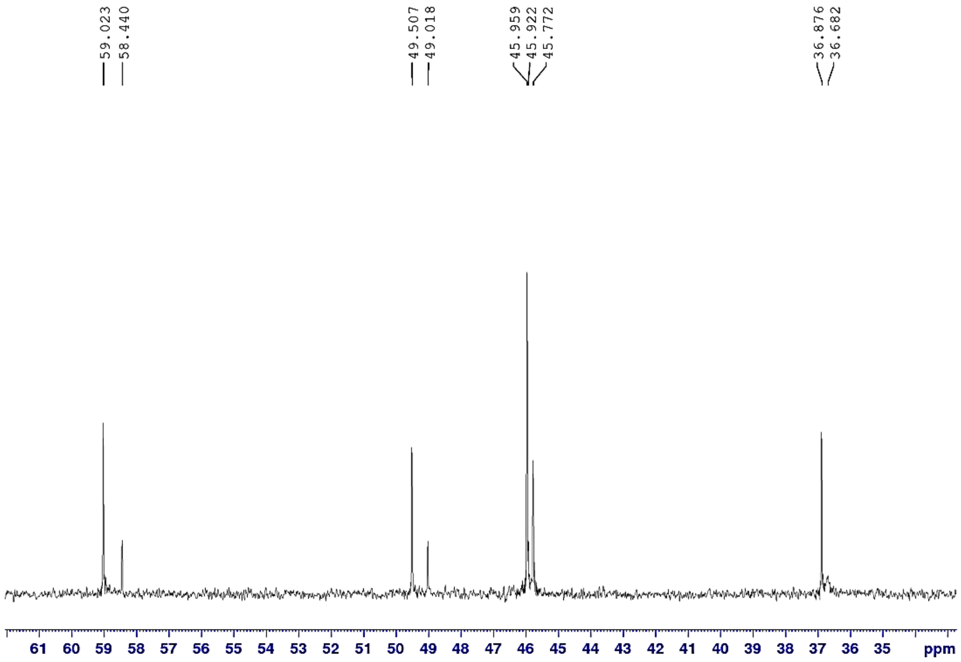

D) HSQC spectrum

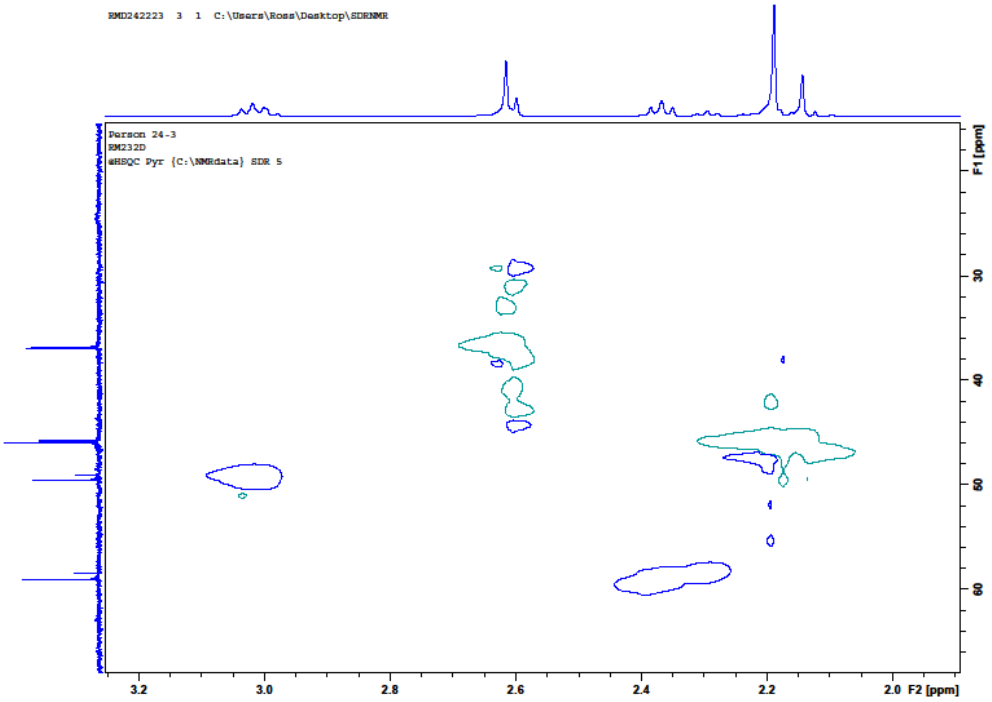

The ^{1}H NMR spectrum indicates the presence of two similar products, in an approximate 3:2 ratio. A heteronuclear ^{1}H-^{13}C HSQC experiment supports this view since there are no cross-peaks between the two components.

E) DOSY spectrum

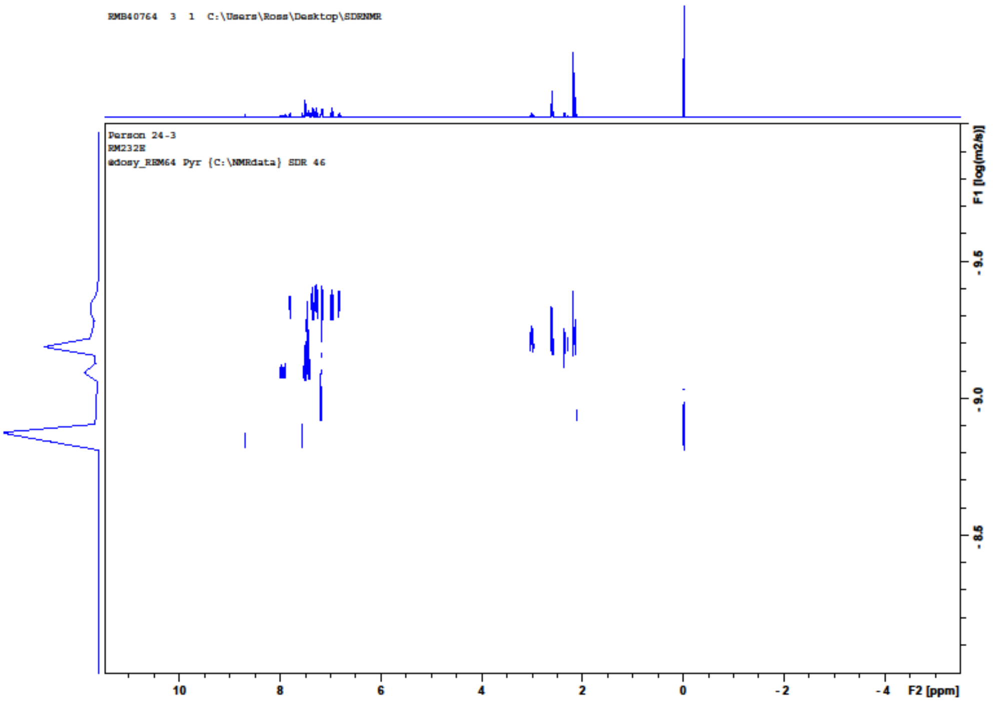

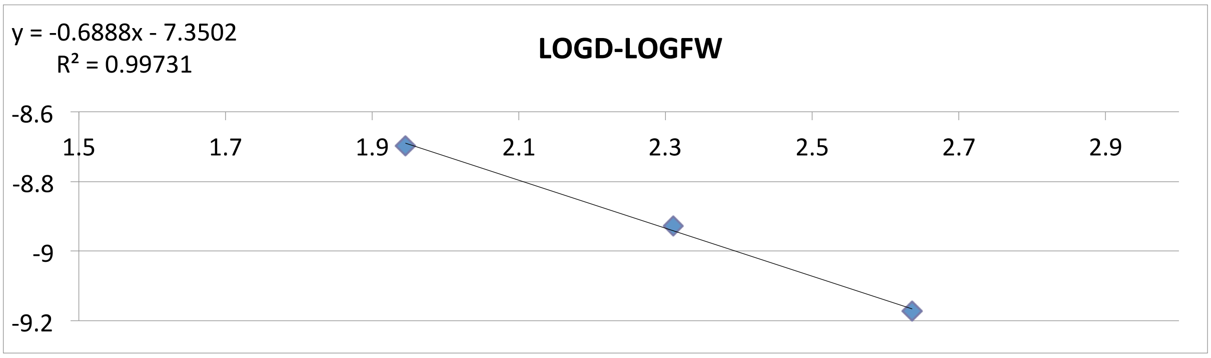

A DOSY experiment reveals the two components possess a similar diffusion coefficient and near identical MWs (258.8 and 258.5 g mol^{-1}). The MW of **[VI·THF]$_2$** without any solvating THF is 243 g mol^{-1}, thus it is reasonable to assume that the two molecules may be isomers of each other and are potentially the result of an intermolecular coupling between two monomers. At this point the identity of the mixture is unclear but the result does again illustrate the profound impact that the reaction solvent has on the process.

Synthesis and NMR characterisation of [VI·py]$_2$

V (116 mg, 1 mmol) was dissolved in hexane (3 mL) and *n*BuLi (0.63 mL, 1 mmol 1.6M in hexane) was added, resulting in precipitation of a white solid after several minutes. After 30 min. stirring, pyridine was added dropwise until a colourless solution was obtained. Crystals suitable for single crystal X-ray diffraction studies were grown after standing the solution at -20 °C for 24 h. Yield 96 mg, 48%.

Elemental analysis (%) calculated for $C_{20}H_{42}N_6B_2Li_2$: C 59.74, H 10.53, N 20.90; found: C 59.31, H 11.04, N 20.96.

^{1}H NMR (400.1 MHz, C_6D_6 300K): δ 8.53 (2H, m, C*H*-Pyr), 6.95 (1H, tt, $^{3}J_{H-H}$ 7.60 Hz; $^{4}J_{H-H}$ 1.80 Hz, C*H*-pyr), 6.64 (2H, m, C*H*-Pyr), 2.71 (3H, br s, CH_3-diamine), 2.56 (2H, br s,CH_2-diamine), 2.43 (2H, br s,CH_2-diamine), 1.98 ppm (1H, s, 2xCH_3-diamine).

^{11}B NMR (128.4 MHz, C_6D_6 300K): δ -17.0 ppm $^{1}J_{B-H}$ 77.6 Hz

^{7}Li NMR (155.5 MHz, C_6D_6 300K): δ 0.96 ppm

^{13}C NMR (100.6 MHz, C_6D_6 300K): δ 150.1 (pyr), 135.7 (pyr), 123.6 (pyr), 58.9 (*C*H_2), 57.6 (*C*H_2), 48.3 (*C*H_3), 45.2 ppm (2x*C*H_3).

Figure S26 NMR characterisation of **[VI·py]$_2$**

A) ^{1}H NMR spectrum

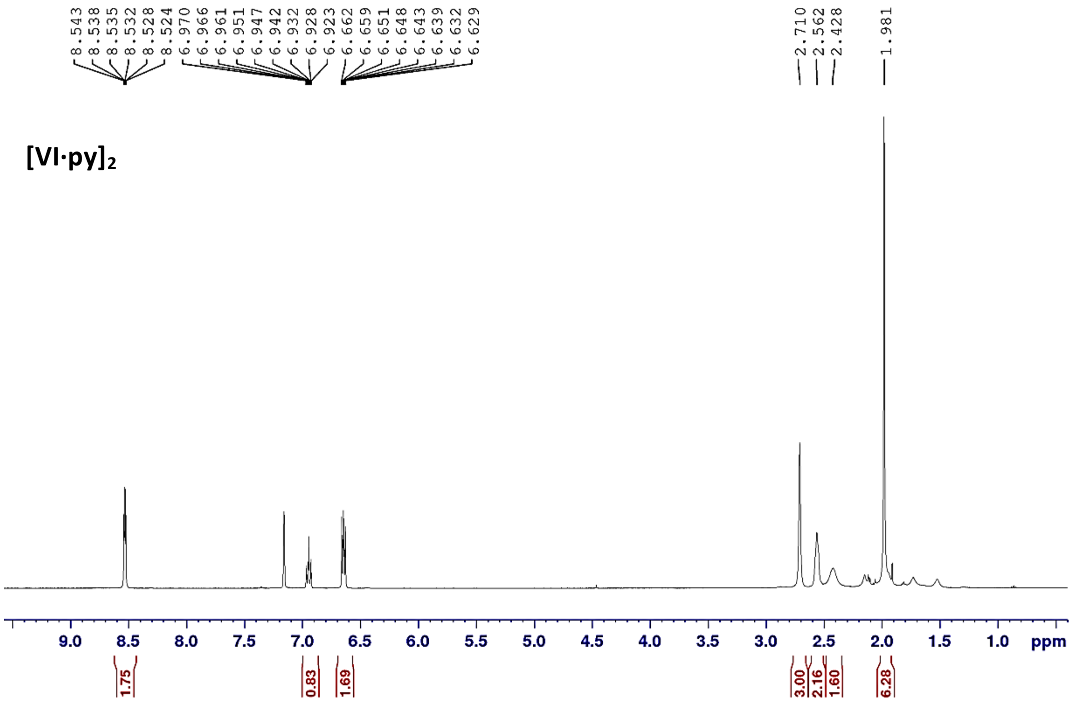

B) ^{7}Li NMR spectrum

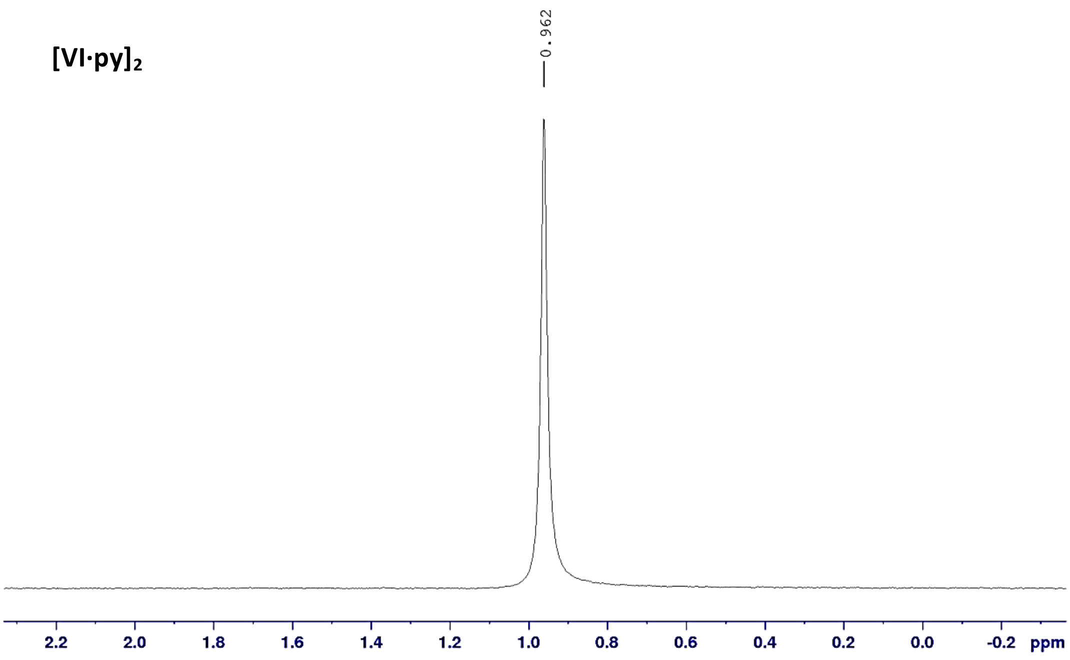

C) ^{11}B NMR spectrum

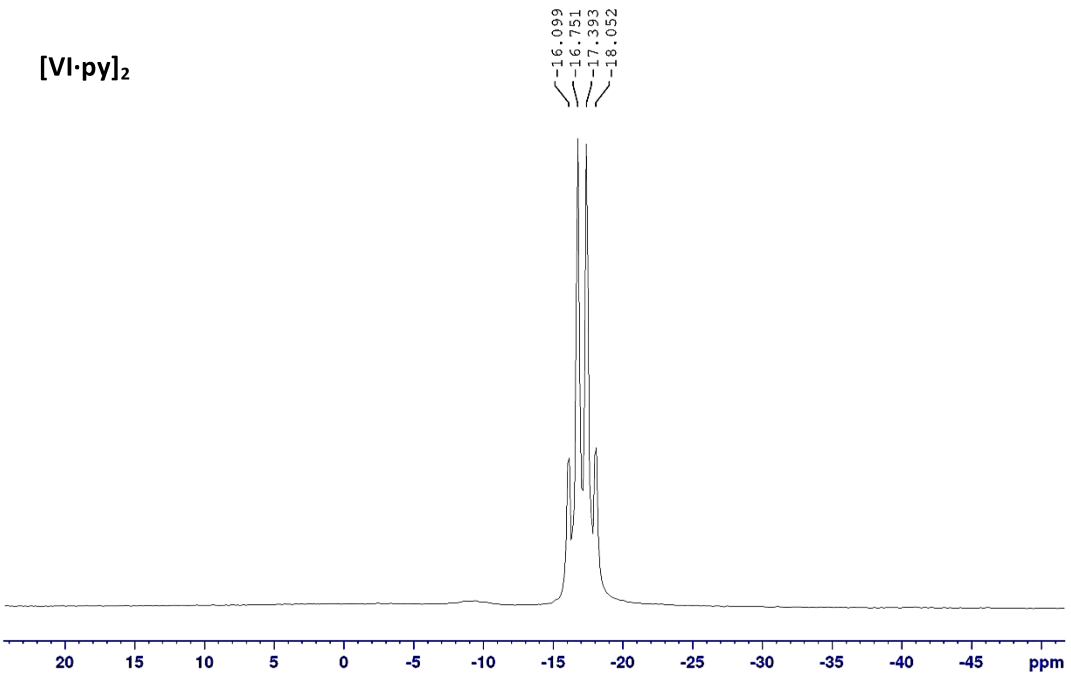

D) ^{13}C NMR spectrum

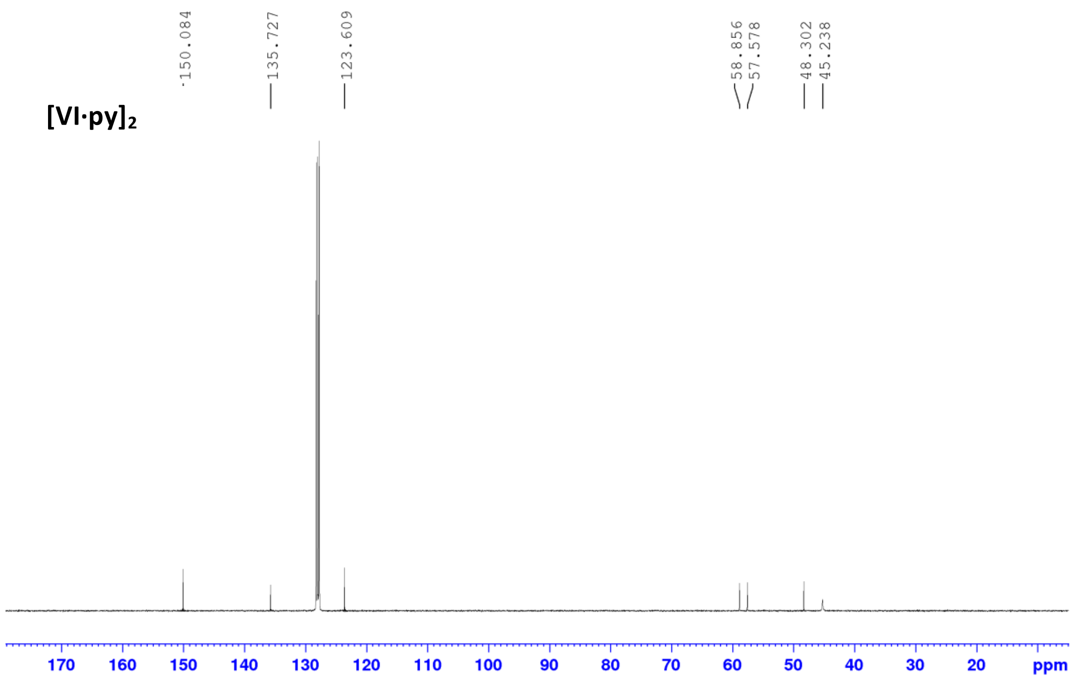

E) Infrared spectrum of **[VI·py]$_2$**

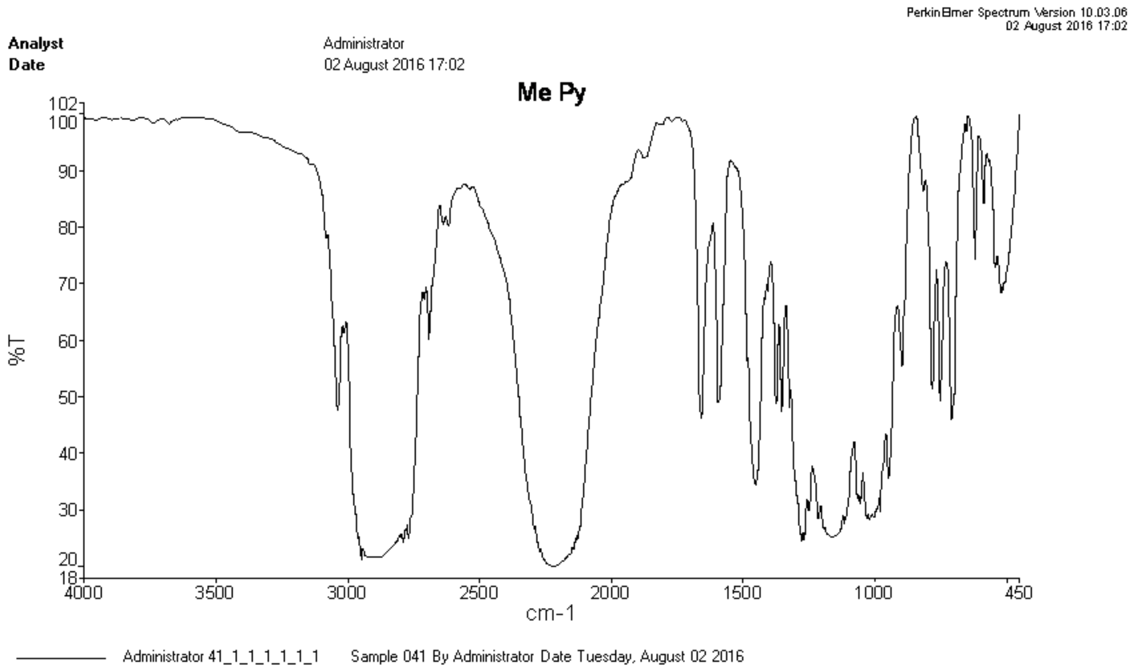

Figure S27 [VI·py]₂ in d_6-benzene at 70 °C for 10 min and 72 h.

A) ^{1}H NMR spectra

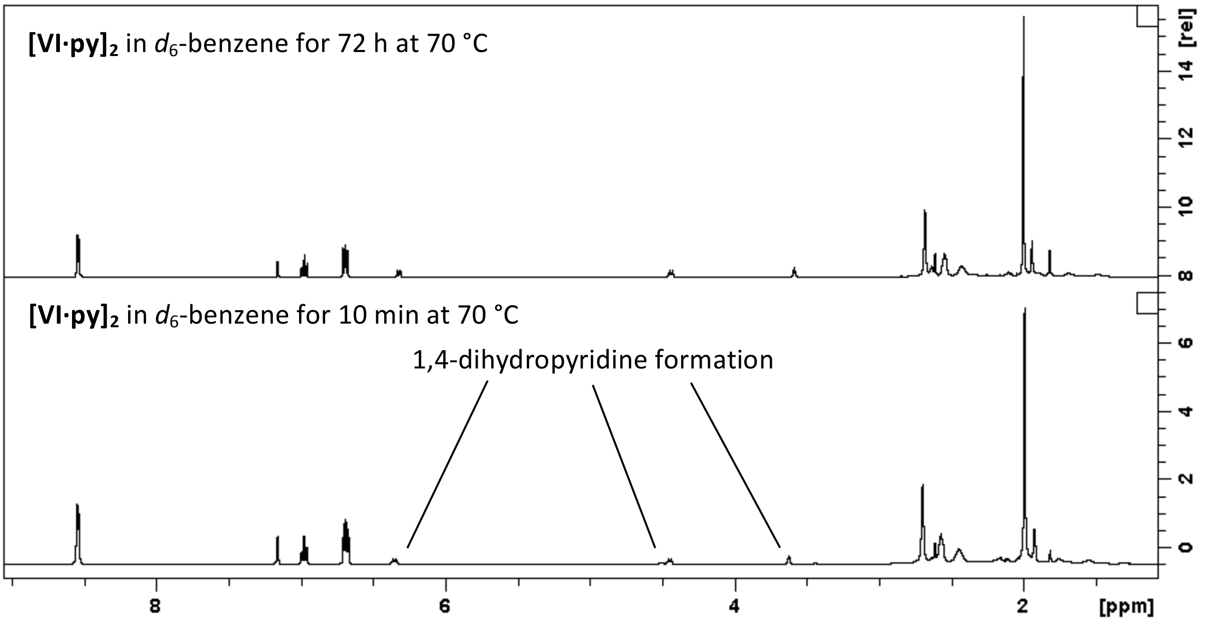

B) ^{11}B NMR spectra

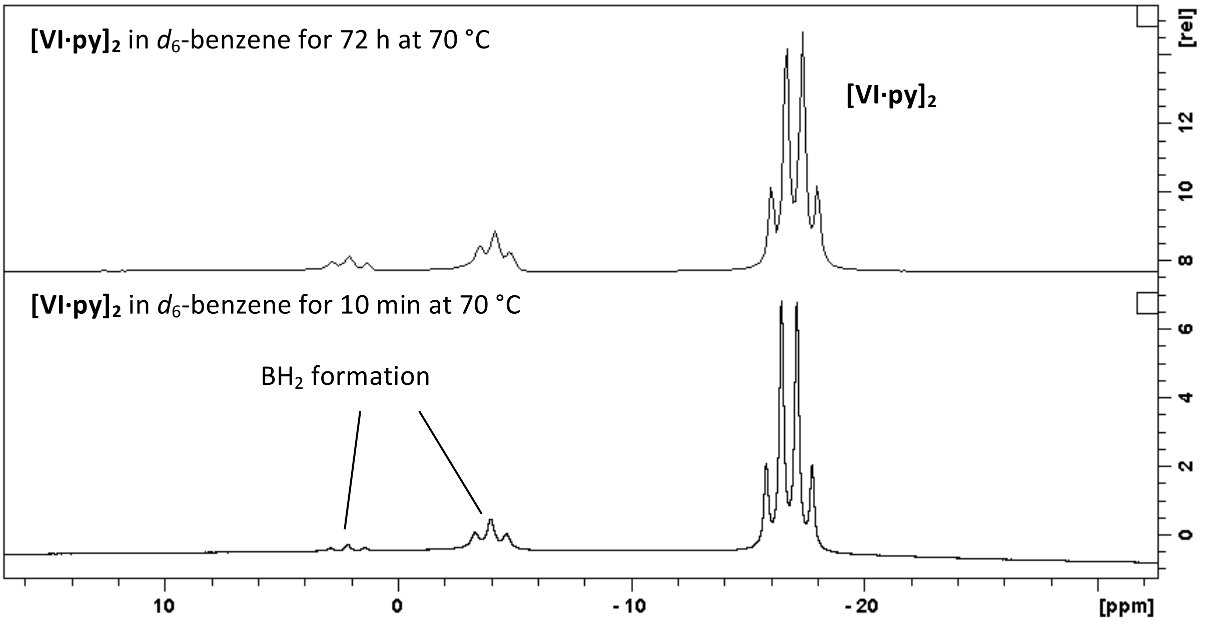

Figure S28 Reaction of **IVa** with PhLi – Formation of **IVaPh**

A) ^{1}H NMR spectrum in d_6-benzene after reacting PhLi and **IVa** (prepared in situ) for 2 hours at room temperature. Indicating formation of **IVaPh**

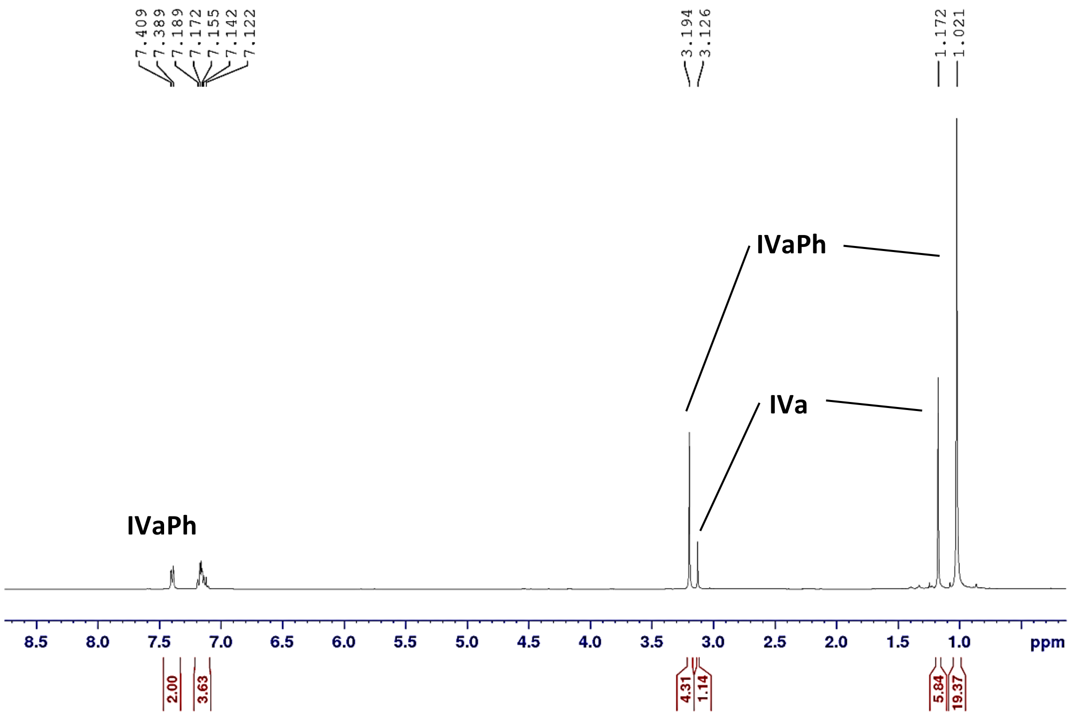

B) ^{11}B NMR spectrum in d_6-benzene after reacting PhLi and **IVa** (prepared in situ) for 2 hours at room temperature.

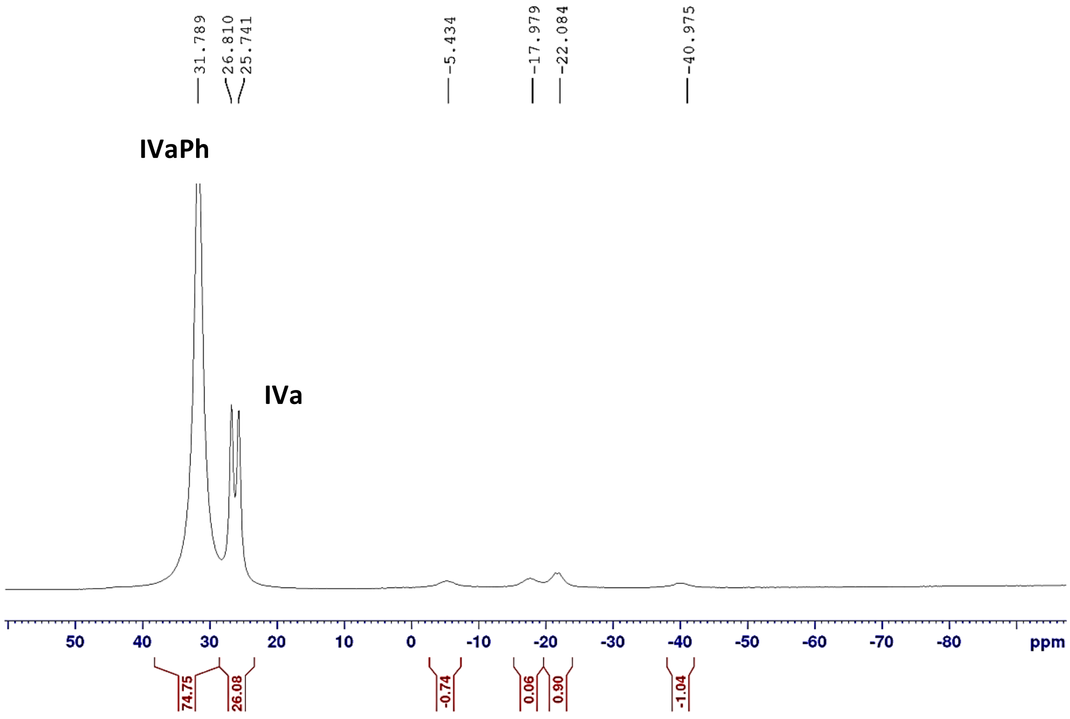

Synthesis and NMR characterisation of IVaPh

Ia (372 mg, 2 mmol) and **1t**Li (14 mg, 5 mol%) were dissolved in toluene (2 mL) and heated at 80 °C for 7h to ensure *in situ* conversion to **IVa**. Phenyllithium (168 mg , 2 mmol) was added and the reaction stirred overnight. Hexane (5 mL) was added and the reaction placed at –70 °C. After 24 hours, colourless crystals suitable for X-ray diffraction studies formed. Yield 361 mg, 70%.

^{1}H NMR (400.1 MHz, C_6D_6 300K): δ 7.42 (2H, m, C*H*-phenyl), 7.20-7.10 (3H, m, C*H*-phenyl), 3.19 (4H, s,CH_2), 1.03 ppm (18H, s, CH_3).

^{11}B NMR (128.4 MHz, C_6D_6 300K): δ 31.8 ppm (s, *B*Ph).

^{13}C NMR (100.6 MHz, C_6D_6 300K): δ 132.7 (*C*-Ph), 127.4 (*C*-Ph), 126.9 (*C*-Ph), 51.8 (quaternary *C*-tBu), 45.2 (*C*H_2), 30.9 ppm (*C*H_3-tBu).

Figure S29 NMR characterisation of **IVaPh**

A) ^{1}H NMR spectrum

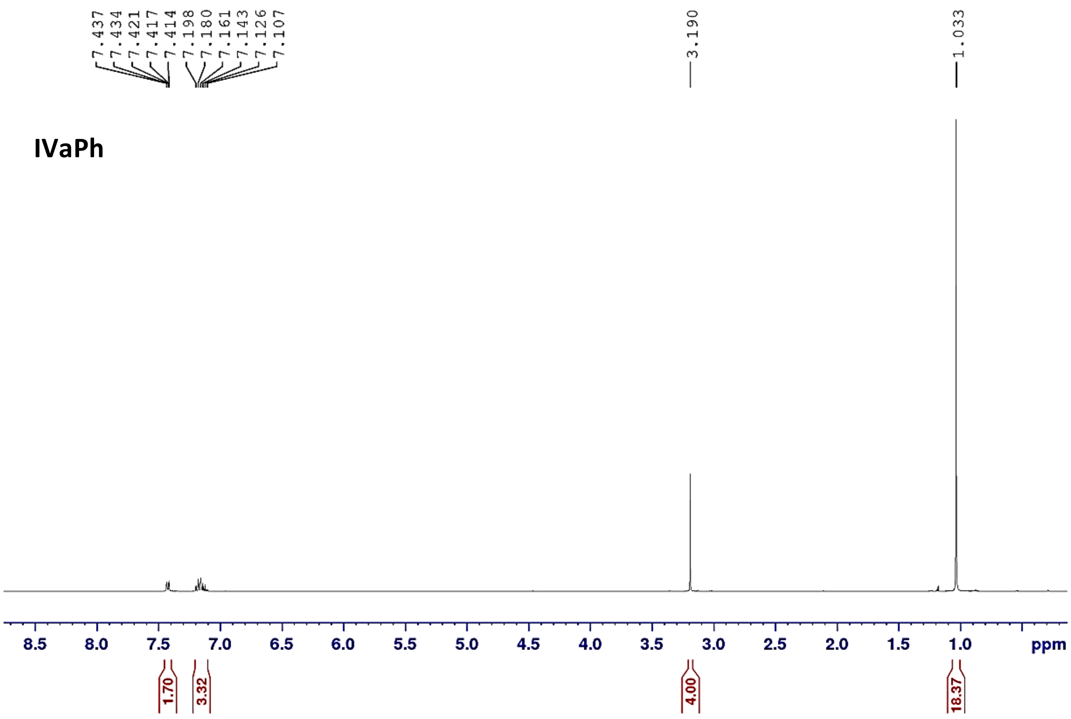

B) ^{11}B NMR spectrum

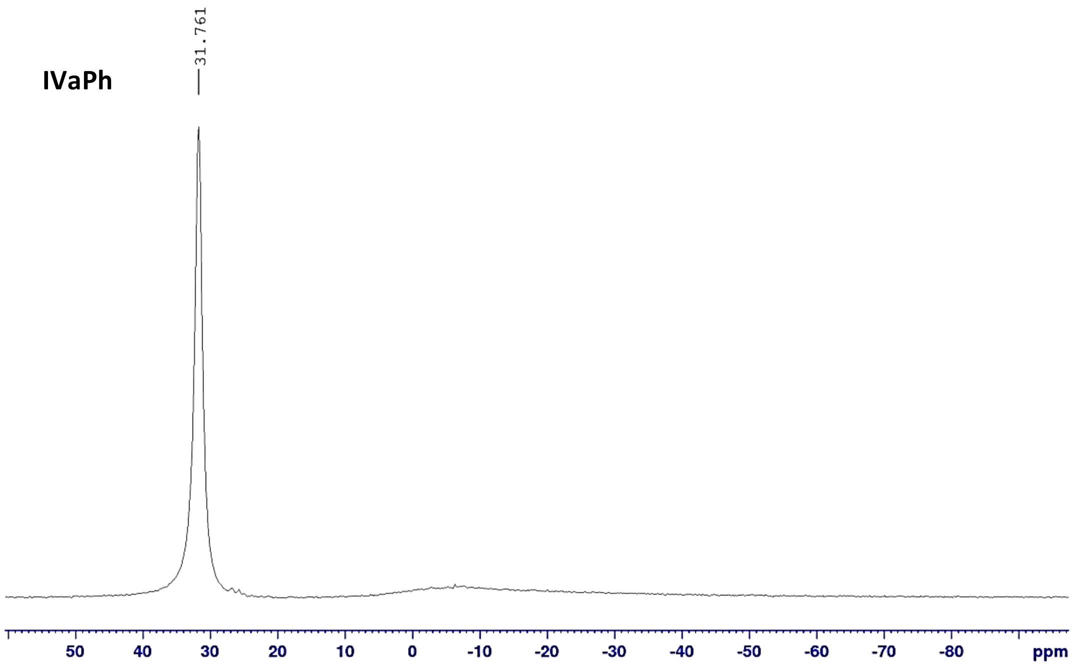

C) ^{13}C NMR spectrum

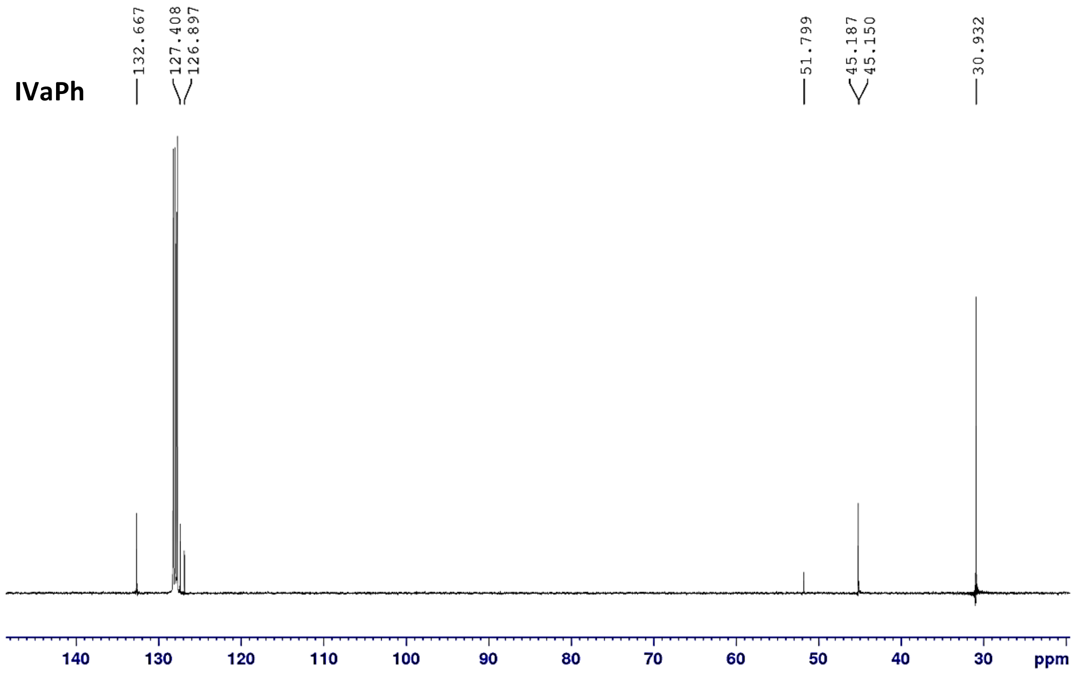

Figure S30 Structural characterisation of **IVaPh** and selected bond parameters.

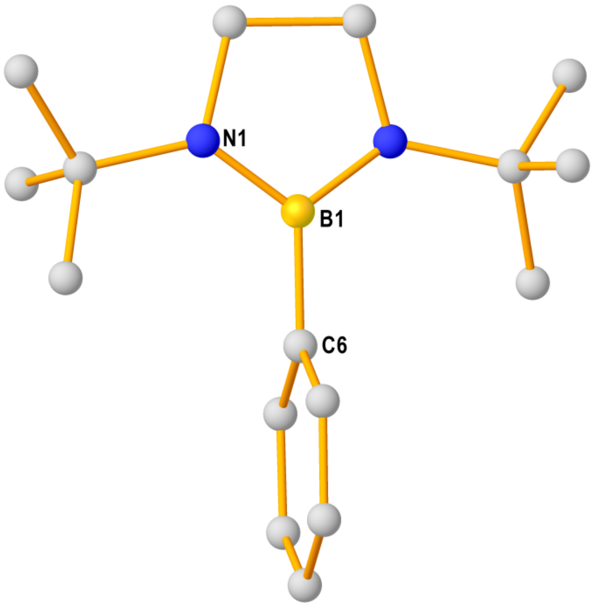

Molecular structure of **IVaPh**, transformations used to generate symmetry equivalent atoms -x,+y,1/2-z. All hydrogen atoms are omitted for clarity. Selected bond lengths (Å) and angles (°): B1-N1, 1.4285(12); B1-C6, 1.578(2); N1-B1-N1', 109.68(12); N1-B1-C6, 125.16(6).

Figure S31 Reaction of **IVb** with PhLi – Formation of **IVbPh**

A) ^{1}H NMR in d_6-benzene after reacting PhLi and **IVb** (prepared in situ) for 2 hours at room temperature. Indicating formation of **IVbPh**

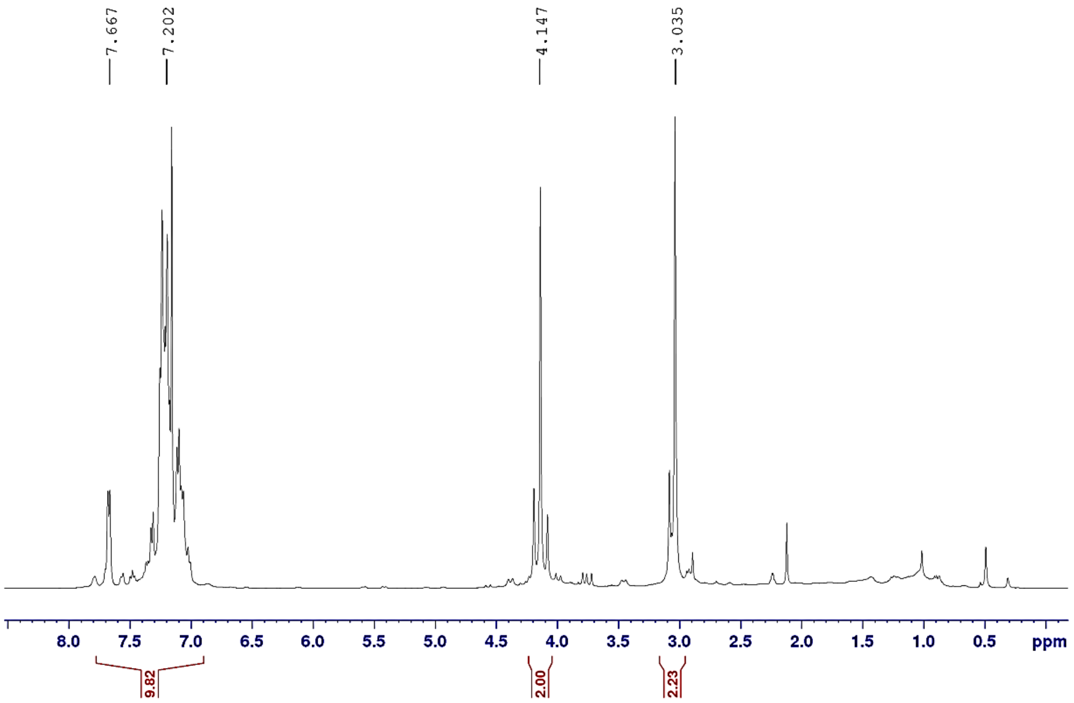

B) ^{11}B NMR in d_6-benzene after reacting PhLi and **IVb** (prepared in situ) for 2 hours at room temperature.

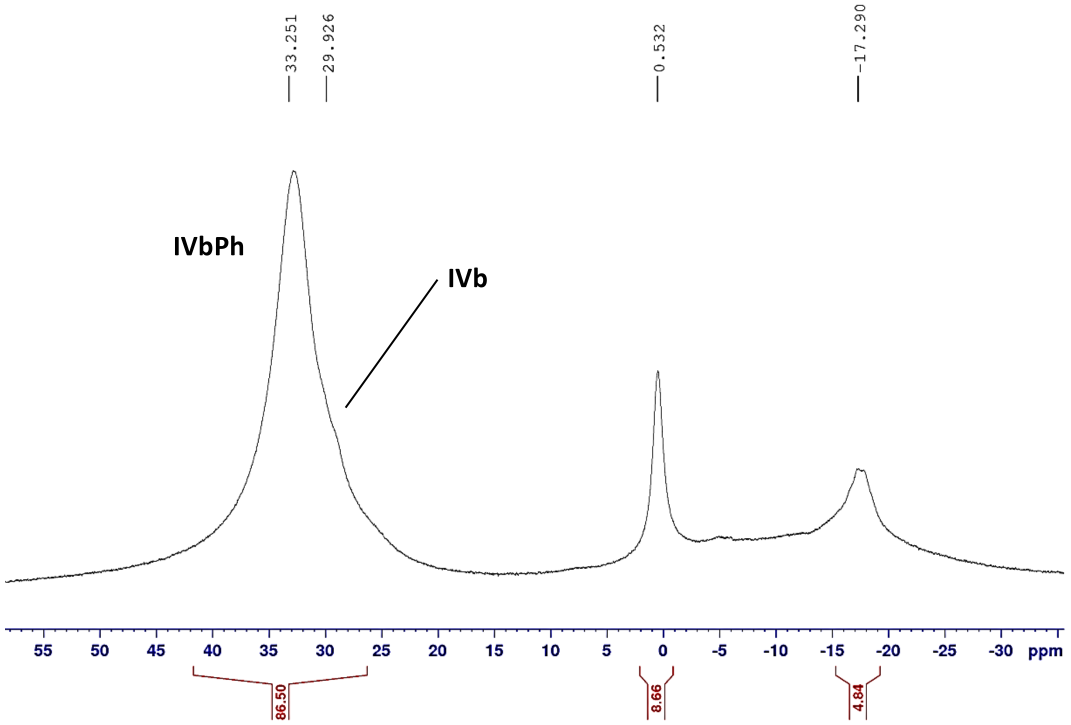

Synthesis and NMR characterisation of IVbPh

Ib (1.640 g, 4 mmol) and **1t**Li (29 mg, 5 mol%) were dissolved in toluene (4 mL) and heated at 80 °C for 24 h to ensure *in situ* conversion to **IVb**. Phenyllithium (336 mg , 4 mmol) was added and the reaction stirred overnight. Hexane (5 mL) was added and the reaction placed at –70 °C. After 24 hours, colourless crystals formed and were isolated by filtration. Yield 1.052 g, 81%.

^{1}H NMR (400.1 MHz, C_6D_6 300K): δ 7.68 (2H, m, C*H*-phenyl), 7.21 (11H, m, C*H*-phenyl), 7.10 (2H, m, C*H*-phenyl), 4.14 (4H, s,C*H*$_2$), 3.03 ppm (4H, s, C*H*$_2$).

^{11}B NMR (128.4 MHz, C_6D_6 300K): δ 32.7 ppm (s, *B*Ph).

^{13}C NMR (100.6 MHz, C_6D_6 300K): δ 141.1 (*C*-Ph), 133.2 (*C*-Ph), 128.8 (*C*-Ph), 128.7 (*C*-Ph), 128.4 (*C*-Ph), 127.6 (*C*-Ph), 126.9 (*C*-Ph), 51.4 (benzyl *C*H$_2$), 48.4 ppm (*C*H$_2$).

Figure S32 NMR characterisation of **IVbPh**

A) ^{1}H NMR spectrum

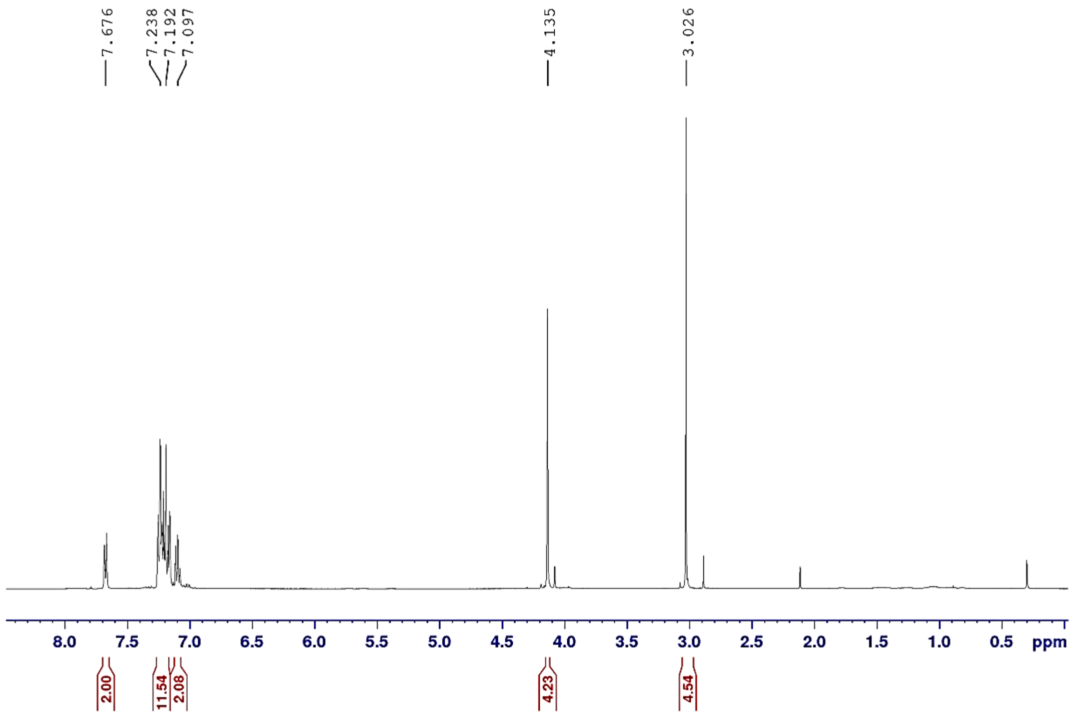

B) ^{11}B NMR spectrum

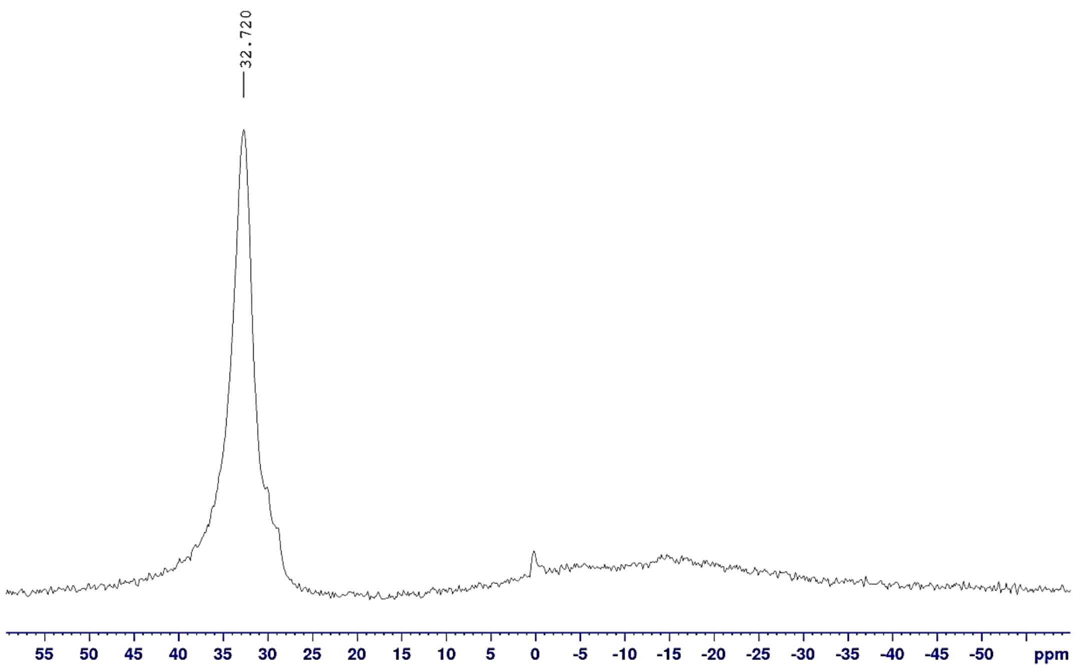

C) ^{13}C NMR spectrum

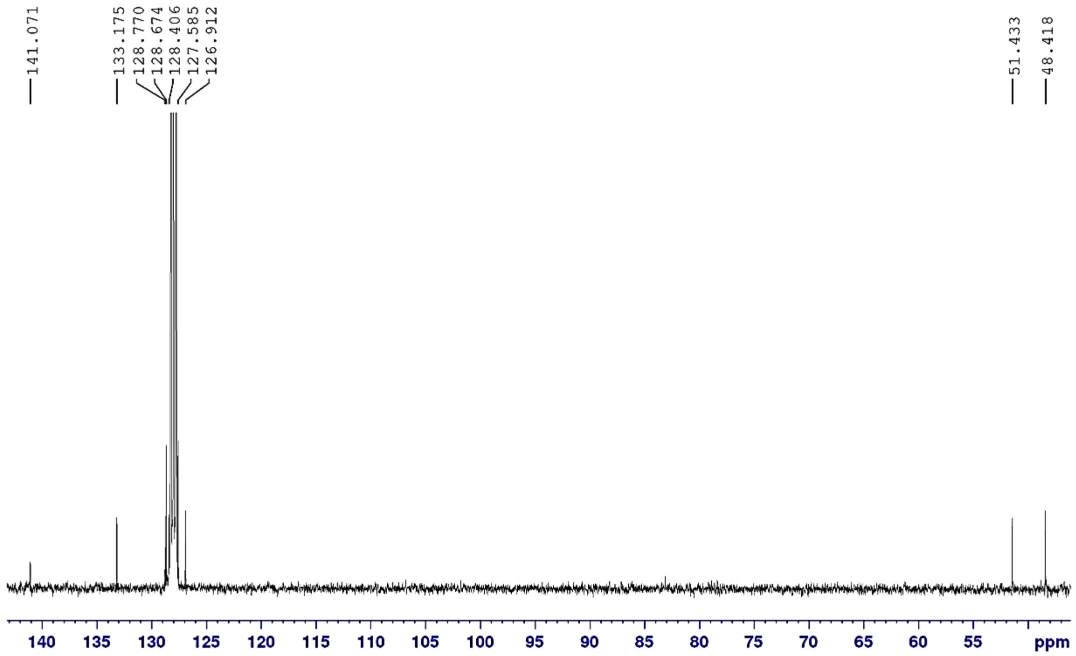

Figure S33 Reaction of **IVc** with PhLi – Formation of **IVcPh**

A) ^{1}H NMR in d_6-benzene after reacting PhLi and **IVc** (prepared in situ) for 2 hours at room temperature. Indicating formation of **IVcPh**

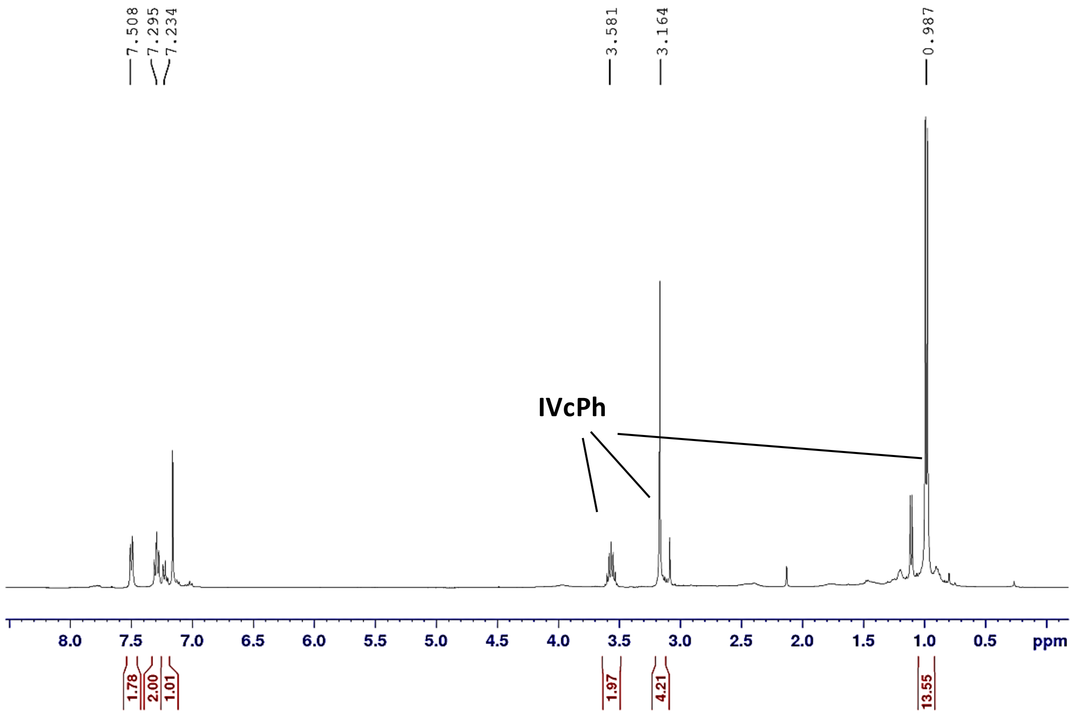

B) ^{11}B NMR in d_6-benzene after reacting PhLi and **IVc** (prepared in situ) for 2 hours at room temperature. Indicating formation of **IVcPh**

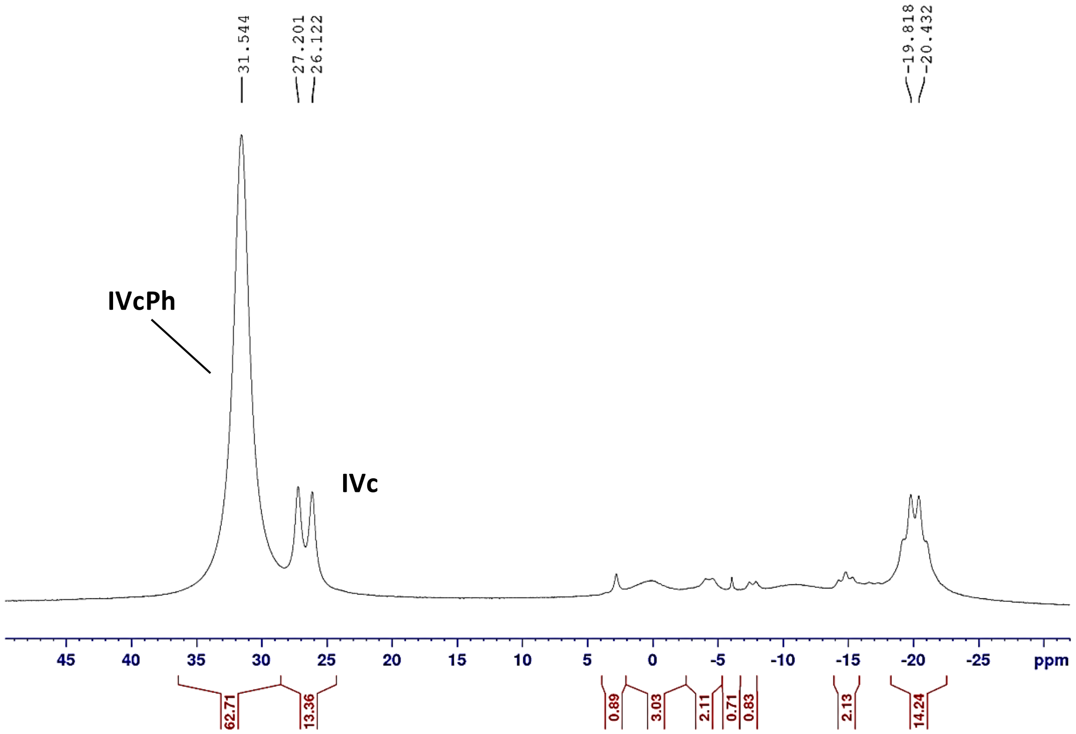

Synthesis and NMR characterisation of IVcPh

Ic (462 mg, 3 mmol) and **1t**Li (21 mg, 5 mol%) were dissolved in toluene (4 mL) and heated at 80 °C for 48 h to ensure *in situ* conversion to **IVc**. Phenyllithium (252 mg , 4 mmol) was added and the reaction stirred overnight. Hexane (5 mL) was added and the reaction placed at –70 °C. After 24 hours, colourless crystals formed. These were isolated at low temperature by decanting the solution from the solid. **IVcPh** exists as a colourless oil at room temperature. Yield 421 mg, 61%.

^{1}H NMR (400.1 MHz, C_6D_6 300K): δ 7.54 (2H, d, C*H*-phenyl), 7.32 (2H, m, C*H*-phenyl), 7.25 (1H, t, C*H*-phenyl), 3.60 (2H, septet, C*H*(CH_3)$_2$), 3.16 (4H, s,C*H*$_2$), 0.98 ppm (12H, d, C*H*(C*H*$_3$)$_2$).

^{11}B NMR (128.4 MHz, C_6D_6 300K): δ 31.6 ppm (s, *B*Ph).

^{13}C NMR (100.6 MHz, C_6D_6 300K): δ 132.9 (*C*-Ph), 128.1 (*C*-Ph), 126.9 (*C*-Ph), 45.2 (*C*H$_2$), 41.9 (*C*H-CH$_3$)$_2$), 22.0 ppm (CH(*C*H$_3$)$_2$).

Figure S34 NMR characterisation of **IVcPh**

A) ^{1}H NMR spectrum

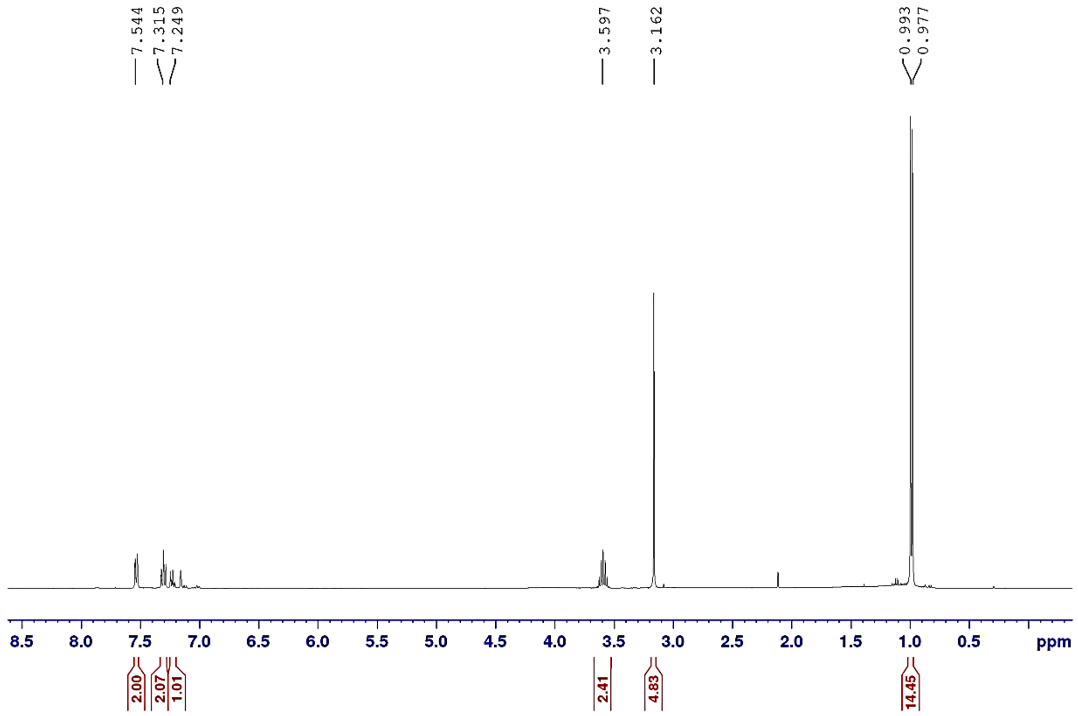

B) ^{11}B NMR spectrum

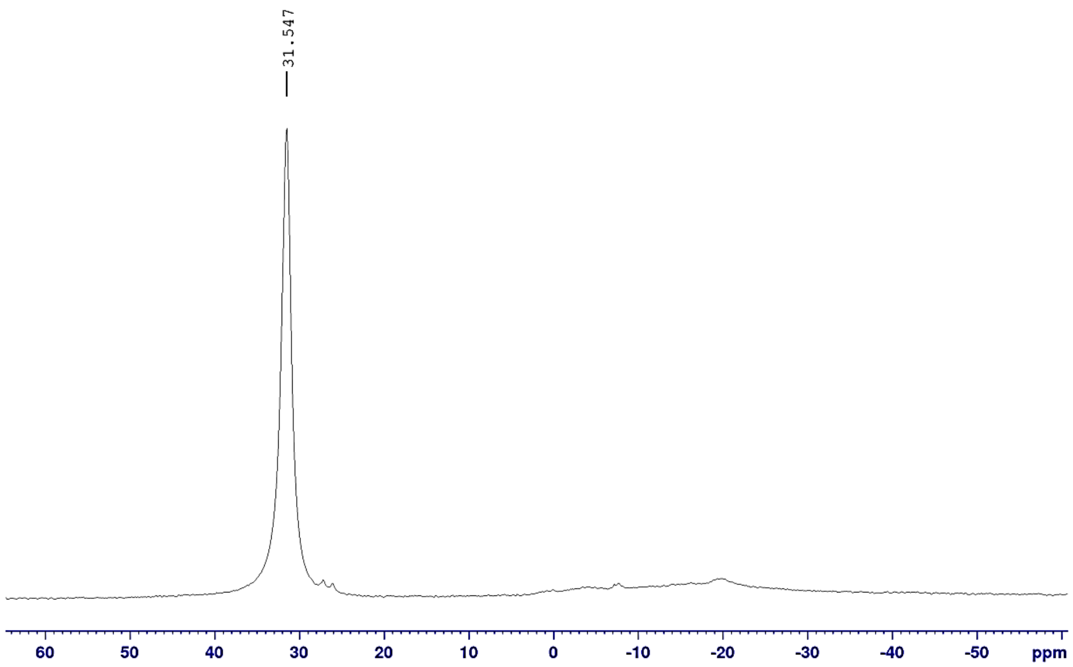

C) ^{13}C NMR spectrum

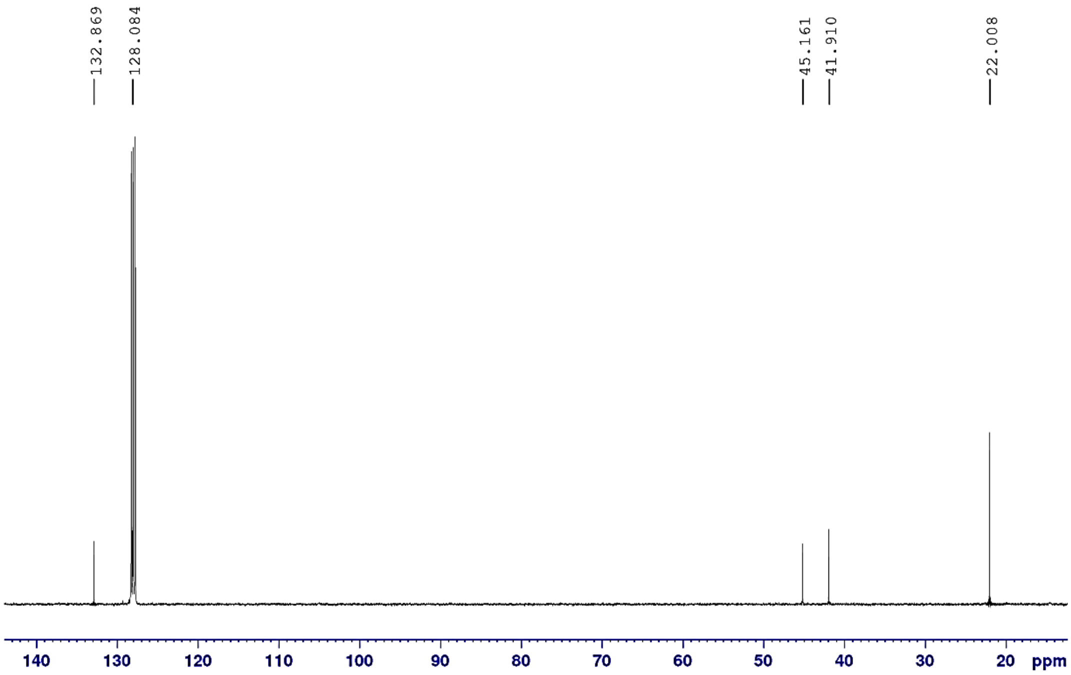

Figure S35 Reaction of **IVd** with PhLi – Formation of **IVdPh**

A) ^{1}H NMR in d_6-benzene after reacting PhLi and **IVd** (prepared in situ) for 2 hours at room temperature. Indicating formation of **IVdPh**

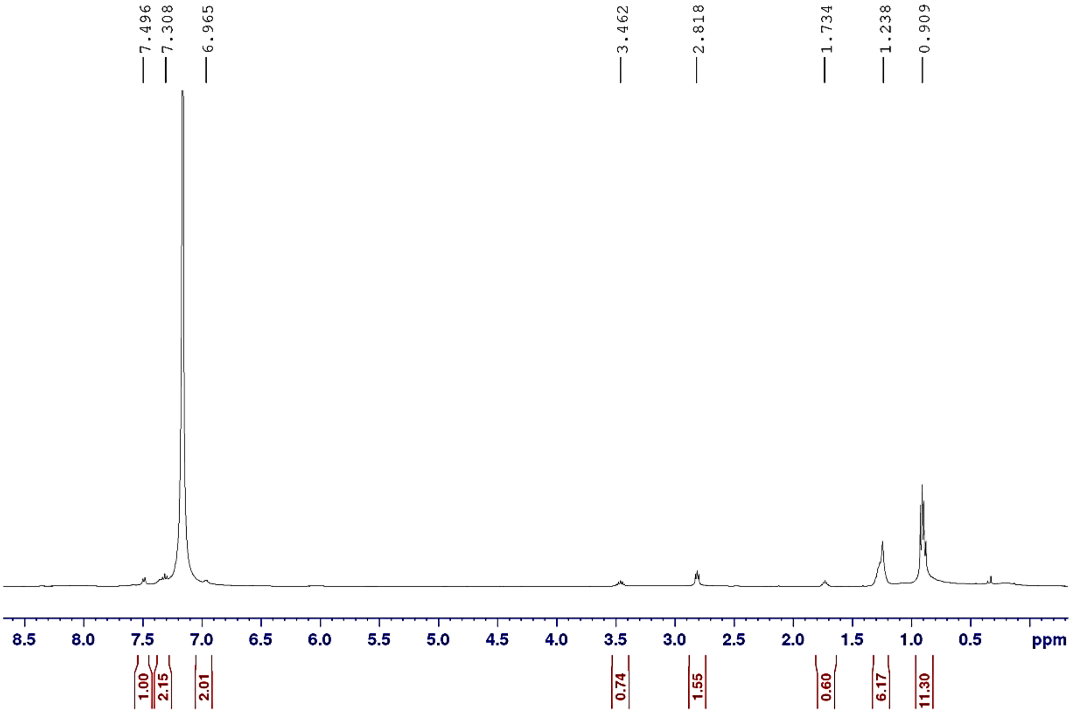

B) ^{11}B NMR in d_6-benzene after reacting PhLi and **IVd** (prepared in situ) for 2 hours at room temperature. Indicating formation of **IVdPh**

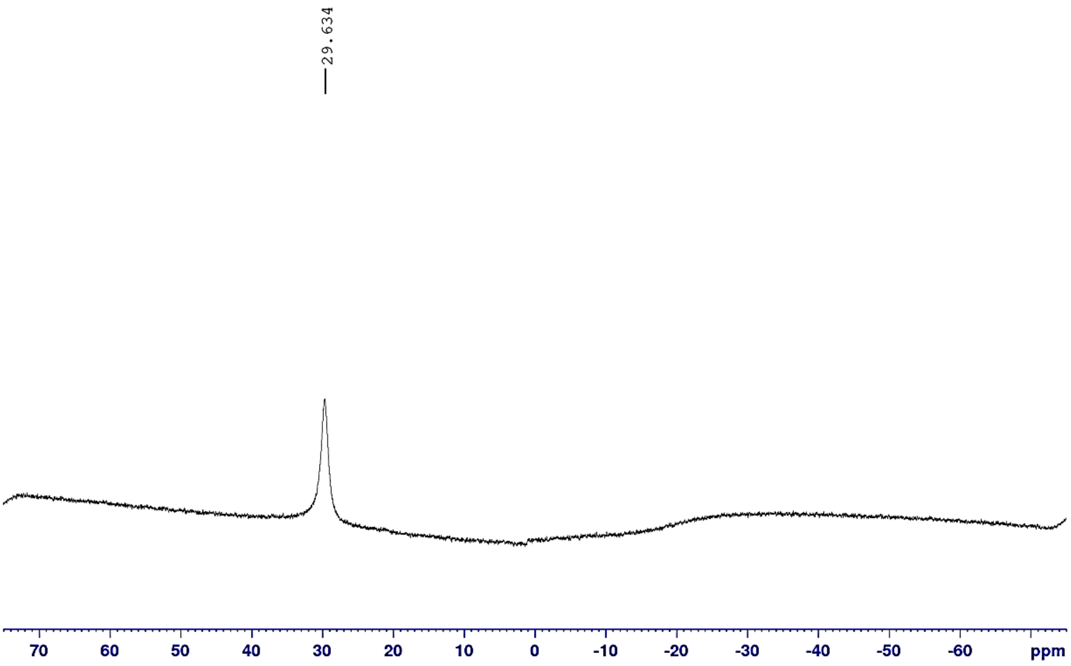

Synthesis and NMR characterisation of IVdPh

Id (420 mg, 2.5 mmol) and **1t**Li (18 mg, 5 mol%) were dissolved in toluene (4 mL) and heated at 80 °C for 48 h to ensure *in situ* conversion to **IVd**. Phenyllithium (156 mg , 2.5 mmol) was added and the reaction stirred overnight. Hexane (5 mL) was added and the reaction placed at –70 °C. After 24 hours a white solid formed. These were isolated at low temperature by decanting the solution from the solid. The reaction product mixture exists as a waxy white solid in a 3:1 ratio of **IVcPh:IVc**. Combined yield 421 mg, 38%.

Figure S36 NMR characterisation of **IVdPh/IVd** product mixture

A) ^{1}H NMR spectrum

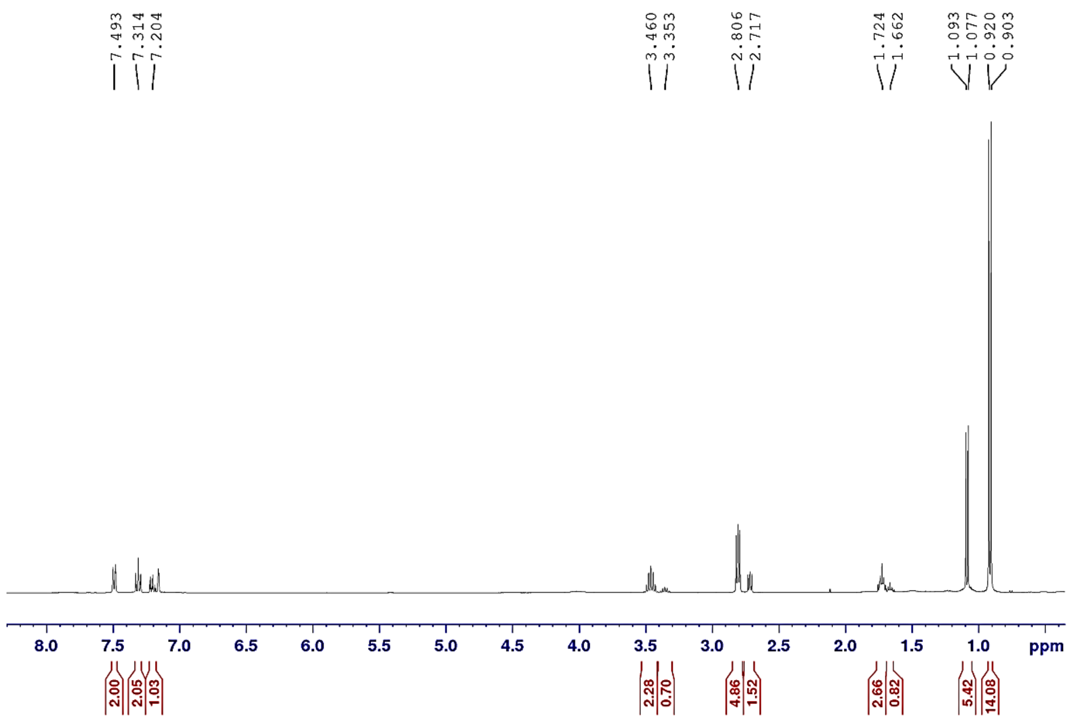

B) ^{11}B NMR spectrum

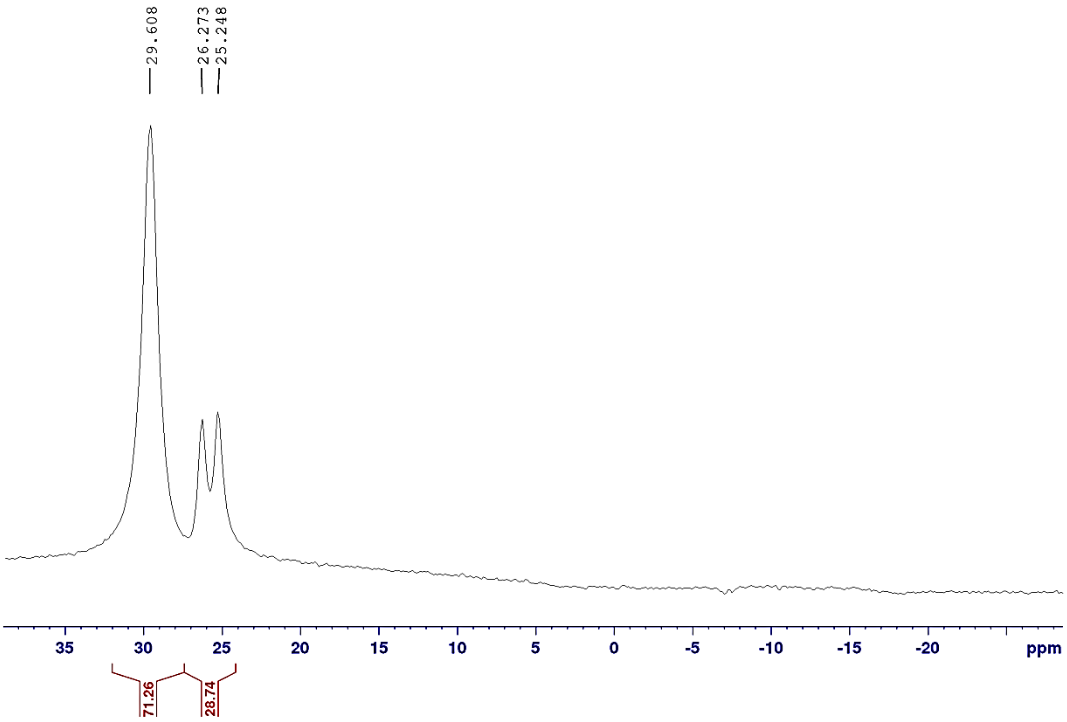

Table S1 Crystallographic data and refinement details for complexes IIa·THF, IIa·py, [VI·THF]$_2$, [VI·py]$_2$ and IVaPh

	**IIa·THF**	**IIa·py**	**[VI·THF]$_2$**	**[VI·py]$_2$**	**IVaPh**
Empirical formula	$LiON_2C_{14}BH_{34}$	$LiN_3C_{15}BH_{31}$	$Li_2O_2N_4C_{18}B_2H_{48}$	$Li_2B_2N_6C_{20}H_{42}$	$C_{16}H_{27}B_1N_2$
Mol. Mass	264.18	271.18	388.10	402.09	258.2
Crystal system	monoclinic	Monoclinic	Triclinic	Monoclinic	Monoclinic
a/ Å	6.1321(6)	8.4263(19)	8.4191(6)	8.4964(11)	14.5259(12)
b/ Å	15.8714(15)	6.2044(9)	8.8743(7)	9.8471(10)	10.3281(7)
c/ Å	18.017(2)	16.977(3)	9.7883(8)	15.8260(19)	11.2033(10)
α	90	90	67.501(8)	90	90
β	93.743(10)	100.745(19)	72.307(7)	104.836(12)	110.814(10)
γ	90	90	72.253(7)	90	90
V/ Å^3	1749.7(3)	872.0(3)	628.76(10)	1279.9(3)	1571.1(2)
Z	4	2	1	2	4
λ/ Å	0.71073	0.71073	0.71073	0.71073	0.71073
Measured reflections	17408	6136	12110	12683	3789
Unique reflections	4579	3083	3436	3477	1906
R_{int}	0.0512	0.0704	0.0257	0.0248	0.0151
Observed rflns [$I>2\sigma(I)$]	3317	2109	2911	2965	1636
GooF	1.038	1.061	1.111	1.049	1.027
R [on *F*, obs rflns only]	0.0668	0.0753	0.0401	0.0419	0.0426
ωR [on F^2, all data]	0.1750	0.2117	0.1461	0.1150	0.1158
Largest diff. Peak/hole. e/ Å^{-3}	0.37/-0.23	0.29/-0.21	0.31/-0.18	0.34/-0.24	0.41/-0.20

1. S. D. Robertson, A. R. Kennedy, J. J. Liggat, R. E. Mulvey, *Chem. Commun*. **2015**, *51*, 5452–5455.

2. C. J. Wallis, G. Alcaraz, A. S. Petit, A. I. Poblador-Bahomande, E. Clot, C. Bijani, L. Vendier, S. Sabo-Etienne, *Chem*. *Eur*. *J*. **2015**, *21*, 13080-13090.

3. G. M. Sheldrick, *Acta Crystallogr*. **2007**, A64, 112-122.

4. O. V. Dolomanov; L. J. Bourhis; R. J. Gildea; J. A. K. Howard; H. Puschmann, *J. Appl. Cryst*. **2009**, *42*, 339-341.
